# Extensive crop–wild hybridization during *Brassica* evolution and selection during the domestication and diversification of *Brassica* crops

**DOI:** 10.1093/genetics/iyad027

**Published:** 2023-02-22

**Authors:** Jasmine M Saban, Anne J Romero, Thomas H G Ezard, Mark A Chapman

**Affiliations:** Biological Sciences, University of Southampton, Life Sciences Building, Highfield Campus, Southampton, SO17 1BJ, UK; Biological Sciences, University of Southampton, Life Sciences Building, Highfield Campus, Southampton, SO17 1BJ, UK; Ocean and Earth Science, National Oceanography Centre Southampton, Southampton, SO14 3ZH, UK; Biological Sciences, University of Southampton, Life Sciences Building, Highfield Campus, Southampton, SO17 1BJ, UK

**Keywords:** *Brassica*, domestication, crop wild relatives, introgression, phylogenomics, Plant Genetics and Genomics

## Abstract

Adaptive genetic diversity in crop wild relatives (CWRs) can be exploited to develop improved crops with higher yield and resilience if phylogenetic relationships between crops and their CWRs are resolved. This further allows accurate quantification of genome-wide introgression and determination of regions of the genome under selection. Using broad sampling of CWRs and whole genome sequencing, we further demonstrate the relationships among two economically valuable and morphologically diverse *Brassica* crop species, their CWRs, and their putative wild progenitors. Complex genetic relationships and extensive genomic introgression between CWRs and *Brassica* crops were revealed. Some wild *Brassica oleracea* populations have admixed feral origins; some domesticated taxa in both crop species are of hybrid origin, while wild *Brassica rapa* is genetically indistinct from turnips. The extensive genomic introgression that we reveal could result in false identification of selection signatures during domestication using traditional comparative approaches used previously; therefore, we adopted a single-population approach to study selection during domestication. We used this to explore examples of parallel phenotypic selection in the two crop groups and highlight promising candidate genes for future investigation. Our analysis defines the complex genetic relationships between *Brassica* crops and their diverse CWRs, revealing extensive cross-species gene flow with implications for both crop domestication and evolutionary diversification more generally.

## Introduction

Large crop losses are predicted under future climate change scenarios (Challinor *et al*. 2014; Mbow *et al*. 2019), presenting significant challenges to ensuring food security and human health (Nelson *et al*. 2018; Mbow *et al*. 2019). Crop domestication generally results in a reduction of genetic diversity because of strong selection and limited population sizes (Gaut *et al*. 2018). Crops can therefore lack the genetic variation needed to rapidly adapt to environmental change (Zhang *et al*. 2017). Crop wild relatives (CWRs) may contain adaptive variants that can be exploited for crop improvement through selective breeding and the potential for phenotypic plasticity (Bailey-Serres *et al*. 2019). Indeed, in some crops, natural introgression of adaptive alleles from wild relatives may have already facilitated the cultivation of early domesticates in novel environments (Janzen *et al*. 2019). Understanding phylogenetic relationships between crops and CWRs and the extent of hybridization throughout domestication are vital to determine how evolutionary potential might aid future breeding programs.

*Brassica oleracea* L. (including cabbage, Brussels sprouts, Chinese kale, cauliflower, broccoli) and *Brassica rapa* L. (turnips, Chinese cabbage, pak choy, bok choy, yellow sarson, among others) are popular vegetables worldwide. Consumption of *Brassica*s is also actively promoted for nutritional benefits because of their high fiber and phytonutrient contents (Kaur *et al*. 2007; Francisco *et al*. 2017). While global consumption of *Brassica* crops is expected to increase, substantial yield losses are predicted due to climate change, pests, and diseases (Rodriguez *et al*. 2015; Phophi and Mafongoya 2017). *Brassica* wild relatives can provide adaptive genetic variation relevant to *Brassica* crop breeding (Branca and Cartea 2011), but some are endangered and poorly represented in seed banks (Branca and Tribulato 2011; Castañeda-Álvarez *et al*. 2016). Efforts to establish phylogenetic relationships have been challenging: wild *Brassica* species display considerable morphological diversity (Snogerup *et al*. 1990; Widen *et al*. 2002) and combinations of *Brassica* species readily hybridize in controlled crosses (FitzJohn *et al*. 2007). Wild populations of *B. oleracea* and *B. rapa* have been identified throughout their predicted native ranges, but, even for these well-studied species, phylogenetic relationships to the crops are not fully understood (Maggioni *et al*. 2018) and the inferred relationships suggest that some “wild” populations are derived from feral populations rather than wild ancestors (Mittell *et al*. 2020; Mabry *et al*. 2021; McAlvay *et al*. 2021).

Several recent analyses have analyzed the genetic relationships between domesticated types (e.g. Cheng, Sun, *et al*. 2016; Guo *et al*. 2021; Cai *et al*. 2022); however, only a few phylogenetic analyses have included wild *Brassica* relatives, and these have used transcriptome, reduced representation, or chloroplast DNA sequencing (Arias and Pires 2012; An *et al*. 2019; Mabry *et al*. 2021; McAlvay *et al*. 2021). The most recent of these analyses have suggested that *Brassica cretica* is likely the closest wild relative of *B. oleracea*, but samples labelled as *B. cretica* were not monophyletic and some individuals were nested in the domesticated groups (Mabry *et al*. 2021) raising outstanding questions for several CWRs to determine their true ancestry, hybridization history, and taxonomic groupings. Further, Mabry *et al*. (2021) demonstrate that putatively wild *B. oleracea* populations are instead feral crop derivatives and not progenitors (see also Mittell *et al*. 2020). For *B. rapa*, some wild populations may well be true wild progenitors, while others appear to be feral escapes from cultivation (McAlvay *et al*. 2021). Both of these most recent analyses (Mabry *et al*. 2021; McAlvay *et al*. 2021) indicate that crop–wild hybridization has occurred in the evolution of some domesticated groups in both species.

Since *B. oleracea* and *B. rapa* are also excellent evolutionary models of convergent evolution due to selection during domestication for parallel phenotypes, selection analyses have compared domesticated populations to identify putative targets of selection (e.g. Cheng, Wu, *et al*. 2016). Signatures of selection within each species alongside parallel selection pressures for the same phenotype have revealed several candidate genes that may play important roles in determining these phenotypes, despite the possibility that extensive hybridization within and between groups could mask true signatures of selection and/or give false signals of selection.

Here, we combine newly generated and existing whole genome sequencing (WGS) data to (1) provide stronger evidence for species relationships among cultivated Brassicas and their suspected CWRs, (2) determine the extent of CWR–crop introgression, (3) resolve the taxonomic status of putative progenitor taxa, and (4) explore the role of hybridization in the emergence of domesticates. We therefore also take this opportunity to (5) further identify genomic regions of recent positive selection in domesticated varieties (and to compare selection targets across species convergently domesticated for similar morphologies), with value to crop breeding efforts.

## Materials and methods

### Whole genome resequencing, data acquisition, and processing

Seeds were obtained for 22 wild *Brassica* accessions: eight wild *B. oleracea* accessions, eight wild *B. rapa* accessions, and six CWRs. These were obtained from the Warwick UK Vegetable Genebank (https://warwick.ac.uk/fac/sci/lifesci/wcc/gru/genebank/seed/), the U.S. National Plant Germplasm System (https://npgsweb.ars-grin.gov/gringlobal/search), and the Leibniz Institute of Plant Genetics and Crop Plant Research Genebank (https://www.ipk-gatersleben.de/en/genebank/). Seeds were grown in the University of Southampton glasshouse and DNA was extracted from frozen leaf material using a modified CTAB protocol (Doyle and Doyle 1990). Novogene Bioinformatics Institute (Cambridge, UK) performed library preparation and 150 bp paired-end (PE) sequencing (350 base insert size) using an Illumina 2500 platform (Illumina, USA). The genome size of the six wild *Brassica* relative species was determined using flow cytometry by Plant Cytometry Services (http://www.plantcytometry.nl/).

Additional resequencing reads were obtained for 86 diploid samples from previously published data sets (see Methods S1) and included *Raphanus raphanistrum* and *Erucastrum elatum* as outgroups. Accession information for all 108 samples is available in Supplementary Table 1.

WGS and acquired resequencing data were quality checked using FastQC (Andrews 2010). Sequences were trimmed and filtered with Trimmomatic v0.36 (Bolger *et al*. 2014), removing adapters, the first five bases, and leading and trailing bases with quality < 5 and where the average quality per base of a sliding window dropped below 15. Reads < 40 bp were removed. Data obtained from An *et al*. (2019) and Kiefer *et al*. (2019) were already trimmed. Following quality control, samples had an average of 11.3× coverage ± 1.4 (95% CI).

### Alignment and SNP filtering

*Brassica* CWRs were mapped more efficiently to the *B. oleracea* pangenome than the *B. rapa ssp. pekinensis* v3.0 genome (Supplementary Table 2). Thus, for initial phylogenetic analysis, all 108 samples were aligned to *B. oleracea* (Golicz *et al*. 2016) using Bowtie2 v2.3.1 (Langmead and Salzberg 2012). For further phylogenetic analysis of *B. rapa* and related *Brassicas*, a subset was aligned to the *B. rapa* ssp. *pekinensis* genome. Only whole chromosome alignments were subsequently analyzed.

Bam files were processed with Picard v2.8.3 (picard.sourceforge.net) and variants detected using the Genome Analysis Toolkit v3.7 (GATK) (Van der Auwera *et al*. 2013) as detailed in Methods S1. Filtering parameters were determined following examination of their distribution in the raw SNP and indel data sets (Supplementary Figs. 10-13). Linkage disequilibrium (LD) decay was calculated using PopLDdecay v3.40 (Zhang *et al*. 2019). SNPs in the two data sets were annotated using SNPeff v5.0 (Cingolani *et al*. 2012) according to annotation files available for genomes.

### SNP phylogenies

**Fig. 1.**
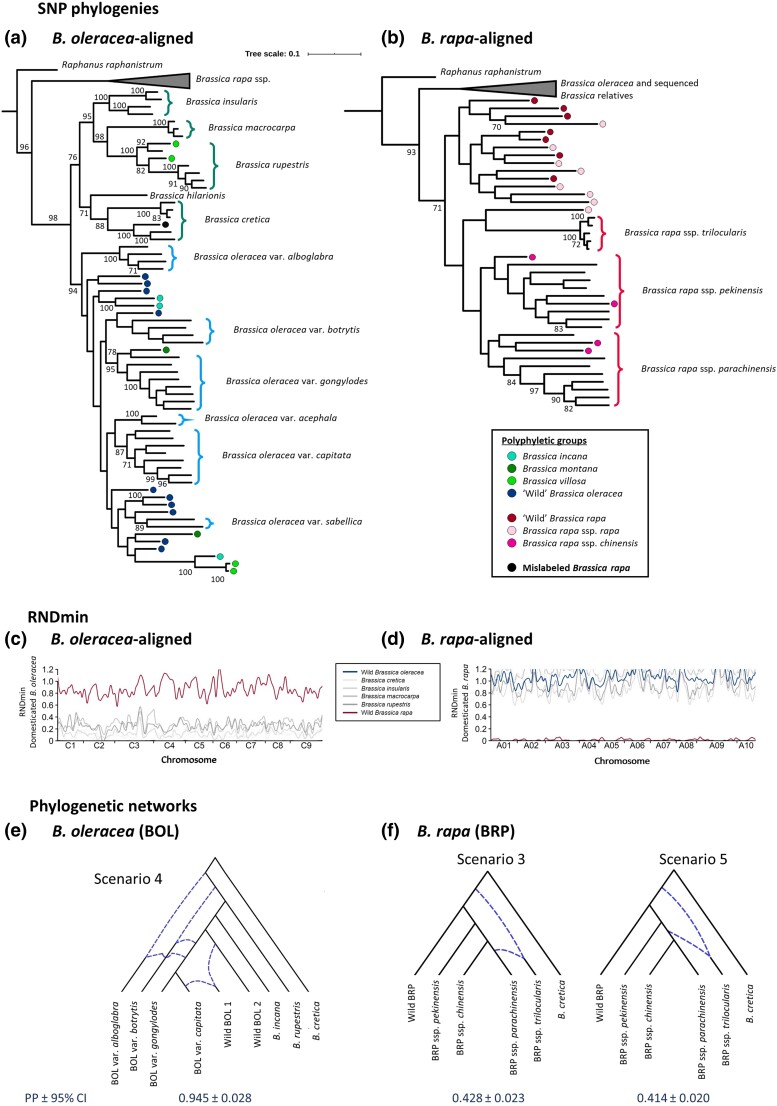
Phylogenetic relationships and hybridization within and between *Brassica oleracea* (a, c, e) and *Brassica rapa* (b, d, f), and their wild relatives. a, b) Maximum likelihood phylogenetic relationships based on single-nucleotide polymorphisms (SNPs) filtered by linkage disequilibrium for samples mapped to a) *B. oleracea* and b) *B. rapa*. Polyphyletic groups are identified by colored dots (see legend), and bootstrap values > 70 are indicated. c, d) RNDmin, a measure of the pairwise distance relative to an outgroup calculated in 50 kb windows for c) domesticated *B. oleracea* versus wild relatives and d) domesticated *B. rapa* (excluding ssp. *rapa*) versus wild relatives. e, f) Most likely phylogenetic networks identified for e) *B. oleracea* (BOL) and f) *B. rapa* (BRP), with dotted lines indicating admixture. Posterior probabilities (PP) with 95% confidence intervals are given below each network.

Phylogenetic trees were constructed from filtered multi-sample gVCFs using maximum likelihood (ML) in SNPhylo (Lee *et al*. 2014); SNPhylo identifies blocks of sequence in LD and keeps one informative SNP per block, which reduces information redundancy while increasing computational tractability. Representative SNPs were extracted with parameters; minimum coverage depth 5 and LD threshold 0.05. SNPs were then concatenated into sequences and aligned using MUSCLE (Edgar 2004) and a phylogenetic tree was determined using DNAML in the PHYLIP package (Felsenstein 1989) with *R. raphanistrum* as the outgroup. Bootstrap analysis was performed using PhyML v3.0 and 100 replications (Guindon *et al*. 2010) and phylogenies were visualized in iTOL (http://itol.embl.de). One wild *B. rapa* individual appeared mislabeled given its position in the phylogenetic tree and was removed from further analysis (black dot, Fig. 1a).

### Relative minimum distance (RMDmin) to wild *Brassica* relatives

This and all subsequent statistical analyses were conducted in R v3.5.2 (R Core Team 2015).

The relative minimum distance between (1) domesticated *B. oleracea* and wild *Brassica* relatives and (2) domesticated *B. rapa* and wild *Brassica* relatives was examined using the summary statistic RNDmin (Rosenzweig *et al*. 2016). RNDmin is a measure of the minimum pairwise distance between populations relative to divergence to an outgroup and was calculated from SNPs in 50 kb windows with a 50 kb step size using R package PopGenome (Pfeifer *et al*. 2014) with outgroup *R. raphanistrum*. RNDmin was plotted using smooth.spline() in R with smoothing parameter 0.4. To determine whether there were significant differences in genome-wide RNDmin averages between comparisons of *B. oleracea* with each of the CWRs and between comparisons of *B. rapa* with each of the four CWRs, a one-way ANOVA was conducted (see Methods S1 for comparisons and further details). *Post hoc* pairwise comparisons were conducted using R package *emmeans* (Lenth *et al*. 2018).

### Genome-wide introgression

Introgression was detected using D-statistics, using Dtrios in Dsuite (Malinsky *et al*. 2020). D-statistics were estimated from biallelic SNPs for trios of populations using *R. raphanistrum* and *E. elatum* as outgroups. A Benjamini–Hochberg multiple test adjustment (Benjamini and Hochberg 1995) was applied (FDR-corrected *P* < 0.05). Genome-wide *fd* (Martin *et al*. 2015) was calculated from windows of 50 informative SNPs across the genome for combinations of taxa. *fd* identifies and estimates the degree of unidirectional introgression from P3 into P2 in four populations with the relationship {[(P1, P2), P3], O}.

### Phylogenetic network analyses

Hybridization in *Brassica* phylogenetic networks was inferred using PhyloNet v3.8.2 (Wen *et al*. 2018) which accounts for incomplete lineage sorting. Since PhyloNet is computationally demanding, multi-sample gVCFs were subsampled to 2–4 representative individuals of wild and domesticated populations of *B. oleracea* and *B. rapa* and one or more monophyletic CWRs (Supplementary Table 1). SNP gVCF files were split into 200 kb regions and converted to PHYLIP files. Suitable nucleotide substitution models were determined using JModeltest2 (Darriba *et al*. 2012). For each genome fragment, phylogenies were constructed using RaxML v8.2.9 (Stamatakis 2014) and bootstrapped with 100 replicates. Resulting trees were converted to nexus files and used to infer phylogenetic networks with zero to five reticulations using the InferNetwork_MPL module. The optimal number of reticulations was determined where the increase in pseudo-likelihood with reticulation number began to plateau (Blair and Ane 2020).

Networks predicted with PhyloNet were evaluated using approximate Bayesian computation (ABC) (Beaumont *et al*. 2002) and used increased sample sizes of four–eight individuals per *B. oleracea* and *B. rapa* population (Supplementary Table 1). Subsets of unlinked SNPs with no missing data were generated and formatted for DIYABC v.2.1.0 (Cornuet *et al*. 2014) using a Python script https://github.com/loire/vcf2DIYABC.py. In DIYABC, uniform distributions were chosen for priors, with 10–10^7^ for the population size and divergence times. All available summary statistics were utilized for *B. rapa*, with a subset of 135 used for the *B. oleracea* analysis (including means of genic diversity and pairwise *F*_ST_) for computational tractability. For each network scenario, 10^6^ simulations were conducted.

The posterior probability of each network was estimated using logistic regression with a logit transformation, based on the number of times that the network appears in the top 1% of simulations when sorted by distance to the observed data set (Cornuet *et al*. 2014). Confidence in network choice was evaluated by calculating type I and type II errors (Cornuet *et al*. 2010).

### Population structure

The population structures within *B. oleracea* and *B. rapa* were analyzed separately. SNPs in LD were filtered out using PLINK v1.07 (Purcell *et al*. 2007) with a 50 kb window size, 5 kb step size, and variant inflation factor 2 and then randomly thinned to 50,000 SNPs. The population structure was analyzed in STRUCTURE v2.3.4 (Pritchard *et al*. 2000) with 1–10 genetic clusters (*K*). Each value of *K* was replicated 10 times, for 20,000 runs following a 10,000 run burn in. Optimal *K* was estimated in STRUCTURE HARVESTER (Earl and VonHoldt 2012) following the *ΔK* method (Evanno *et al*. 2005). Replicates of *K* were aligned, merged, and plotted using R package POPHELPER v2.3.1 (Francis 2017).

### Genome-wide population statistics

Nucleotide diversity, Tajima's *D*, and SNP and indel densities across the *B. oleracea* and *B. rapa* genomes were calculated from filtered SNPs in 50 kb windows using VCFtools v0.1.15 (Danecek *et al*. 2011). Population statistics were plotted using Circos v0.69–6 (Krzywinski *et al*. 2009).

### Demographic history inference

Population size changes over time were inferred for wild and domesticated *B. rapa* and *B. oleracea* using a sequentially Markovian coalescent (SMC) method in SMC++ (Terhorst *et al*. 2017). All domesticated *B. oleracea* varieties excluding *alboglabra* [see results (Fig. 1)] were combined for the *B. oleracea* domesticated population (*n* = 24) and compared with wild *B. oleracea* (*n* = 10). Domesticated *B. rapa* subspecies *trilocularis*, *chinensis*, *parachinensis*, and *pekinensis* were combined for the *B. rapa* domesticated population (*n* = 25), with wild *B. rapa* and *B. rapa ssp. rapa* combined for the wild population (*n* = 15) since the latter are not reciprocally monophyletic (see *Results*). Regions identified as under positive selection (described in the next section) were masked. In the SMC++ sample, frequency spectra are conditioned on a “distinguished lineage” rather than a reference genome. Five to seven “distinguished lineages” were used per population, and each chromosome was analyzed separately. Models were estimated using the *estimate* function, using a mutation rate estimate of 1.5 × 10^−8^ synonymous mutations per generation (Kagale *et al*. 2014) and a generation time of one year as in other analyses (Okazaki *et al*. 2007; McAlvay *et al*. 2021).

### Identification of regions affected by positive selection and targets of selection within them

Recent hard positive selective sweeps were identified by combining outputs from Sweed (Pavlidis *et al*. 2013) and OmegaPlus (Alachiotis *et al*. 2012). Sweed identifies signatures of selection in site frequency spectra using CLR tests, while OmegaPlus looks for signatures of selection in LD using the *ω*-statistic. For analyses of domesticates, domesticated varieties of *B. oleracea* and subspecies of *B. rapa* were analyzed separately (*n* = 4–10). For comparisons of domesticated and wild populations, populations were defined as for demographic history inference.

In Sweed, likelihood ratios are reported for a specific position as well as the genomic window that maximizes CLR for that position. This window is determined dynamically and is biologically relevant since strong selection generally affects large genomic regions (subject to LD decay). In OmegaPlus, statistics are reported for a specific position only. To identify regions affected by positive selection supported in both analyses, first, positions of the top 1% CLR reported in Sweed were retained if the windows that maximized CLR for these positions also contained positions within the top 1% of *ω*-statistics. These positions are referred to as top 1% CLR; *ω*-statistic positions and overlapping associated CLR windows were combined to identify regions affected by positive selection. In an attempt to distinguish between the likely target of positive selection and the genomic window affected by selection, we used custom R scripts to identify regions within windows maximizing CLR for top 1% CLR; *ω*-statistic takes position, starting where the CLR value for the position first crosses the top 1% CLR threshold and ending where it falls below.

Genes overlapping regions targeted by positive selection were extracted using the R package GenomicRanges (Lawrence *et al*. 2013). Gene sequences were compared with The *Arabidopsis* Information Resource (TAIR10) (Berardini *et al*. 2015) using BLASTX (Altschul *et al*. 1990) (*e*-value < 1 × 10^−4^ and >60% sequence identity).

### Gene ontology enrichment analysis

GO enrichment analysis was conducted for genes targetted by selection in each variety/subspecies separately using Fisher's exact test with Benjamini and Yekutieli multiple test adjustment (Benjamini and Yekutieli 2001) (FDR < 0.05), in agriGO v2.0 (Du *et al*. 2010). Venn diagrams of gene ontology (GO) categories in targets of positive selection between domesticates were drawn using eulerr (J. Larsson 2020).

### Parallel selection analysis

Genes identified in target windows of selection were compared with those in a similar analysis using reduction in diversity (ROD) metrics and the population-based integrated haplotype score (PiHS) (Cheng, Sun, et al. 2016). The *B. rapa* genome (Chiifu-401-42) and *B. oleracea* var. *capitata* (line 02–12) v1.0 genome and annotation files were downloaded from BRAD (X. B. Wang *et al*. 2015) and Bolbase (Yu *et al*. 2013), respectively. Genes in regions identified as under selection in Cheng, Sun, *et al*. (2016) were extracted and compared with genes identified in the SFS- and LD-based analyses here using BLASTX (*e*-value < 1 × 10^−4^ and >60% sequence identity).

The genes in candidate target regions were compared for three pairs of *B. oleracea* and *B. rapa* domesticated varieties with similar phenotypes, i.e. early flowering varieties (*B. oleracea* var. *alboglabra* and *B. rapa* ssp. *parachinensis*), heading varieties (*B. oleracea* var. *capitata* and *B. rapa* ssp. *pekinensis*), and enlarged stem varieties (*B. oleracea* var. *gongylodes* and *B. rapa* ssp. *rapa*). Gene fasta files were BLAST searched between pairs to identify putative othologues (BLASTX; *e*-value < 1 × 10^−4^ and >60% sequence identity). Reciprocal best BLAST was used.

Fixed polymorphisms between a domesticated variety and other same species domesticates were identified within 1 kb on either side of genes of interest using vcf-contrast in VCFtools (Danecek et al. 2011), and the sequence was extracted and examined in AliView (A. Larsson 2014).

## Results

Whole genome sequencing (WGS) data of 22 wild *Brassica* individuals (8 wild *B. oleracea*, 8 wild *B. rapa*, and one each of six diploid CWRs; Supplementary Table 1) yielded an average of 43.3 M PE reads (±3.3 M; 95% CI). The six *Brassica* relatives were confirmed to be diploid (610–645 Mbp/1C; Supplementary Table 2). WGS data acquired from further 86 diploid individuals from six publications averaged 27.3 M PE reads (±3.1 M).

### 1. Phylogenomic relationships among *Brassica* species

All 108 samples were mapped to the *B. oleracea* pangenome, and then, to determine the phylogenomic relationships among *B. rapa* and CWRs, relevant samples were mapped to the *B. rapa* genome. The average mapping efficiency rates for these data sets were 72.8 and 75.9%, respectively, and calling and filtering SNPs resulted in 6.0 and 4.1 M SNPs, respectively. Phylogenomic relationships between *Brassica* species (Fig. 1; Supplementary Figs. 1 and 2) demonstrate that *B. rapa* (2*n* = 20) formed a monophyletic clade distinct from *B. oleracea* and all other wild *Brassica* species (2*n* = 18) in both analyses.

In the *B. oleracea*-aligned analysis (Fig. 1a), five of the CWRs (*B. cretica* Lam., *Brassica rupestris* Raf., *Brassica macrocarpa* Guss., *Brassica insularis* Moris, and *Brassica hilarionis* Post) formed a clade, and *B. oleracea* formed another in which wild *B. oleracea* is polyphyletic. *Brassica villosa* Biv. ex Spreng. was found in both the CWR and *B. oleracea* clades; *Brassica incana* Ten. and *Brassica montana* Raf. were found at multiple places in the *B. oleracea* clade. Cultivated varieties of domesticated *B. oleracea* formed monophyletic groups, however often lacked strong bootstrap support.

In the *B. rapa*-aligned analysis (Fig. 1b), wild *B. rapa* and ssp. *rapa* (turnip) formed a clade distinct from the other domesticates and were not reciprocally monophyletic. Three domesticated ssp. (*trilocularis*, *parachinensis*, and *pekinensis*) were monophyletic but with low bootstrap support, and ssp. *chinensis* was polyphyletic.

The summary statistic RNDmin (Rosenzweig *et al*. 2016) was used to calculate the minimum genetic distance between domesticates and CWRs (Fig. 1, c and d). After excluding windows with zero RNDmin, the smallest minimum distance between domesticated *B. oleracea* (combined) and one of the monophyletic CWRs was with *B. cretica* (Fig. 1c; Supplementary Fig. 3a). RNDmin between *B. oleracea* and *B. cretica* was significantly different than the average RNDmin for any of the other three CWRs analyzed (ANOVA; *F*(3, 11,862) = 406.4, *P* < 0.001, Tukey's HSD; all *P* < 0.001; Supplementary Table 3). Although *B. cretica* and other CWRs are equidistant to *B. oleracea* based on the phylogeny, a lower RNDmin for the *B. cretica* comparison could mean that some gene flow between *B. cretica* and *B. oleracea* has caused this apparent similarity. The number of zero RNDmin windows between the wild relatives and domesticated *B. oleracea* (zero RNDmin represents fully conserved or fully introgressed regions) differed (logistic regression; all coefficients *P* < 0.05, Tukey's HSD; all *P* < 0.05; Supplementary Table 3) with *B. cretica* having the most, again suggesting the close relationship, potentially due to introgression, to *B. oleracea*.

RNDmin between domesticated *B. rapa* (excluding ssp. *rapa*) and wild *Brassica* species were much larger than those observed for *B. oleracea* (Fig. 1d), and no zero RNDmin windows were identified, consistent with the relative phylogenetic placement of *B. rapa* and *B. oleracea* (Fig. 1a). The smallest RNDmin was between *B. rapa* and *B. insularis* (Supplementary Figure S3b), smaller than the distance to any other CWR (ANOVA; F(3, 16,742) = 195.5, *P* < 0.001, Tukey's HSD; all *P* < 0.001; Supplementary Table 4).

### 2. Introgression and hybridization among *Brassica* crops and CWRs

#### Among *Brassica oleracea* groups and CWRs

D-statistics detected introgression between all monophyletic CWRs and wild and domesticated *B. oleracea* (Supplementary Table 5, Supplementary Fig. 4). The direction and extent of introgression were further investigated using *fd* (Martin *et al*. 2015). No introgression was detected from CWRs into *B. oleracea* var. *alboglabra* but was found from CWRs into all other domesticated varieties, predicted to account for 0.20–3.61% of the genome (Supplementary Table 6). No introgression from domesticated *B. oleracea* varieties into the CWRs *B. insularis*, *B. macrocarpa*, and *B. rupestris* was detected. However, all domesticated and wild *B. oleracea* varieties exhibited introgression into *B. cretica*, accounting for 9.46–14.28% of the genome.

Phylogenetic networks (allowing one–five reticulations) were constructed from 2174 trees (see *Methods*), for a subset of 22 representative individuals of *B. oleracea* and two monophyletic relatives, *B. cretica* and *B. rupestris* (Supplementary Table 1). There was no clear plateau in pseudo-likelihood with an increasing number of reticulations (Supplementary Fig. 5), highlighting the complexity of relationships. Therefore, the networks with the highest pseudo-likelihood from zero to five reticulations were compared using ABC (Supplementary Table 1 and Supplementary Fig. 6). The network with three reticulations was most likely. Type I and type II error rates for this network were low at 1.1 and 6.2%, respectively, and model checking demonstrated low discordance between simulated network–prior combinations and observed data (Supplementary Table 7). This network (Fig. 1e) included reticulations producing var. *botrytis* (cauliflower), var. *alboglabra* (Chinese kale) and a recently derived wild *B. oleracea* clade (see below). D-statistics between domesticated *B. oleracea* varieties largely support this model showing signals of introgression between *B. oleracea* var. *alboglabra* and the other varieties (*D* = 0.043–0.053, *P* < 0.001) and between *B. oleracea* var. *botrytis* and var. *gongylodes* (*D* = 0.039, *P* < 0.05; Supplementary Table 5).

### Among *Brassica rapa* groups and CWRs

D-statistics also identified introgression between CWRs and *B. rapa* subspecies (Supplementary Table 8, Supplementary Fig. 7). Unidirectional introgression from CWRs into *B. rapa* subspecies was evidenced for the wild *B. rapa*/ssp. *rapa* clade (1.74–1.78% of the genome; Supplementary Table 9), for ssp. *trilocularis* (0.00–1.76%) and to ssp. *pekinensis* (0.03–0.80%). No introgression was detected from *B. rapa* subspecies into *B. cretica* or *B. macrocarpa* but introgression was found into *B. insularis* (0.22–0.88%) and to a lesser extent into *B. rupestris* (Supplementary Table 9). This is consistent with the strongest signal of introgression between *B. insularis* and *B. rapa* subspecies identified by D-statistics and the increased genetic relatedness (RNDmin) compared with other CWRs. Since all subspecies are introgressed with *B. insularis*, this likely represents ancient introgression.

Phylogenetic networks including *B. rapa* subspecies resulted in 1028 trees from 18 representative individuals of *B. rapa* and *B. cretica* (Supplementary Table 1). One reticulation maximized pseudo-likelihood while minimizing the reticulation number (Supplementary Fig. 8) and the five one-reticulation models with the highest pseudolikelihood were compared using ABC (Supplementary Table 1). These support a hybrid origin for ssp. *trilocularis*; two near-identical networks were well-supported (Fig. 1f) but differ slightly in the contributing parental populations (Supplementary Fig. 9). Type II error rates for these networks were low (7.9 and 13.8%) while type I error rates were very high (36.2 and 51.4%), potentially evidencing a lack of SNP variation to distinguish between topologies. Discordance between simulated network–prior combinations and the observed data set was evident (Supplementary Tables 10 and 11); however, it was less for scenario five (Fig. 1e).

### Population structure and domestication history of wild and domesticated *Brassica*s

#### Population structure and demographic history analysis of *B. oleracea* and *B. rapa*

**Fig. 2.**
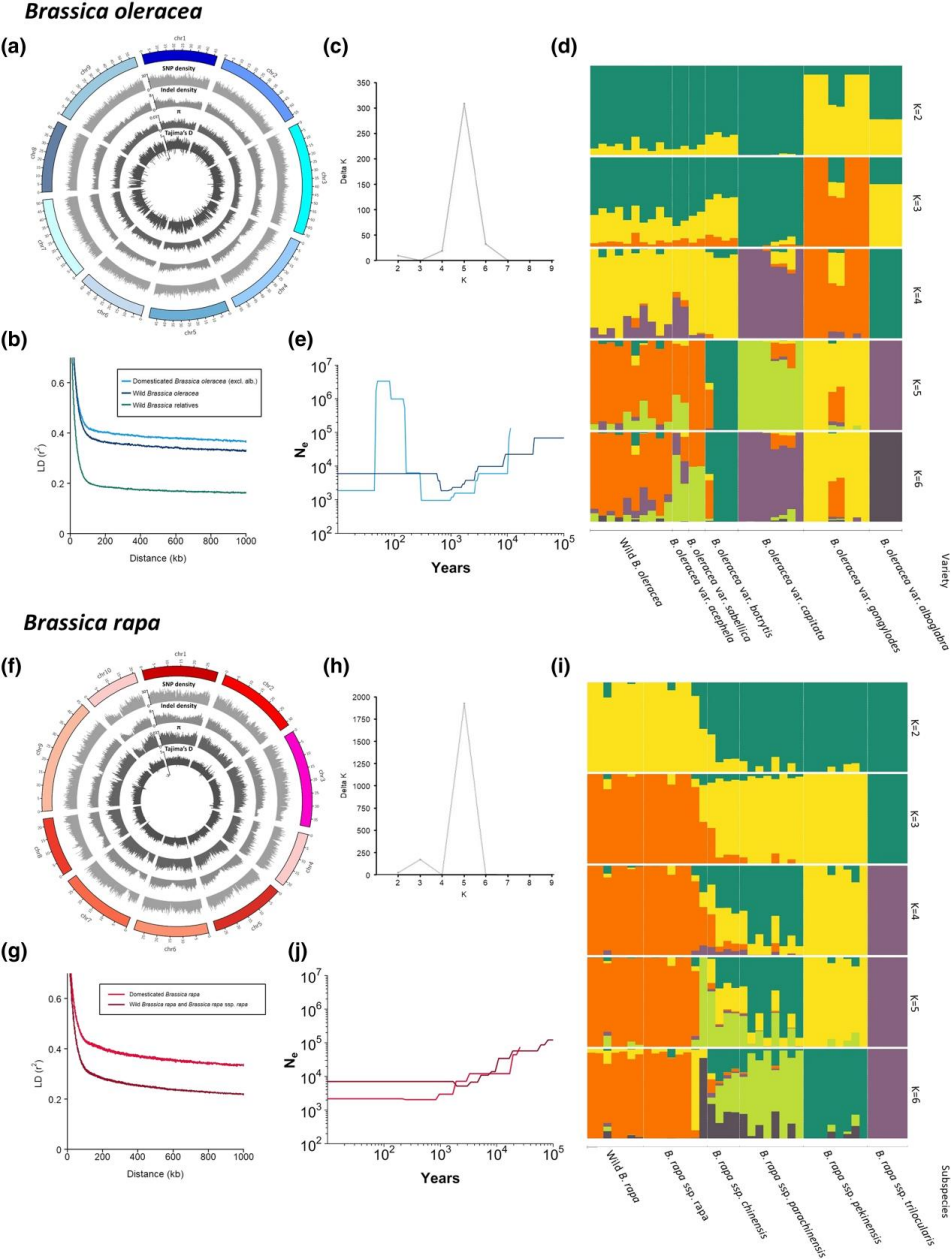
Population genetic statistics and population structure of wild and domesticated *Brassica oleracea* (a–e) and *Brassica rapa* (f–j). a, f) Distribution of population genetic statistics across the genome; b, g) linkage disequilibrium decay; c, h) Evanno's delta *K* for STRUCTURE analyses; d, i) STRUCTURE analysis, with colors representing the proportional assignment of each individual to each of the *K* clusters; e, j) demographic history inference of effective population size over time.

The focused analysis of *B. oleracea* (38 *B. oleracea* samples aligned to the *B. oleracea* pangenome) resulted in 6.1 M SNPs and 1.0 M indels (<50 bp) (Table 1). SNP and indel density, nucleotide diversity [mean 2.6 ± 0.023 × 10^−3^ (95% CI)], and Tajima's *D* [mean 1.738 ± 0.021 (95% CI)] varied throughout the genome (Fig. 2a). The focused analysis of *B. rapa* (41 *B. rapa* samples aligned to the *B. rapa* pangenome) resulted in 5. 8 M SNPs and 0.8 M indels (Table 1). Again, SNP and indel densities, nucleotide diversity [mean 4.5 ± 0.049 × 10^−3^ (95% CI)] and Tajima's *D* [mean 1.780 ± 0.016 (95% CI)] varied throughout the genome (Fig. 2f).

**Table 1.** Summary statistics for *Brassica oleracea* only and *Brassica rapa* only data sets.

Data set	*B. oleracea*-aligned	*B. rapa*-aligned
Reference sequence	*B. oleracea* pangenome	*B. rapa* ssp. *pekinensis* v3.0
Number of individuals	38	41
No. of SNPs post-filtering	8,113,885	5,862,399
No. of indels post-filtering	1,001,661	815,569
Percentage intergenic SNPs	88.6%	85.9%
Percentage exonic SNPs	7.5%	8.9%
Percentage intronic SNPs	3.9%	5.2%
Mean non-synonymous to synonymous ratio	0.781	0.566

LD decay calculated from genome-wide SNPs dropped to half of maximum average *r* ^2^ at c. 53 kb and c. 62 kb for wild and domesticated *B. oleracea*, respectively (Fig. 2b). The difference in LD decay between wild and domesticated *B. rapa* was larger, c. 43 kb and c. 70 kb for the wild *B. rapa*/ssp. *rapa* group and domesticated *B. rapa*, respectively (Fig. 2 g). These are comparable to values for other crops where a recent genetic bottleneck in the domesticated populations has been cited as the cause (X. Huang *et al*. 2012; X. H. Huang *et al*. 2010; Zhou *et al*. 2015).

### Domestication history of *B. oleracea*

There was low bootstrap support for the relative positions of wild and domesticated *B. oleracea* populations in phylogenetic analyses; however, network analyses support var. *alboglabra* as the earliest diverging lineage (Fig. 1). This supports the ABC analyses (above) that wild *B. oleracea* accessions are feral derivatives, not wild ancestors (Fig. 1, a and c). In STRUCTURE analysis of *B. oleracea*, the number of underlying populations was estimated as five (Fig. 2, c and d). Varieties *alboglabra*, *gongylodes*, *capitata*, and *botrytis* largely formed distinct genetic clusters with a fifth cluster including wild *B. oleracea*, var. *sabellica* and var. *acephela*. Wild *B. oleracea* individuals show admixture from each of the domesticated clusters.

Domesticated *B. oleracea* (excluding var. *alboglabra*) experienced a decline in effective population size from 10 Kya to c. 300 years ago (Ne = 133,000 to Ne = 1000), followed by a prominent expansion c. 40 years ago (Ne = 3,333,000) and rapid decline to the present day (Fig. 2e). This is consistent with a long history of cultivation, global distribution of cultivated varieties, and then improvement of *B. oleracea* varieties. The effective population size of wild *B. oleracea* similarly declined, from 100 Kya (Ne = 123,000) to 500 ya (Ne = 2000), which could represent shared ancestry during the cultivation of *B. oleracea* until 1 Kya, supporting STRUCTURE analysis.

### Domestication history of *B. rapa*

STRUCTURE analysis of *B. rapa* identified five clusters, three were clearly delimited (wild *B. rapa*/ssp. *rapa*, ssp. *trilocularis* and ssp. *pekinensis*), and ssp. *chinensis* was partially assigned to a fourth and ssp. *parachinensis* to a fifth, but with extensive admixture (Fig. 2). This largely matches the phylogenetic clades identified above. Domesticated *B. rapa* subspecies (excluding ssp. *rapa*) experienced a decline in effective population size from 25 Kya to c. 1 Kya (Ne = 75,000 to Ne = 2000) and the wild *B. rapa*/ssp. *rapa* population declined from 200 Kya (Ne = 137,000) to 2 Kya (Ne = 5000), followed by a small increase (Fig. 2j). Considering the above analyses and the extant geographical ranges, this could describe complex parallel and largely independent cultivation histories, i.e. early cultivation of ssp. *rapa* in Europe with later independent domestication in South-East Asia (ssp. *chinensis*, *pekinensis*, and *parachinensis*) and with the divergence of ssp. *trilocularis* in Southern Asia (Qi *et al*. 2017). The lack of significant recent expansion in *B. rapa*, compared with the prominent expansion *B. oleracea*, could reflect greater recent introgression from the wild in the latter.

### Positive selection during domestication

#### Shared selection in wild and domesticated populations of *B. oleracea* and *B. rapa*

**Fig. 3.**
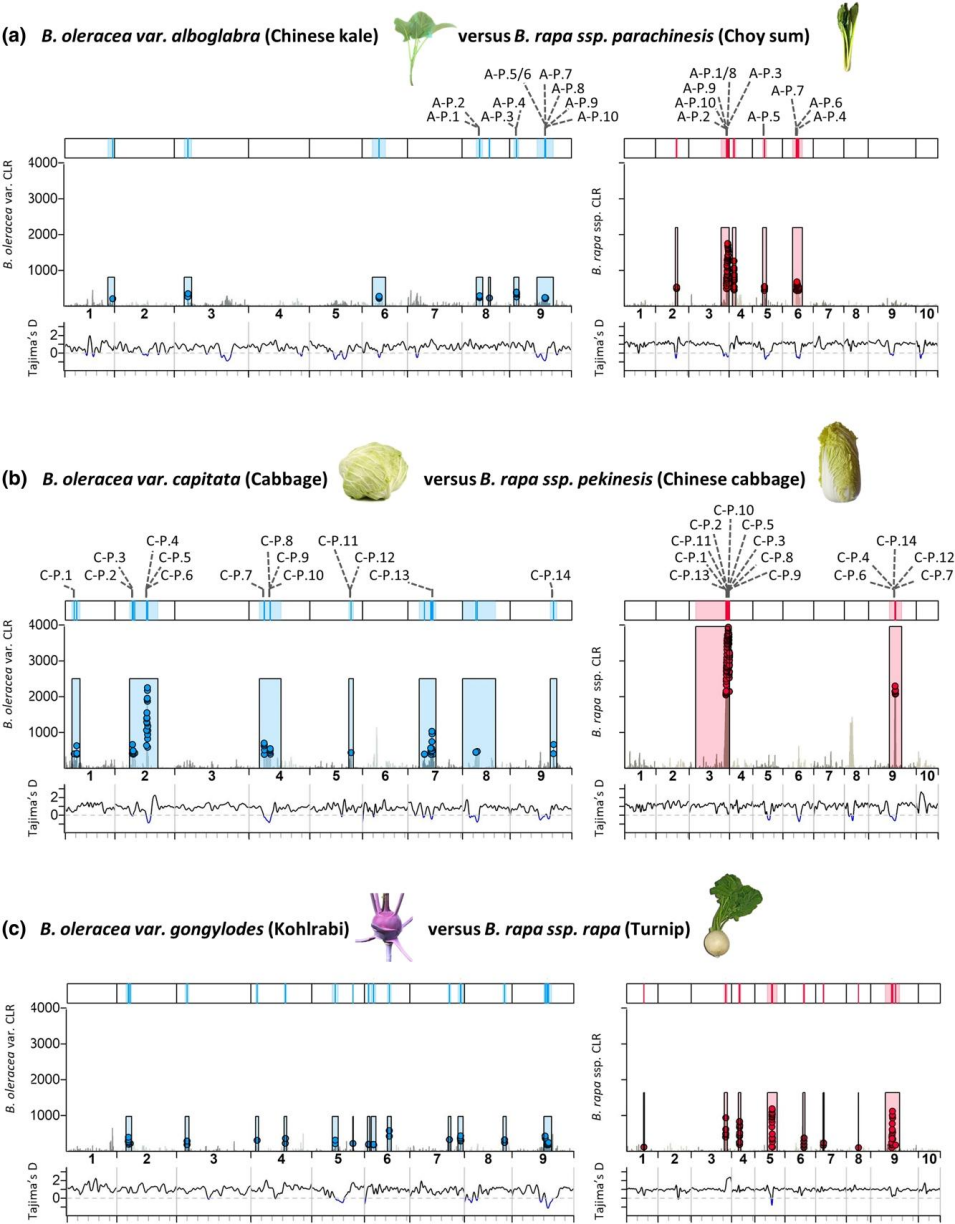
Signatures of selection in *Brassica oleracea* and *Brassica rapa*. a, b) Overlap between genomic regions targeted by positive selection in a) wild (bottom) and domesticated (top) *B. oleracea* (excluding *B. oleracea var. alboglabra*) and b) overlap between regions targeted by positive selection in combined wild (bottom, including *B. rapa* ssp. *rapa*) and domesticated (top) *B. rapa*. CLR values in the top 1% of both the CLR (Sweed) and *ω*-statistic (OmegaPlus) are highlighted as red or blue points. Shaded boxes define windows around these points that maximize CLR. Bars at the top show the location of these windows affected by selection (light) and the likely targets of selection within them (dark). Overlapping regions are indicated with arrows and numbers indicate the number of genes in the overlap and the number with AT annotations. c) Size (Mb) of genomic regions targeted by positive selection and their overlap between domesticated *B. oleracea* varieties and between *B. rapa* subspecies. d) Overlap in gene ontology categories that were enriched in regions targeted by positive selection.

Genomic regions targeted by positive selection were identified through composite likelihood ratio (CLR) tests of site frequency spectra (SFS) in Sweed (Pavlidis *et al*. 2013) and LD patterns (*ω*) using OmegaPlus (Alachiotis *et al*. 2012) (see *Methods* for details). Several regions targeted by positive selection were identified in wild and domesticated (excluding var. *alboglabra*; see above) *B. oleracea*, with regions on chromosomes 4 and 5 overlapping (Fig. 3a). In these overlapping regions, there were 38 genes (Supplementary Table 12) but only 14 had an *Arabidopsis* BLAST hit and no biological processes were significantly enriched in GO analysis of these. Several regions of selection were identified in wild and domesticated *B. rapa*; however, no regions overlapped for wild *B. rapa*/ssp. *rapa* and domesticated subspecies (Fig. 3b).

### Between domesticates within species

Combined analyses of SFS and LD identified regions targeted by recent positive selection in all domesticates (Fig. 3c). For *B. oleracea*, each domesticate showed overlap in regions of positive selection with at least one other domesticate, but for *B. rapa*, the only overlap was limited to the three subspecies with large leaf phenotypes (Cheng, Wu, *et al*. 2016), overlapping geographic ranges and a shared domestication history (Fig. 3c). The other two domesticates [ssp. *trilocularis* (oilseed) and ssp. *rapa* (turnip)] showed no overlap.

On average, regions targeted by positive selection represented 0.76 ± 0.24% (3.65 MB) of the assembled chromosomes for *B. oleracea* domesticates and 1.29 ± 0.67% (2.49 MB) for *B. rapa* domesticates, containing an average of 355 and 230 annotated genes, respectively. The smallest proportion was for ssp. *trilocularis* with only 0.06% of the genome (0.14 MB; 18 genes).

Despite the close relationship between *Arabidopsis* and *Brassica*, *Arabidopsis* orthologues were identified for only 53% of genes (Supplementary Tables 13-25), which may have limited detection of potentially key genes. Gene ontology analysis evidenced large overlap of functions and processes in the genes in these regions across domesticates, with few group-specific enriched GO categories (Fig. 3d, Supplementary Tables 26-28). GO categories such as “multicellular organism development” and “response to stimulus” were enriched (FDR < 0.05, Fisher's exact test) for all domesticated and wild populations, except *B. rapa* ssp. *trilocularis*, again highlighting the distinctiveness of this subspecies.

### Parallel selection during domestication for similar phenotypes

To analyze parallel selection for similar phenotypes, genes in positive-selection target regions were compared for (1) *B. oleracea* var. *alboglabra* and *B. rapa* ssp. *parachinensis* (early flowering/leafy varieties), (2) *B. oleracea* var. *capitata* and *B. rapa* ssp. *pekinensis* (heading varieties), and (3) *B. oleracea* var. *gongylodes* and *B. rapa* ssp. *rapa* (enlarged stem varieties).

For comparisons (2) and (3), similar comparisons have been carried out previously (Cheng, Sun, *et al*. 2016) using earlier genome assemblies and alternative methods for identifying positive selection. Because of the introgression and admixture that we resolved, we used a single-population approach to identify regions under selection rather than comparisons between domesticates or putatively wild groups used previously. For each of the four groups in these two comparisons, we compared genes in candidate regions targeted by selection in our analysis with those identified as under selection in Cheng, Sun, *et al*. (2016). 40% of the genes identified in target regions in *B. rapa* ssp. *pekinensis*, 25% in *B. oleracea* var. *capitata*, 44% in *B. rapa* ssp. *rapa*, and 31% in *B. oleracea* var. *gongylodes* were also identified in Cheng, Sun, *et al*. (2016) and this is significantly more than expected by chance (*χ* ^2^ test; *χ* ^2^ = 21.4–59.5, all *P* < 0.01), suggesting that the approaches show broad agreement.

**Fig. 4.**
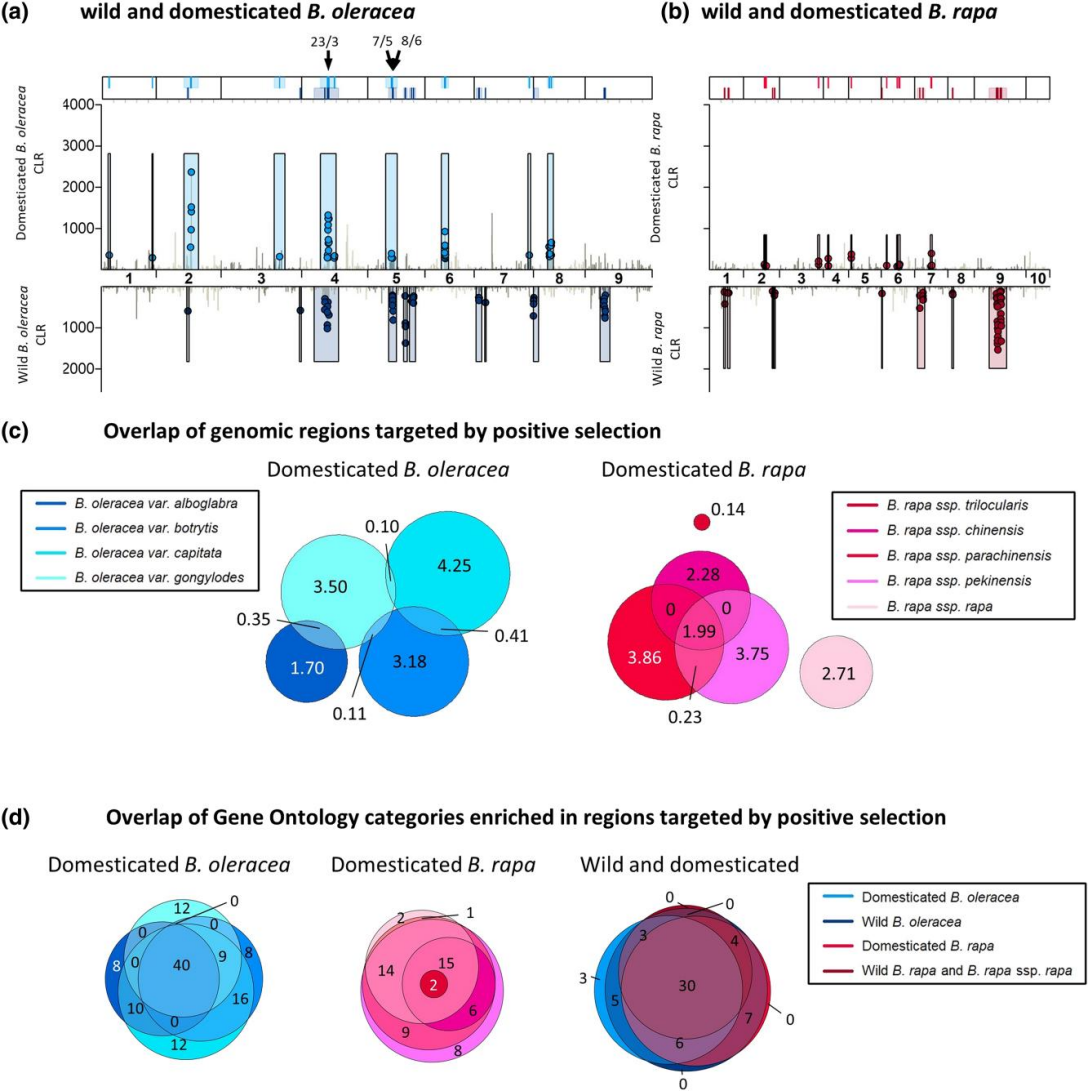
Evidence for parallel positive selection between pairs of *Brassica oleracea* domesticates (left) and *Brassica rapa* domesticates (right) with similar phenotypes. CLR values in the top 1% CLR values and top 1% of *ω*-statistic values highlighted as blue and red points for *B. oleracea* and *B. rapa*, respectively. Shaded boxes define windows around these points that maximize CLR. The top bars show the location of these windows affected by selection (light) and the candidate target regions of selection within them (dark). Positions of putative *rapa*-*oleracea* orthologues in target selection regions according to reciprocal BLAST are indicated (full gene information is given in SI Appendix, Supplementary Table 11). Tajima's *D* is plotted below with negative values highlighted in blue.

We then looked at shared selection between groups of the two species that share the same phenotype. For comparisons (1) and (2), 10 and 14 putative *B. rapa*–*B*. *oleracea* orthologues were identified in selection target regions (Fig. 4); more than expected by chance (*χ* ^2^ test; *χ* ^2^ = 7.2, *P* < 0.01, and *χ* ^2^ = 11.1, *P* < 0.001, respectively). In contrast, no putative orthologues were found in comparison 3 (large stem varieties). Among parallel pairs of genes, only 27% of the 24 gene pairs received an *Arabidopsis* hit, but we still detected over-representation of GO terms involved in transport, methylation, and transcription (Supplementary Table 29).

### Selection on genes involved in anatomical structure development

GO annotation of genes in candidate selection target regions highlights promising candidates for follow-up. Of these, genes annotated with the GO term “anatomical structure development” are briefly discussed.

(1) Early flowering and leafy varieties: *B. oleracea* var. *alboglabra* and *B. rapa* ssp. *parachinensis*

Two of six such genes in regions targeted by positive selection in *B. oleracea* var. *alboglabra* and one of the 10 in *B. rapa* ssp. *parachinensis* were involved in auxin response. Both gene sets also contained putative orthologues of genes involved in floral development; *CULLIN3* is encoding a positive regulator of floral development (Chahtane *et al*. 2018) in var. *alboglabra* and *ICMB* encoding a negative regulator of signaling pathways affecting floral development (Bracha-Drori *et al*. 2008) in ssp. *parachinensis*.

(2) Heading varieties: *B. oleracea* var. *capitata* and *B. rapa* ssp. *pekinensis*

Regions targeted by positive selection on chromosome 3 of ssp. *pekinensis* contained three genes with this GO annotation, including *ALE1*, encoding a subtilisin protease associated with leaf development (Tanaka *et al*. 2001). Irregularities in the coordination of leaf polarity are thought to play a key role in the formation of the heading phenotype in ssp*. pekinensis* (Li *et al*. 2019) and a putative orthologue of a gene involved in this pathway, *KANADI-2* (Yamaguchi *et al*. 2012), was identified on chromosome 9 of ssp. *pekinensis*.

In var. *capitata*, a putative orthologue of *ASL5*, which operates in the same adaxial–abaxial polarity pathway as KANADI-2, was one of seventeen anatomical structure genes in regions targeted by selection. This pathway therefore warrants further investigation for the heading phenotype in both species.

(3) Enlarged stem varieties: *B. oleracea* var. *gongylodes* and *B. rapa* ssp. *rapa*

In enlarged stem varieties, there was no clear overlap in the pathways of genes targeted by selection, although there were potential candidates. In var. *gongylodes*, *WVD2* was one of twelve anatomical structure development genes. Overexpression of *WVD2* in *Arabidopsis* results in shorter, stockier roots, and stems (Perrin *et al*. 2007). In ssp. *rapa*, an orthologue of a gene encoding a transcription factor that regulates organ size when overexpressed in *Arabidopsis* (*ANT*) is in a region targeted by positive selection (Ding *et al*. 2018).

### Causative SNPs in genes of interest

Among the genes of interest (i.e. under parallel selection or annotated as “anatomical structure development” genes; Supplementary Tables 30-31), the only fixed difference between a domesticated variety and other varieties occurred in *AAT* on chromosome 8 for *B. oleracea* var. *gongylodes* (kohlrabi). This gene functions in the biosynthesis of aromatic amino acids that have diverse roles as precursors to secondary metabolites such as anthocyanins (Tzin and Galili 2010). Three fixed SNPs result in amino acid replacement and therefore potentially functional changes.

## Discussion

This analysis further demonstrates the complex phylogenomic relationships between *Brassica* crops and their CWRs, quantifying for the first time the extent of introgression in their diversification and domestication. We then adopt a single-population analysis strategy to identify candidate genomic regions under selection during domestication. The incorporation of adaptive genetic diversity from CWRs into crops is a key strategy to improve crop resilience to climate change to ensure future food security (Castañeda-Álvarez *et al*. 2016). The resolution of *Brassica* CWR and crop genetic relationships therefore has direct application to these diverse and economically important crops.

### Phylogenomic relationships among *Brassica* species and the potential for *Brassica* crop improvement

Two recent phylogenies constructed to model the *B. rapa* and *B. oleracea* groups (including a small number of CWRs) within the core *oleracea* lineage used genotyping-by-sequencing (McAlvay *et al*. 2021) and RNA-seq (Mabry *et al*. 2021). We present largely concordant findings, but with additional insight using alternative outgroups and increased resolution afforded by WGS.

Mabry *et al*. (2021) suggest that *B. cretica* is the closest CWR to *B. oleracea*, whereas we argue that *B. cretica* is better described as a member of a cluster of CWRs distinct from *B. oleracea* (also found by Song *et al*. 1990 using RFLPs). The Mabry *et al*. (2021) analysis uses a sample of *B. villosa* as an outgroup, which both our study and their study indicate is not monophyletic. Our study instead used *Raphanus* as an outgroup which helped us to highlight the relationships between the CWRs. We further identify gene flow between *B. oleracea* and *B. cretica*, which could explain the close phylogenetic position resolved by Mabry *et al*. (2021). This gene flow likely took place prior to domestication, given that all domesticated groups show introgression (accounting for 9.46–14.28% of the genome) with *B. cretica*.

The close relationships between several CWRs highlight that all these CWRs could be considered potential sources of adaptive genetic variation for *B. oleracea* breeding. Indeed, introgression was detected from several CWRs into crop varieties of both *B. oleracea* and *B. rapa* demonstrating that crossing is likely to be successful. It is important to note that of these CWRs, three are near threatened or critically endangered (Bilz *et al*. 2011) and are poorly represented in seed banks (Castañeda-Álvarez *et al*. 2016), highlighting an urgent need to collect and preserve their genetic diversity.

Our data also confirm that wild *B. oleracea* populations are not monophyletic and are not the ancestors of all *B. oleracea* crops, supporting conclusions that wild populations along the Atlantic coast are feral derivatives (Maggioni *et al*. 2018; Mittell *et al*. 2020; Mabry *et al*. 2021). We also show that these populations possess significant admixture from domesticated varieties. Regardless, the combination of closely related CWRs that can hybridize with domesticated *B. oleracea* (FitzJohn *et al*. 2007) and the pool of potentially adaptive novel allele combinations in admixed wild populations provide an extensive resource for breeding *B. oleracea* crops where adaptive genetic diversity is lacking (Katche *et al*. 2019).

In agreement with McAlvay *et al*. (2021), in our analyses, wild populations of *B. rapa* are polyphyletic with *B. rapa* ssp. *rapa* (turnip), which raises several possibilities about the taxonomic status of ssp. *rapa*. The turnip phenotype could be a plastic response to a cultivated environment (and hence not a genetically fixed phenotype) and has evolved multiple times, and/or some “wild” *B. rapa* populations are feral ssp. *rapa*. McAlvay *et al*. (2021) asserting that true wilds are present in the Caucasus and Italy, a group that we did not identify, but our sampling of wild *B. rapa* was less geographically extensive. Feral *B. rapa* populations could provide potential for intraspecific breeding of *B. rapa* domesticates. To our knowledge, experimental crosses between the CWRs and *B. rapa* have not been conducted, but our evidence for introgression suggests that this is possible.

Overall, the finding of polyphyly of *B. villosa*, *B. montana*, and *B. incana* (see also Mabry *et al*. 2021; McAlvay *et al*. 2021) highlights that any putatively wild *B. rapa* and other CWRs should be re-examined alongside samples from other *Brassica* species.

### The role of introgression and hybridization among *Brassica* crops and CWRs in domestication and diversification

We detected hybrid origins of domesticates in both *B. oleracea* and *B. rapa*. In *B. oleracea*, both var. *alboglabra* (Chinese kale) and var. *botrytis* (cauliflower) were identified as having hybrid origins between unknown wild *Brassica* species and a more recently derived *B. oleracea* var. *gongylodes* and var. *capitata* lineage. A previous GBS SNP analysis also suggested that var. *botrytis* is derived from introgression, albeit with var. *italica* (Stansell *et al*. 2018) which we did not sample. The greatest proportion of introgressed genomic sites was detected from *B. cretica* (Stansell *et al*. 2018) into var. *botrytis* but phylogenetic network analysis highlights an unidentified wild species as the parental species. Var. *alboglabra* contrasts with all other domesticates, in that no introgression was detected from the four monophyletic CWRs and could suggest that this group was domesticated outside the range of CWRs.

Based on the WGS phylogeny, var. *alboglabra* forms a unique population that diverges before other *B. oleracea* varieties, which is also shown in other analyses (Izzah *et al*. 2013; Cheng, Sun, *et al*. 2016; Stansell *et al*. 2018). *B. oleracea* is generally considered to have been domesticated in the Mediterranean (Arias *et al*. 2014; Maggioni *et al*. 2018; Mabry *et al*. 2021) where the core oleracea lineage originated c. 3 Mya (Arias *et al*. 2014). However, phylogenetic placement of var. *alboglabra* might suggest an earlier independent domestication. From an initial hybrid origin, presumably in the Mediterranean, a subsequent absence of introgression from the CWRs indicates that var. *alboglabra* was geographically isolated from wild relatives during its domestication. Since var. *alboglabra* is widely cultivated in China and South-East Asia (Dixon, 2006), the hybrid lineage could have been transported to Asia where subsequent selection and domestication took place. In this way, hybridization may have provided a starting point for the cultivation of var. *alboglabra* while an absence of introgression with wild relatives promoted domestication.

Qi *et al*. (2017) evidence a stepwise eastward progression of *B. rapa* domestication over 2000–4000 years, with turnip and Chinese cabbage cultivation corroborated by written records, and McAlvay *et al*. (2021) suggest that introgression may have been prominent in a subset of these Central Asian oilseed crops, which our data support. Network analysis suggested a hybrid origin of ssp*. trilocularis* deriving from the ssp. *parachinensis*/ssp. *chinensis* lineage and an unknown, potentially wild, lineage (Fig. 1f). Previous analyses support that ssp. *trilocularis* forms part of a genetically distinct Asian population of rapid cycling domesticates selected for high-seed oil content; however, these did not include CWRs (Cheng, Wu, *et al*. 2016; Bird *et al*. 2017). The potential hybrid origin of ssp. *trilocularis* should be followed up after more wild taxa are investigated.

Previously, Qi *et al*. (2017) identified *B. rapa* ssp. *pekinensis* as a hybrid between ssp. *rapa* and ssp. *chinensis* which is partly supported by McAlvay *et al*. (2021). Although our analysis does not support this, the reduced sampling of *B. rapa* ssp. *chinensis* in our analysis, or the absence of *B. cretica* sampling by Qi *et al*. (2017), could have led to this discrepancy.

### Positive selection and parallel evolution during domestication

The considerable phenotypic variation in domesticated *Brassicas* provides opportunities to investigate parallel evolution, similarly explored in other crops (Lin *et al*. 2012; Wang *et al*. 2018). Hybrid origins of some domesticated varieties, introgression between domesticates and CWRs, and the emergence of domesticated groups in geographical isolation from CWRs highlight the phylogenetic complexity of this group. Consequently, our analysis employs single-population approaches to identify targets of selection during domestication rather than traditional comparative approaches (Cheng, Sun, *et al*. 2016). This could be advantageous in other systems too, where phylogenetic analysis has identified complex histories of hybridization between domesticated varieties and with wild relatives (Flowers *et al*. 2019; Page *et al*. 2019). Although using smaller sample sizes compared with previous analyses (Cheng, Sun, *et al*. 2016), our analysis does make use of updated genome assemblies.

Our analysis of selection in domesticated groups, and parallel evolution among crops selected for similar phenotypes, identified further potential targets with importance for breeding programs. These may also be relevant to research in similar phenotypes for other crop species. For one such gene (*AAT*), kohlrabi exhibited fixed non-synonymous SNPs compared with other domesticates. This gene functions in the production of aromatic amino acids, and variants have been associated with flowering time and yield in lentil (Skibinski *et al*. 1984). Furthermore, aromatic amino acids are precursors to anthocyanins (Winkel-Shirley, 2001) which produce the purple color of some kohlrabi varieties (Park *et al*. 2017; Petropoulos *et al*. 2019). Consumption of these anthocyanins can have health benefits (Kim *et al*. 2017); thus, this gene warrants further study with reference to human health and exemplifies the potential application of this positive selection analysis. Other genes worthy of further investigation include ALE1 and ASL5 both putatively involved in leaf development and identified in the selection analysis of heading varieties of both crops.

We also note that different numbers of genomic loci appear to show signatures of selection during the evolution of different domesticated groups. For example, only 0.06% of the genome (0.14 MB; 18 genes) showed evidence for selection in *B. rapa* ssp. *trilocularis*, which may suggest that the evolution of yellow seeds and high seed oil content characteristic of this taxon involved few genes.

## Conclusions

Our study demonstrates through a range of approaches and genome sequencing of CWRs that hybridization and introgression have been instrumental in the evolution of *Brassica* crops as well as continuing more recently between crops and wild relatives. Our selection analysis, which should be less prone to interference from past hybridization, identified targets of selection during *Brassica* domestication. Overall, we show that there are several CWRs with potential to hybridize with domesticated Brassica species and we identify candidate genes for adaptive phenotypes worthy of follow-up.

## Supplementary Material

iyad027_Supplementary_Data

## Data Availability

All raw sequencing data generated in this study have been deposited in the NCBI SRA under project number PRJNA929712. Supplemental material available at GENETICS online.
