# Supplementary material for: Extensive crop–wild hybridization during Brassica evolution and selection during the domestication and diversification of Brassica crops
Source: Genetics. 2023 Feb 22;223(4):iyad027. doi: 10.1093/genetics/iyad027 (PMC10078912; doi:10.1093/genetics/iyad027)
Supplement: iyad027_Supplementary_Data [file iyad027_supplementary_data.zip › Methods_S1,_Tables_S2-S11,_Table_S29_GENETICS-2022-305730.docx]

Supporting Information

**Article title: Extensive crop-wild hybridisation during Brassica evolution, and selection during the domestication and diversification of Brassica crops**

**Authors:** Jasmine M. Saban, Anne J. Romero, Thomas H. G. Ezard & Mark A. Chapman

**Article acceptance date:**

The following Supporting Information is available for this article:

**Methods S1** Extended materials and methods.

**Figure S1** Maximum likelihood phylogeny of *Brassica* species based on single nucleotide polymorphisms (filtered by linkage disequilibrium) identified by mapping resequencing data of 108 samples to the *Brassica* *oleracea* pangenome.

**Figure S2** Maximum likelihood phylogeny of *Brassica* species based on single nucleotide polymorphisms (filtered by linkage disequilibrium) identified by mapping resequencing data of 77 samples to the *Brassica* *rapa* ssp. *pekinensis* genome.

**Figure S3** Average genome-wide relative minimum distance (RNDmin) between domesticated *Brassica* crops and wild monophyletic *Brassica* species relative to outgroup *Raphanus raphanistrum*.

**Figure S4** Signals of introgression between *Brassica oleracea* varieties and wild *Brassica* relatives as a heatmap of significant (P<0.05, FDR correction) D-statistics.

**Figure S5** Pseudolikelihood of models inferred for zero to five reticulations in phylogenetic network analysis of *Brassica oleracea.*

**Figure S6** Phylogenetic networks identified as having the highest pseudolikelihood for number of reticulations 5:0 (a-f) in analysis of *Brassica oleracea.*

**Figure S7** Signals of introgression between *Brassica rapa* varieties and wild *Brassica* relatives as a heatmap of significant (P<0.05, FDR correction) D-statistics.

**Figure S8** Pseudolikelihood of models inferred for zero to five reticulations in phylogenetic network analysis of *Brassica rapa.*

**Figure S9** The five phylogenetic networks with highest pseudolikelihood for one reticulation in analysis of *Brassica rapa* phylogenies.

**Figure S10** Density distribution of annotation values informing SNP discovery for 108 individual samples aligned to the *Brassica oleracea* pangenome assembly.

**Figure S11** Density distribution of annotation values informing INDEL discovery for 108 individual samples aligned to the *Brassica oleracea* pangenome assembly.

**Figure S12** Density distribution of annotation values informing SNP discovery for 77 individual samples aligned to the *Brassica rapa* v3.0 genome assembly.

**Figure S13** Density distribution of annotation values informing INDEL discovery for 77 individual samples aligned to the *Brassica rapa* v3.0 genome assembly.

**Table S1 (separate file)** Meta-data for samples used in whole genome sequencing analysis. Accession information is provided along with an outline of the samples used in each analysis.

Supporting_Information_Table_S1.xlsx

**Table S2** Genome size of wild *Brassica* relatives determined using flow cytometry.

**Table S3** Statistical analysis of differences in relative minimum distances between domesticated *B. oleracea* varieties and wild *Brassica* relatives.

**Table S4** Statistical analysis of differences in relative minimum distances between domesticated *B. oleracea* varieties and wild *Brassica* relatives.

**Table S5** D-statistics used to test for signals of introgression between *Brassica oleracea* varieties and wild *Brassica* relatives.

**Table S6** Estimated genome-wide proportion of introgressed sites using the *fd* statistic in *Brassica oleracea* analyses.

**Table S7** ABC model checking for Scenario 4 in network analysis of *Brassica oleracea* phylogenies.

**Table S8** D-statistics used to test for signals of introgression between *Brassica rapa* varieties and wild *Brassica* relatives.

**Table S9** Estimated genome-wide proportion of introgressed sites using the *fd* statistic in *Brassica rapa* analyses.

**Table S10** ABC model checking for Scenario 3 in network analysis of one reticulation *Brassica rapa* phylogenies.

**Table S11** ABC model checking for Scenario 5 in network analysis of one reticulation *Brassica rapa* phylogenies.

**Table S12 (separate file)** Genes identified as overlapping with peaks of positive selection in both domesticated *B. oleracea* (excluding var. *alboglabra*) and Wild *B. oleracea*.

**Table S13-S25 (separate file)** Genes identified as overlapping with peaks of positive selection, and their associated AT identifier where applicable for all analyses: Wild *B. oleracea*, *B. oleracea* var*. alboglabra*, *B. oleracea* var*. capitata*, *B. oleracea* var*. botrytis*, *B. oleracea* var*. gongylodes*, combined domesticated *B. oleracea* (excl. alboglabra), Wild *B. rapa*, *B. rapa* ssp. *chinensis, B. rapa* ssp. *parachinensis, B. rapa* ssp. *pekinensis, B. rapa* ssp. *trilocularis, B. rapa* ssp. *rapa,* combined domesticated *B. rapa* (excluding ssp. rapa).

Supporting_Information_Tables_S12toS24.xlsx

**Table S26-S28 (separate file)** GO categories identified as enriched for genes in positive selection peaks for comparison of: Wild and domesticated *B. oleracea* and *B. rapa*, domesticated *B. oleracea* varieties, and domesticated *B. rapa* subspecies. All enriched GO categories identified in the analysis are listed with the presence or absence of this term in the enrichment of each population indicated with 1 or 0.

Supporting_Information_Tables_S26toS28.xlsx

**Table S29** Descriptions for putative *B. oleracea* – *B. rapa* orthologues (identified as reciprocal best blast pairs) in peaks of positive selection of domesticates with similar phenotypes.

**Table S30-S31 (separate file)** Genes of interest identified as putative orthologues under parallel selection (reciprocal best blast) or annotated as “anatomical structure development” genes for *B. oleracea* and *B. rapa.*

Supporting_Information_Tables_S30toS31.xlsx

**Methods S1** Extended materials and methods.

**Genome size estimation**

Leaf samples from six wild Brassica relative species were harvested and sent on wet ice to Plant Cytometry Services (<http://www.plantcytometry.nl/>). DNA content (2C) was established using flow cytometry with comparison to the standard *Pachysandra terminalis* Siebold and Zucc. (3.5 pg/2C).

**Whole genome resequencing**

Seeds were obtained for 22 wild *Brassica* accessions: eight wild *Brassica oleracea* accessions, eight wild *B. rapa* accessions and six crop wild relatives (CWRs). These were obtained from Warwick UK Vegetable Genebank (<https://warwick.ac.uk/fac/sci/lifesci/wcc/gru/genebank/seed/>), the U.S. National Plant Germplasm System (<https://npgsweb.ars-grin.gov/gringlobal/search>), and the Leibniz Institute of Plant Genetics and Crop Plant Research Genebank (<https://www.ipk-gatersleben.de/en/genebank/>). Seeds were grown in the glasshouse at the University of Southampton and DNA was extracted from frozen leaf material using a modified CTAB protocol (Doyle & Doyle, 1990). Novogene Bioinformatics Institute (Cambridge, UK) performed library preparation of the samples and carried out 150 bp paired-end sequencing (with 350 base insert size) using an Illumina 2500 platform (Illumina, USA).

**Acquisition of additional resequencing data**

Additional resequencing reads were obtained for 84 samples from previously published datasets. Whole genome resequencing data from four wild *Brassica cretica* isolates (Kioukis *et al.*, 2020) were downloaded and subsampled to 30 M reads to ensure consistency with the other datasets. Low coverage genome skimming data for 21 samples were obtained for diverse *B. rapa*, *B. oleracea* and wild *Brassica* relatives (An *et al.*, 2019) to add additional breadth to the sampling. Genome resequencing data for 59 domesticated *B. rapa* and *B. oleracea* samples were obtained from Cheng *et al.* (2016b). Two sets of paired-end reads obtained from *Erucastrum elatum* O.E. Schulz (Kiefer *et al.*, 2019) , were concatenated to provide sufficient coverage. *Raphanus raphanistrum* raw reads were downloaded as a subset of those used to assemble the radish genome (Moghe *et al.*, 2014). Where analyses only allowed for one outgroup, *R. raphanistrum* was used and *E. elatum* was additionally as an outgroup where possible. Accession information for all 108 sequencing samples can be found in Supporting Information Table S1.

**Processing sequencing data**

Raw WGS data and the data obtained from previous publications (above), was quality checked using FastQC (Andrews, 2010). Sequences were trimmed and filtered with Trimmomatic v0.36 (Bolger *et al.*, 2014), which removed Illumina adapters, the first 5 bases of the sequence, leading and trailing N bases with quality below 5, and where the average quality per base of a sliding window dropped below 15. Reads shorter than 40 bp were removed. Data obtained from An *et al.* (2019) and Kiefer *et al.* (2019) were already trimmed. After quality control steps, samples had an average of 11.3x coverage ± 1.4 [95% CI] based on a 600 Mb genome (Supporting Information Table S2).

**Alignment and SNP filtering**

Preliminary and subsequent analysis indicated that wild *Brassica* relatives mapped more efficiently to the *Brassica oleracea* pangenome than the *Brassica rapa ssp. pekinensis* v3.0 genome (Zhang *et al.*, 2018) (Supporting Information Table S1). Therefore all 108 acquired samples were aligned to the *Brassica oleracea* pangenome (Golicz *et al.*, 2016) using Bowtie2 v2.3.1 (Langmead & Salzberg, 2012) for phylogenetic analysis. To analyse the relationship between *B. rapa* subspecies and related *Brassica* species, a subset of 77 samples were aligned to the *B. rapa* ssp. *pekinensis* genome. Alignment files were processed to obtain high quality SNPs as described below. For all subsequent analyses only alignments to whole chromosomes were considered.

Bam files were processed with Picard v2.8.3 (picard.sourceforge.net), unmapped and non-unique reads were filtered out, and the bam files were sorted, duplicated reads were removed and read groups were added. Reads were then realigned around indels using RealignerTarget creator and IndelRealigner in the Genome Analysis Toolkit v3.7 (GATK) (Van der Auwera *et al.*, 2013). Variant detection was performed using the GATK pipeline, with variants called using HaplotypeCaller to create single sample genomic Variant Call Format files (gVCF), with minimum phred-scaled confidence threshold of 30. gVCFs were combined into a multisample gVCF using the CombineGVCFs module of GATK. For genotyping, the multisample gVCF was split into regions for tractability, samples were genotyped using GenotypeGVCFs and then recombined. Indel and SNP variants were called separately using SelectVariants in GATK. High quality SNPs and indels were extracted using the Variant Filtration module of GATK.

To filter SNPs produced by alignment to the *B. oleracea* pangenome the following SNP filtering expression was used: QUAL < 0 || MQ < 20 || SOR > 2 || QD < 2 || FS > 60|| MQRankSum < -12 || ReadPosRankSum < -8. To filter SNPs produced by alignment to the *B. rapa ssp. pekinensis* genome, the SNP filtering expression was; MQ < 25 || SOR > 3 || QD < 2 || FS > 60 || MQRankSum < -12 || ReadPosRankSum < - 8. High quality indels were extracted using the indel filtering expression as SOR > 3 || QD < 10 || FS > 60 || ReadPosRankSum < - 8 || Inbreeding coefficient <-0.8. These parameters were determined following examination of their distribution in the raw SNP and indel datasets (Supporting Information Figures S10-S13). Indels were further filtered to include sites where there was identity information for <50% of the dataset, to remove indels with minor allele frequency <0.05, and to remove indels >50 bp. Linkage disequilibrium (LD) decay was calculated using PopLDdecay v3.40 (Zhang *et al.*, 2019).

**SNP annotation**

SNPs in the two datasets were annotated according to the annotation files available for the *Brassica oleracea* pangenome and the *Brassica rapa* ssp. *pekinensis* genome. SNP annotation was performed using SNPeff v5.0 (Cingolani *et al.*, 2012).

**SNP phylogenies**

Phylogenetic trees were constructed from filtered multisample gVCFs using maximum likelihood in SNPhylo (Lee *et al.*, 2014). SNPhylo identifies blocks of sequence in LD and keeps one informative SNP per block, which reduces information redundancy while increasing computational tractability. Phylogenies were constructed using SNPs with a minimum depth of coverage of 5, LD threshold of 0.05 and using *R. raphanistrum* as an outgroup. Bootstrap analysis was performed using PhyML v3.0 (Guindon *et al.*, 2010) and phylogenies were visualised in iTOL (<http://itol.embl.de>). In phylogenetic reconstruction, one wild *B. rapa* individual appeared to be mislabelled and was removed from further analysis (black dot, Figure 1a).

**Relative minimum distance to wild *Brassica* relatives**

Relative minimum distance between (1) domesticated *B. oleracea* and wild *Brassica* relatives, and (2) domesticated *B. rapa* and wild *Brassica* relatives were examined using the summary statistic RNDmin (Rosenzweig *et al.*, 2016). RNDmin is a measure of the minimum pairwise distance between populations relative to the divergence to an outgroup. RNDmin was calculated from filtered SNP calls in 50 kb windows with a 50 kb step size using R package PopGenome (Pfeifer *et al.*, 2014) and using *R. raphanistrum* as the outgroup. RNDmin was plotted with smoothing by fitting a cubic smoothing spline using the smoothing.spline() function with smoothing parameter 0.4 in R base packages. To determine whether there were significant differences in genome-wide RNDmin averages, RNDmin in non-zero windows were log-transformed, and a one-way ANOVA was conducted. Zero RNDmin values affected comparisons between *B. oleracea* and CWRs only, zeros were removed to conduct a one-way ANOVA of non-conserved regions. Additionally, the differences in the number of fully introgressed or fully conserved RNDmin windows (RNDmin=0) were analysed using logistic regression. *Post hoc* pairwise comparisons were conducted using Tukey's honestly significant difference (HSD) test in R package *emmeans* (Lenth *et al.*, 2018) for logistic regression analysis. All statistical analyses were conducted in R v3.5.2 (RCoreTeam, 2015).

**Genome-wide introgression**

D-statistics were utilised to test for signals of introgression between taxa and were calculated using Dtrios in Dsuite (Malinsky *et al.*, 2020). D-statistics were estimated from multisample gVCF files filtered for biallelic SNPs, for combinations of trios of populations and using *R. raphanistrum* and *E. elatum* as outgroups. A Benjamini-Hochberg multiple test adjustment (Benjamini & Hochberg, 1995) was applied with maximum FDR corrected p‑value <0.05. To explore the direction and extent of introgression in these comparisons the genome-wide *fd* statistic was calculated from windows across the genome (50 informative SNPs) for combinations of taxa. The *fd* statistic (Martin *et al.*, 2015) estimates unidirectional introgression from P3 to P2 in four populations with the relationship (((P1,P2),P3),O). A positive *fd* evidences introgression and estimates the proportion of introgressed sites across the genome.

**Phylogenetic network analysis using PhyloNet**

PhyloNet v3.8.2 (Wen *et al.*, 2018) was used to infer hybridisation and introgression in phylogenetic networks (accounting for incomplete lineage sorting) in the *Brassica* datasets. Since PhyloNet is computationally demanding, multisample gvcfs were subset to include two or more representative individuals of wild and domesticated populations of *B. oleracea* and *B. rapa*, and one or more monophyletic wild relatives (22 individuals for *B. oleracea* analysis and 18 individuals for the *B. rapa* dataset; Supporting Information Table S1). SNP gVCF files were split into 200 kb regions across the genomes and converted to PHYLIP files. JModeltest2 (Darriba *et al.*, 2012), conducted on 10 randomly selected fragments, was used to identify suitable nucleotide substitution models for analyses. For each genome fragment in each of the two analyses, phylogenies were constructed using RaxML v8.2.9 (Stamatakis, 2014) with maximum likelihood and a GTRCAT substitution model, bootstrapped with 100 replicates. This resulted in 2174 trees for the *B. oleracea* dataset and 1028 trees for the *B. rapa* dataset. Trees were converted to nexus files and used to infer phylogenetic networks with zero, one, two, three, four and five reticulations using the InferNetwork_MPL option of PhyloNet. Pseudolikelihood of the top five outputted models for each number of reticulations was plotted against the number of reticulations, to determine the number of reticulations at which the increase in pseudolikelihood with reticulation number began to plateau, as recommended (Blair & Ane, 2020).

**Evaluating phylogenetic networks with ABC**

To assess the extent to which hybridisation may have contributed to the domestication of *B. rapa* and *B. oleracea*, evolutionary networks predicted with PhyloNet were evaluated using Approximate Bayesian Computation (ABC)(Beaumont *et al.*, 2002). These network scenarios were tested using increased sample sizes of individuals selected based on phylogenies; 43 individuals for *B. oleracea* analysis and 41 individuals for the *B. rapa* analysis (see Supporting Information Table S1 for samples used). SNPs were filtered by LD using the SNPRelate package (Zheng *et al.*, 2012) in R, generating a subset of unlinked SNPs with no missing data. VCF files were converted to the correct input format for the software DIYABC v.2.1.0 (Cornuet *et al.*, 2014) using a python script available from <https://github.com/loire/vcf2DIYABC.py>. For implementing the analyses in DIYABC, a uniform distribution was chosen for each prior, with wide intervals of 10-10^7^ for populations sizes and 10-10^7^ for divergence times. All available summary statistics were utilised for *B. rapa*, while a subset of 135 summary statistics were used for the *B. oleracea* analysis for computational tractability. The subset of summary statistics included means of genic diversities and pairwise F_ST_. For each network scenario in each analysis, 10^6^ simulations were conducted.

The posterior probability of each network was estimated using logistic regression with a logit transformation, based on the number of times the network appears in the top 1% of simulations when these are sorted by distance to the observed dataset (Cornuet *et al.*, 2014). Confidence in network choice was evaluated by calculating Type I and Type II error according to Cornuet *et al.* (2010). Five hundred test datasets (pseudo-observed) were simulated for each analysed network, drawing parameters from the prior distribution. The posterior probability of each network was calculated using logistic regression. Type I error was calculated as the proportion of datasets simulated under the specified (“best”) network where the highest posterior probability was assigned to an alternate network. Type II error was calculated as the proportion of datasets simulated under alternate networks that had highest posterior probability assigned to the specified network. The model checking function of DIYABC was used to assess the goodness-of-fit of datasets generated under specified network-parameter posterior combinations to the observed data. One thousand datasets were simulated for each specified network-posterior combination. All utilised summary statistics were applied, and this can result in overfitting since the same statistics are used for inference and model checking (Cornuet *et al.*, 2010).

**Population structure**

Population structure within *B. oleracea* and *B. rapa* were analysed separately. SNPs were pruned to those in approximate LD using PLINK v1.07 (Purcell *et al.*, 2007) with 50 kb window size, 5 kb step-size, and variant inflation factor 2, then randomly thinned to 50,000 SNPs. Population structure was analysed in STRUCTURE v2.3.4 (Pritchard *et al.*, 2000) with genetic cluster numbers predefined for K=1:10. Each value of K was replicated 10 times, for 20,000 runs following a 10,000 run burn in. Optimal K was estimated in STRUCTURE HARVESTER (Earl & VonHoldt, 2012) following the ΔK method described by Evanno *et al.* (2005). Replicates of K were aligned, merged and plotted using R package POPHELPER v2.3.1 (Francis, 2017).

**Genome-wide population statistics**

To examine the distribution of SNP and indel densities across the *B. oleracea* and *B. rapa* genomes, densities were calculated from filtered SNPs in 50 kb windows using VCFtools v0.1.15 (Danecek *et al.*, 2011). VCFtools were also used to calculate nucleotide diversity and Tajima’s D. Distribution of whole genome population statistics were plotted using Circos v0.69-6 (Krzywinski *et al.*, 2009). For Figure 4, Tajima’s D is plotted with smoothing by fitting a cubic smoothing spline using the *smoothing.spline*() function with smoothing parameter 0.6 for each chromosome in R base packages.

**Demographic history inference**

Population size changes over time were inferred for wild and domesticated *B. rapa* and *B. oleracea* using a sequentially Markovian coalescent (SMC) method, implemented in SMC++ (Terhorst *et al.*, 2017). The domesticated *B. oleracea* varieties *capitata, gongylodes*, *botrytis, acephala* and *sabellica* (excluding *alboglabra*, which may represent an older divergence; see results) were combined for the *B. oleracea* domesticated population (n=24) and compared to wild *B. oleracea* (n=10). For the *B. rapa* analysis, the domesticated population included ssp. *trilocularis, chinensis, parachinensis* and *pekinensis* (n=25) while the wild population consisted of the combined wild *B. rapa* and *B. rapa ssp. rapa* individuals (n=15) due to these populations not being reciprocally monophyletic (see results). Regions identified as under positive selection (described below) were masked. Five to seven “distinguished lineages” (*sensu* SMC++) were used and each chromosome was analysed separately. Models were estimated using the *estimate* function, using a mutation rate estimate of 1.5 x 10^-8^ synonymous mutations per generation based on recent estimations for *B. rapa* (3.95 x 10^-9^ - 1.42 x 10^-8^; Park et al 2019) and the Brassicaceae (Kagale *et al.*, 2014). Generation time was assumed to be one year.

**Identification of regions affected by positive selection and targets of selection within them**

Recent hard positive selective sweeps were identified by combining outputs from Sweed (Pavlidis *et al.*, 2013) and Omegaplus (Alachiotis *et al.*, 2012). Sweed identifies signatures of selection in site frequency spectra using CLR tests, while Omegaplus looks for signatures of selection in LD patterns using the ω-statistic. For analyses of domesticates, domesticated varieties of *B. oleracea* and subspecies of *B. rapa* were analysed separately (n=4-10). For comparisons of domesticated and wild populations, domesticated *B. oleracea* consisted of all *B. oleracea* varieties except *alboglabra* (n=24) and domesticated *B. rapa* included ssp. *trilocularis, chinensis, parachinensis* and *pekinensis* (n=25). Wild *B. rapa* consisted of the wild *B. rapa* accessions combined with *B. rapa* ssp*. rapa* (n=15).

In Sweed, likelihood ratios are reported for a specific position, but the window that maximises CLR for that position is also reported and usually overlaps positions with lower likelihood ratios. This window is determined dynamically and is biologically relevant since strong selection generally affects large windows of the genome (subject to LD decay). In Omegaplus, statistics are reported for a specific position only. To combine these outputs to identify regions affected by positive selection supported in both analyses, positions of the top 1% CLR reported in Sweed were retained if the windows that maximised CLR for these positions also contained positions with the top 1% of ω-statistic values. These positions are referred to as top 1% CLR;ω-statistic positions and associated windows were combined where they overlap to identify regions affected by positive selection.

Since windows maximising CLR for top 1% CLR;ω-statistic can contain CLR positions with likelihood ratios not in the top 1% of values, custom R scripts were used to further identify candidate target regions of positive selection within these windows as follows. For each CLR position across the chromosome, if the CLR value for the position is below the threshold for the top 1% CLR but the next position is a top 1% CLR;ω-statistic position, then this position is the start of the target region. If the position is a top 1% CLR;ω-statistic position, and the next position has a CLR value below the top 1% CLR threshold, then the next position forms the end of the target region. Where these occur at the beginning or end of a chromosome, the beginning or end of the chromosome forms the start or end of the target region respectively. This approach attempts to distinguish between the likely target of positive selection and the genomic window affected by selection.

Regions targeted by positive selection were identified for each population and genes overlapping these regions were extracted using the R package GenomicRanges (Lawrence *et al.*, 2013). Gene sequences were compared against The *Arabidopsis* Information Resource (TAIR10)(Berardini *et al.*, 2015) using BLASTX (Altschul *et al.*, 1990) (e-value <1x10^-4^ and >60% sequence identity). Gene descriptions for AT numbers were obtained using TAIR (<https://www.arabidopsis.org/tools/bulk/genes/index.jsp>).

**Gene ontology (GO) enrichment analysis**

GO enrichment analysis was conducted for sets of genes targetted by selection for each variety or subspecies separately using a Fisher’s exact test with Benjamini and Yekutieli multiple test adjustment (Benjamini & Yekutieli, 2001) (FDR <0.05), implemented using agriGO v2.0 (Du *et al.*, 2010). Overlaps in enriched gene ontology categories were identified using base packages and Venn diagrams were drawn using the eulerr package (Larsson, 2020).

**Parallel selection analysis**

**Comparison of genes in positive selection target regions in Cheng *et al.* (2016a)**

Genes identified in windows of selection in this analysis, and in a similar analysis Cheng *et al.* (2016a), which used different metrics were compared. The *B. rapa* reference genome (Chiifu-401-42) was downloaded from BRAD (Wang *et al.*, 2015) and *B. oleracea* var. *capitata* (line 02–12) v1.0 genome was downloaded from Bolbase (Yu *et al.*, 2013), along with their respective annotation files. Genes in regions identified as under selection by either reduction in diversity (ROD) metrics or population-based integrated haplotype score (PiHS) in Cheng *et al.* (2016a) were extracted. These were compared to genes identified in the SFS and LD based analyses presented here, using BLASTX (e-value <1x10^-4^ and >60% sequence identity).

**Putative orthologues**

The genes in selection target regions were compared for three pairs of *B. oleracea* and *B. rapa* domesticated varieties with similar phenotypes (see main text). Fasta files of genes in regions were BLAST searched between pairs to identify pairs of putatively othologous genes (BLASTX; e-value <1x10^-4^ and >60% sequence identity). Reciprocal best BLAST was used to ensure true orthologues were identified.

**Syntenic orthologues**

Syntenic orthologues between *B. oleracea* and *B. rapa* were identified by comparing peptide fasta sequences and synteny in the genome annotation files provided with genome assemblies, using the software SynOrths (Cheng *et al.*, 2012). Parameters specified 20 genes flanking each side of the query gene were considered with 100 genes flanking the reference genes and using 0.2 as the minimum ratio of flanking genes that were a BLASTP best hit.

**Identification of causative polymorphisms in genes of interest**

Polymorphisms between a single domesticated variety and other domesticated varieties of the same species were subset using vcf-contrast in VCFtools (Danecek *et al.*, 2011). These were filtered to only include polymorphisms within 1 kb either side of genes of interest (parallel selection genes or those with anatomical structured development annotation). Only one gene of interest contained fixed polymorphisms, the sequence for this gene was extracted using vcf-consensus and examined in AliView (Larsson, 2014).

## **References**

**Alachiotis N, Stamatakis A, Pavlidis P. 2012.** OmegaPlus: a scalable tool for rapid detection of selective sweeps in whole-genome datasets. *Bioinformatics* **28**(17): 2274-2275.

**Altschul SF, Gish W, Miller W, Myers EW, Lipman DJ. 1990.** Basic Local Alignment Search Tool. *Journal of Molecular Biology* **215**(3): 403-410.

**An H, Qi XS, Gaynor ML, Hao Y, Gebken SC, Mabry ME, McAlvay AC, Teakle GR, Conant GC, Barker MS, et al. 2019.** Transcriptome and organellar sequencing highlights the complex origin and diversification of allotetraploid Brassica napus. *Nature Communications* **10**.

**Andrews S 2010**. FastQC: a quality control tool for high throughput sequence data. <http://www.bioinformatics.babraham.ac.uk/projects/fastqc>.

**Beaumont MA, Zhang WY, Balding DJ. 2002.** Approximate Bayesian computation in population genetics. *Genetics* **162**(4): 2025-2035.

**Benjamini Y, Hochberg Y. 1995.** Controlling the false discovery rate - a practical and powerful approach to multiple testing. *Journal of the Royal Statistical Society Series B-Statistical Methodology* **57**(1): 289-300.

**Benjamini Y, Yekutieli D. 2001.** The control of the false discovery rate in multiple testing under dependency. *Annals of Statistics* **29**(4): 1165-1188.

**Berardini TZ, Reiser L, Li DH, Mezheritsky Y, Muller R, Strait E, Huala E. 2015.** The *Arabidopsis* Information Resource: Making and mining the "gold standard" annotated reference plant genome. *Genesis* **53**(8): 474-485.

**Blair C, Ane C. 2020.** Phylogenetic Trees and Networks Can Serve as Powerful and Complementary Approaches for Analysis of Genomic Data. *Systematic Biology* **69**(3): 593-601.

**Bolger A, Lohse M, Usadel B. 2014.** Trimmomatic: a flexible trimmer for Illumina sequence data. *Bioinformatics* **30**: 2114-2120.

**Cheng F, Sun RF, Hou XL, Zheng HK, Zhang FL, Zhang YY, Liu B, Liang JL, Zhuang M, Liu YX, et al. 2016a.** Subgenome parallel selection is associated with morphotype diversification and convergent crop domestication in *Brassica rapa* and *Brassica oleracea*. *Nature Genetics* **48**(10): 1218-1224.

**Cheng F, Wu J, Cai CC, Fu LX, Liang JL, Borm T, Zhuang M, Zhang YY, Zhang FL, Bonnema G, et al. 2016b.** Genome resequencing and comparative variome analysis in a *Brassica rapa* and *Brassica oleracea* collection. *Scientific Data* **3**.

**Cheng F, Wu J, Fang L, Wang XW. 2012.** Syntenic gene analysis between *Brassica rapa* and other Brassicaceae species. *Frontiers in Plant Science* **3**.

**Cingolani P, Platts A, Wang LL, Coon M, Nguyen T, Wang L, Land SJ, Lu XY, Ruden DM. 2012.** A program for annotating and predicting the effects of single nucleotide polymorphisms, SnpEff: SNPs in the genome of *Drosophila melanogaster* strain w(1118); iso-2; iso-3. *Fly* **6**(2): 80-92.

**Cornuet J-M, Pudlo P, Veyssier J, Dehne-Garcia A, Gautier M, Leblois R, Marin J-M, Estoup A. 2014.** DIYABC v2.0: a software to make approximate Bayesian computation inferences about population history using single nucleotide polymorphism, DNA sequence and microsatellite data. *Bioinformatics* **30**: 1187-1189.

**Cornuet J-M, Ravigné V, Estoup A. 2010.** Inference on population history and model checking using DNA sequence and microsatellite data with the software DIYABC (v1.0). *BMC Bioinformatics* **11**(1): 401.

**Danecek P, Auton A, Abecasis G, Albers CA, Banks E, DePristo MA, Handsaker RE, Lunter G, Marth GT, Sherry ST, et al. 2011.** The variant call format and VCFtools. *Bioinformatics* **27**(15): 2156-2158.

**Darriba D, Taboada GL, Doallo R, Posada D. 2012.** jModelTest 2: more models, new heuristics and parallel computing. *Nature Methods* **9**(8): 772-772.

**Doyle JJ, Doyle JL. 1990.** Isolation of plant DNA from fresh tissue. *Focus* **12**: 13-15.

**Du Z, Zhou X, Ling Y, Zhang Z, Su Z. 2010.** agriGO: a GO analysis toolkit for the agricultural community. *Nucleic Acids Research* **38**: W64–W70.

**Earl DA, VonHoldt BM. 2012.** STRUCTURE HARVESTER: a website and program for visualizing STRUCTURE output and implementing the Evanno method. *Conservation genetics resources* **4**(2): 359-361.

**Evanno G, Regnaut S, Goudet J. 2005.** Detecting the number of clusters of individuals using the software STRUCTURE: a simulation study. *Molecular Ecology* **14**(8): 2611-2620.

**Francis RM. 2017.** pophelper: an R package and web app to analyse and visualize population structure. *Molecular Ecology Resources* **17**(1): 27-32.

**Golicz AA, Bayer PE, Barker GC, Edger PP, Kim H, Martinez PA, Chan CKK, Severn-Ellis A, McCombie WR, Parkin IAP, et al. 2016.** The pangenome of an agronomically important crop plant *Brassica oleracea*. *Nature Communications* **7**.

**Guindon S, Dufayard JF, Lefort V, Anisimova M, Hordijk W, Gascuel O. 2010.** New Algorithms and Methods to Estimate Maximum-Likelihood Phylogenies: Assessing the Performance of PhyML 3.0. *Systematic Biology* **59**(3): 307-321.

**Kagale S, Robinson SJ, Nixon J, Xiao R, Huebert T, Condie J, Kessler D, Clarke WE, Edger PP, Links MG, et al. 2014.** Polyploid Evolution of the Brassicaceae during the Cenozoic Era. *Plant Cell* **26**(7): 2777-2791.

**Kiefer C, Willing EM, Jiao WB, Sun HQ, Piednoel M, Humann U, Hartwig B, Koch MA, Schneeberger K. 2019.** Interspecies association mapping links reduced CG to TG substitution rates to the loss of gene-body methylation. *Nature Plants* **5**(8): 846-855.

**Kioukis A, Michalopoulou VA, Briers L, Pirintsos S, Studholme DJ, Pavlidis P, Sarris PF. 2020.** Intraspecific diversification of the crop wild relative *Brassica cretica* Lam. using demographic model selection. *Bmc Genomics* **21**(1).

**Krzywinski M, Schein J, Birol I, Connors J, Gascoyne R, Horsman D, Jones SJ, Marra MA. 2009.** Circos: An information aesthetic for comparative genomics. *Genome Research* **19**(9): 1639-1645.

**Langmead B, Salzberg SL. 2012.** Fast gapped-read alignment with Bowtie 2. *Nature Methods* **9**(4): 357-359.

**Larsson A. 2014.** AliView: a fast and lightweight alignment viewer and editor for large datasets. *Bioinformatics* **30**(22): 3276-3278.

**Larsson J 2020**. eulerr: Area-Proportional Euler and Venn Diagrams with Ellipses. <https://cran.r-project.org/package=eulerr>.

**Lawrence M, Huber W, Pages H, Aboyoun P, Carlson M, Gentleman R, Morgan MT, Carey VJ. 2013.** Software for Computing and Annotating Genomic Ranges. *Plos Computational Biology* **9**(8).

**Lee TH, Guo H, Wang XY, Kim C, Paterson AH. 2014.** SNPhylo: a pipeline to construct a phylogenetic tree from huge SNP data. *Bmc Genomics* **15**.

**Lenth R, Singmann H, Love J, Buerkner P, Herve M 2018**. Emmeans: Estimated marginal means, aka least-squares means. <https://CRAN.R-project.org/package=emmeans>.

**Malinsky M, Matschiner M, Svardal H. 2020.** Dsuite - Fast D-statistics and related admixture evidence from VCF files. *Molecular Ecology Resources*.

**Martin SH, Davey JW, Jiggins CD. 2015.** Evaluating the use of ABBA–BABA statistics to locate introgressed loci. *Molecular Biology and Evolution* **32**(1): 244-257.

**Moghe GD, Hufnagel DE, Tang HB, Xiao YL, Dworkin I, Town CD, Conner JK, Shiu SH. 2014.** Consequences of Whole-Genome Triplication as Revealed by Comparative Genomic Analyses of the Wild Radish *Raphanus raphanistrum* and Three Other Brassicaceae Species. *Plant Cell* **26**(5): 1925-1937.

**Pavlidis P, Zivkovic D, Stamatakis A, Alachiotis N. 2013.** SweeD: Likelihood-Based Detection of Selective Sweeps in Thousands of Genomes. *Molecular Biology and Evolution* **30**(9): 2224-2234.

**Pfeifer B, Wittelsburger U, Ramos-Onsins SE, Lercher MJ. 2014.** PopGenome: An Efficient Swiss Army Knife for Population Genomic Analyses in R. *Molecular Biology and Evolution* **31**(7): 1929-1936.

**Pritchard JK, Stephens M, Donnelly P. 2000.** Inference of population structure using multilocus genotype data. *Genetics* **155**(2): 945-959.

**Purcell S, Neale B, Todd-Brown K, Thomas L, Ferreira MA, Bender D, Maller J, Sklar P, De Bakker PI, Daly MJ. 2007.** PLINK: a tool set for whole-genome association and population-based linkage analyses. *The American journal of human genetics* **81**(3): 559-575.

**RCoreTeam. 2015.** R: A Language and Environment for Statistical Computing. Vienna, Austria: R Foundation for Statistical Computing. [*https://www.R-project.org/*](https://www.R-project.org/).

**Rosenzweig BK, Pease JB, Besansky NJ, Hahn MW. 2016.** Powerful methods for detecting introgressed regions from population genomic data. *Molecular Ecology* **25**(11): 2387-2397.

**Stamatakis A. 2014.** RAxML version 8: a tool for phylogenetic analysis and post-analysis of large phylogenies. *Bioinformatics* **30**(9): 1312-1313.

**Terhorst J, Kamm JA, Song YS. 2017.** Robust and scalable inference of population history froth hundreds of unphased whole genomes. *Nature Genetics* **49**(2): 303-309.

**Van der Auwera GA, Carneiro MO, Hartl C, Poplin R, Del Angel G, Levy-Moonshine A, Jordan T, Shakir K, Roazen D, Thibault J, et al. 2013.** From FastQ data to high confidence variant calls: the Genome Analysis Toolkit best practices pipeline. *Curr Protoc Bioinformatics* **43**(1110): 11.10.11-11.10.33.

**Wang XB, Wu J, Liang JL, Cheng F, Wang XW. 2015.** *Brassica* database (BRAD) version 2.0: integrating and mining Brassicaceae species genomic resources. *Database-the Journal of Biological Databases and Curation*.

**Wen DQ, Yu Y, Zhu JF, Nakhleh L. 2018.** Inferring Phylogenetic Networks Using PhyloNet. *Systematic Biology* **67**(4): 735-740.

**Yu JY, Zhao MX, Wang XW, Tong CB, Huang SM, Tehrim S, Liu YM, Hua W, Liu SY. 2013.** Bolbase: a comprehensive genomics database for *Brassica oleracea*. *Bmc Genomics* **14**.

**Zhang C, Dong SS, Xu JY, He WM, Yang TL. 2019.** PopLDdecay: a fast and effective tool for linkage disequilibrium decay analysis based on variant call format files. *Bioinformatics* **35**(10): 1786-1788.

**Zhang L, Cai X, Wu J, Liu M, Grob S, Cheng F, Liang JL, Cai CC, Liu ZY, Liu B, et al. 2018.** Improved *Brassica rapa* reference genome by single-molecule sequencing and chromosome conformation capture technologies. *Horticulture Research* **5**.

**Zheng XW, Levine D, Shen J, Gogarten SM, Laurie C, Weir BS. 2012.** A high-performance computing toolset for relatedness and principal component analysis of SNP data. *Bioinformatics* **28**(24): 3326-3328.

**Table S2** Genome size of wild Brassica relatives determined using flow cytometry. Genome sizes were determined from the same individuals used for whole genome sequencing.

| **Species** | **Accession** | **Chromosome number (est.)** | **DNA content** | |  |
| --- | --- | --- | --- | --- | --- |
|  |  |  | **pg/2C** | **Mbp/2C** | **Mbp/1C** |
| *Brassica cretica* | PI662593 | 18 | 1.30 | 1277 | 639 |
| *Brassica incana* | PI662591 | 18 | 1.31 | 1281 | 641 |
| *Brassica macrocarpa* | HRI08:013115A | 18 | 1.24 | 1219 | 610 |
| *Brassica montana* | CGN18472 | 18 | 1.30 | 1272 | 636 |
| *Brassica rupestris* | BRA2945 | 18 | 1.26 | 1230 | 615 |
| *Brassica villosa* | K10263 | 18 | 1.32 | 1290 | 645 |

**Table S3** Statistical analysis of differences in relative minimum distances between domesticated *B. oleracea* varieties and wild *Brassica* relatives. Relative minimum distance (RNDmin) between domesticated varieties (combined) and each wild relative was calculated in 50 kb windows with a 50 kb step size. Comparison of the distribution of RNDmin values for each pair were analysed in two parts due to the presence of zero RNDmin values. Firstly one-way ANOVA was conducted on log-transformed RNDmin values when zero values were removed, with post hoc Tukey’s HSD test to adjust for multiple comparisons. Secondly the probability of windows with zero RNDmin was compared across species using logistic regression.

| **One-way ANOVA with Tukey’s HSD of non-zero windows** | | | | | |
| --- | --- | --- | --- | --- | --- |
| Species comparison | Difference in observed means | Lower bound | Upper bound | Adjusted p-value | |
| *B. insularis* – *B. cretica* | 0.231 | 0.173 | 0.288 | 0.000 | |
| *B. macrocarpa* – *B. cretica* | 0.611 | 0.559 | 0.662 | 0.000 | |
| *B. rupestris* – *B. cretica* | 0.570 | 0.517 | 0.622 | 0.000 | |
| *B. macrocarpa* – *B. insularis* | 0.380 | 0.329 | 0.431 | 0.000 | |
| *B. rupestris* – *B. insularis* | 0.339 | 0.287 | 0.391 | 0.000 | |
| *B. rupestris* – *B. macrocarpa* | -0.041 | -0.086 | 0.004 | 0.089 | |
|  |  |  |  | | |
| **Logistic regression with post hoc Tukey’s HSD of log odds ratios** | | | | | |
|  | Odds ratio | Standard Error | Degrees of freedom | Z ratio | Adjusted p -value |
| *B. cretica* – *B. insularis* | 0.902 | 0.034 | Inf | -2.765 | 0.0291 |
| *B. cretica* – *B. macrocarpa* | 0.334 | 0.012 | Inf | -29.68 | <.0001 |
| *B. cretica* – *B. rupestris* | 0.422 | 0.016 | Inf | -23.52 | <.0001 |
| *B. insularis* – *B. macrocarpa* | 0.370 | 0.014 | Inf | -26.82 | <.0001 |
| *B. insularis* – *B. rupestris* | 0.468 | 0.017 | Inf | -20.66 | <.0001 |
| *B. macrocarpa* – *B. rupestris* | 1.262 | 0.046 | Inf | 6.39 | <.0001 |

**Table S4** Statistical analysis of differences in relative minimum distances between domesticated *B. rapa* varieties and wild *Brassica* relatives. Relative minimum distance (RNDmin) between domesticated varieties (combined) and each wild relative was calculated in 50 kb windows with a 50 kb step size. Comparison of the distribution of RNDmin values for each pair were analysed using a one-way ANOVA, conducted on log-transformed RNDmin values, with posthoc Tukey’s HSD test to adjust for multiple comparisons.

| **One-way ANOVA with Tukey’s HSD of non-conserved windows** | | | | |
| --- | --- | --- | --- | --- |
| Species comparison | Difference in observed means | Lower bound | Upper bound | Adjusted p-value |
| *B. insularis* – *B. cretica* | -0.365 | -0.395 | -0.334 | 0.000 |
| *B. macrocarpa* – *B. cretica* | -0.020 | -0.050 | 0.010 | 0.299 |
| *B. rupestris* – *B. cretica* | -0.279 | -0.309 | -0.249 | 0.000 |
| *B. macrocarpa* – *B. insularis* | 0.344 | 0.314 | 0.375 | 0.000 |
| *B. rupestris* – *B. insularis* | 0.085 | 0.055 | 0.116 | 0.000 |
| *B. rupestris* – *B. macrocarpa* | -0.259 | -0.289 | -0.229 | 0.000 |

**Table S5** D-statistics used to test for signals of introgression between *Brassica oleracea* varieties and wild *Brassica* relatives. For each pair of populations denoted P2 and P3, reported is the D-statistic that had the lowest p-value when all possible P1 populations are considered. P-values are corrected for multiple testing using Benjamini-Hochberg multiple test adjustment.

| **P2** | **P3** | **D-statistic** | **P-value** |
| --- | --- | --- | --- |
| *B. oleracea* var. *capitata* | *B. oleracea* var. *botrytis* | 0.022 | 0.062 |
| *B. oleracea* var. *capitata* | *B. oleracea* var. *gongylodes* | 0.000 | 1.000 |
| *B. oleracea* var. *capitata* | *B. oleracea* var. *alboglabra* | 0.053 | 0.000 |
| *B. oleracea* var. *capitata* | Wild *B. oleracea 1* | 0.002 | 0.453 |
| *B. oleracea* var. *capitata* | Wild *B. oleracea 2* | 0.000 | 1.000 |
| *B. oleracea* var. *capitata* | *B. cretica* | 0.071 | 0.000 |
| *B. oleracea* var. *capitata* | *B. insularis* | 0.128 | 0.000 |
| *B. oleracea* var. *capitata* | *B. macrocarpa* | 0.162 | 0.000 |
| *B. oleracea* var. *capitata* | *B. rupestris* | 0.172 | 0.000 |
| *B. oleracea* var. *botrytis* | *B. oleracea* var. *gongylodes* | 0.039 | 0.034 |
| *B. oleracea* var. *botrytis* | *B. oleracea* var. *alboglabra* | 0.048 | 0.000 |
| *B. oleracea* var. *botrytis* | Wild *B. oleracea 1* | 0.000 | 1.000 |
| *B. oleracea* var. *botrytis* | Wild *B. oleracea 2* | 0.004 | 0.438 |
| *B. oleracea* var. *botrytis* | *B. cretica* | 0.099 | 0.000 |
| *B. oleracea* var. *botrytis* | *B. insularis* | 0.123 | 0.000 |
| *B. oleracea* var. *botrytis* | *B. macrocarpa* | 0.162 | 0.000 |
| *B. oleracea* var. *botrytis* | *B. rupestris* | 0.158 | 0.000 |
| *B. oleracea* var. *gongylodes* | *B. oleracea* var. *alboglabra* | 0.043 | 0.000 |
| *B. oleracea* var. *gongylodes* | Wild *B. oleracea 1* | 0.041 | 0.004 |
| *B. oleracea* var. *gongylodes* | Wild *B. oleracea 2* | 0.013 | 0.256 |
| *B. oleracea* var. *gongylodes* | *B. cretica* | 0.070 | 0.000 |
| *B. oleracea* var. *gongylodes* | *B. insularis* | 0.140 | 0.000 |
| *B. oleracea* var. *gongylodes* | *B. macrocarpa* | 0.182 | 0.000 |
| *B. oleracea* var. *gongylodes* | *B. rupestris* | 0.183 | 0.000 |
| *B. oleracea* var. *alboglabra* | Wild *B. oleracea 1* | 0.000 | 1.000 |
| *B. oleracea* var. *alboglabra* | Wild *B. oleracea 2* | 0.004 | 0.360 |
| *B. oleracea* var. *alboglabra* | *B. cretica* | 0.063 | 0.000 |
| *B. oleracea* var. *alboglabra* | *B. insularis* | 0.054 | 0.000 |
| *B. oleracea* var. *alboglabra* | *B. macrocarpa* | 0.151 | 0.000 |
| *B. oleracea* var. *alboglabra* | *B. rupestris* | 0.000 | 1.000 |
| Wild *B. oleracea 1* | Wild *B. oleracea 2* | 0.031 | 0.067 |
| Wild *B. oleracea 1* | *B. cretica* | 0.056 | 0.003 |
| Wild *B. oleracea 1* | *B. insularis* | 0.140 | 0.000 |
| Wild *B. oleracea 1* | *B. macrocarpa* | 0.178 | 0.000 |
| Wild *B. oleracea 1* | *B. rupestris* | 0.182 | 0.000 |
| Wild *B. oleracea 2* | *B. cretica* | 0.058 | 0.000 |
| Wild *B. oleracea 2* | *B. insularis* | 0.154 | 0.000 |
| Wild *B. oleracea 2* | *B. macrocarpa* | 0.189 | 0.000 |
| Wild *B. oleracea 2* | *B. rupestris* | 0.214 | 0.000 |
| *B. cretica* | *B. insularis* | 0.048 | 0.000 |
| *B. cretica* | *B. macrocarpa* | 0.089 | 0.000 |
| *B. cretica* | *B. rupestris* | 0.154 | 0.000 |
| *B. insularis* | *B. macrocarpa* | 0.000 | 1.000 |
| *B. insularis* | *B. rupestris* | 0.108 | 0.000 |
| *B. macrocarpa* | *B. rupestris* | 0.119 | 0.000 |

**Table S6** Estimated genome-wide proportion of introgressed sites using the *fd* statistic in *Brassica oleracea* analyses. Reported is the average *fd* statistic when each possible P1 population is considered; all other *B. oleracea* populations as P1 when P2 is a domesticated variety and P3 is a CWR, and all other CWRs as P1 when P2 is a CWR and P3 is a *B. oleracea* population.

| **P2** | **P3** | ***fd*** |
| --- | --- | --- |
| *B. oleracea* var. *alboglabra* | *B. cretica* | -0.032 |
| *B. oleracea* var. *alboglabra* | *B. insularis* | -0.066 |
| *B. oleracea* var. *alboglabra* | *B. macrocarpa* | -0.063 |
| *B. oleracea* var. *alboglabra* | *B. rupestris* | -0.061 |
| *B. oleracea* var. *botrytis* | *B. cretica* | 0.036 |
| *B. oleracea* var. *botrytis* | *B. insularis* | 0.002 |
| *B. oleracea* var. *botrytis* | *B. macrocarpa* | 0.005 |
| *B. oleracea* var. *botrytis* | *B. rupestris* | 0.000 |
| *B. oleracea* var. *capitata* | *B. cretica* | 0.002 |
| *B. oleracea* var. *capitata* | *B. insularis* | 0.003 |
| *B. oleracea* var. *capitata* | *B. macrocarpa* | 0.003 |
| *B. oleracea* var. *capitata* | *B. rupestris* | 0.005 |
| *B. oleracea* var. *gongylodes* | *B. cretica* | 0.015 |
| *B. oleracea* var. *gongylodes* | *B. insularis* | 0.013 |
| *B. oleracea* var. *gongylodes* | *B. macrocarpa* | 0.014 |
| *B. oleracea* var. *gongylodes* | *B. rupestris* | 0.011 |
| Wild *B. oleracea* 1 | *B. cretica* | -0.016 |
| Wild *B. oleracea* 1 | *B. insularis* | 0.016 |
| Wild *B. oleracea* 1 | *B. macrocarpa* | 0.015 |
| Wild *B. oleracea* 1 | *B. rupestris* | 0.031 |
| Wild *B. oleracea* 2 | *B. cretica* | -0.015 |
| Wild *B. oleracea* 2 | *B. insularis* | 0.031 |
| Wild *B. oleracea* 2 | *B. macrocarpa* | 0.024 |
| Wild *B. oleracea* 2 | *B. rupestris* | 0.031 |
| *B. cretica* | *B. oleracea* var. *alboglabra* | 0.143 |
| *B. cretica* | *B. oleracea* var. *botrytis* | 0.128 |
| *B. cretica* | *B. oleracea* var. *capitata* | 0.106 |
| *B. cretica* | *B. oleracea* var. *gongylodes* | 0.105 |
| *B. cretica* | Wild *B. oleracea* 1 | 0.101 |
| *B. cretica* | Wild *B. oleracea* 2 | 0.095 |
| *B. insularis* | *B. oleracea* var. *alboglabra* | -0.003 |
| *B. insularis* | *B. oleracea* var. *botrytis* | -0.012 |
| *B. insularis* | *B. oleracea* var. *capitata* | -0.004 |
| *B. insularis* | *B. oleracea* var. *gongylodes* | -0.005 |
| *B. insularis* | Wild *B. oleracea* 1 | 0.002 |
| *B. insularis* | Wild *B. oleracea* 2 | 0.002 |
| *B. macrocarpa* | *B. oleracea* var. *alboglabra* | -0.027 |
| *B. macrocarpa* | *B. oleracea* var. *botrytis* | -0.016 |
| *B. macrocarpa* | *B. oleracea* var. *capitata* | -0.012 |
| *B. macrocarpa* | *B. oleracea* var. *gongylodes* | -0.010 |
| *B. macrocarpa* | Wild *B. oleracea* 1 | -0.006 |
| *B. macrocarpa* | Wild *B. oleracea* 2 | 0.002 |
| *B. rupestris* | *B. oleracea* var. *alboglabra* | -0.094 |
| *B. rupestris* | *B. oleracea* var. *botrytis* | -0.086 |
| *B. rupestris* | *B. oleracea* var. *capitata* | -0.077 |
| *B. rupestris* | *B. oleracea* var. *gongylodes* | -0.077 |
| *B. rupestris* | Wild *B. oleracea* 1 | -0.081 |
| *B. rupestris* | Wild *B. oleracea* 2 | -0.074 |

**Table S7** ABC model checking for Scenario 4 in network analysis of *Brassica oleracea* phylogenies. Summary statistics included: the mean and variance of gene diversity on polymorphic loci (HM1 and HV1 respectively), mean gene diversity on all loci (HMO), mean and variance of non-zero Fst distances (FM1 and FV1 respectively), mean of all Fst distances (FMO). Population *j* in summary statistic format STAT_1_*j* corresponds to populations 1-9 in Figure S6. Tail-area probability significance is indicated as *, *P* < 0.05; **, *P* < 0.01; ***, *P* < 0.001.

| Summary statistics | Observed value | proportion (simulated<observed) | |
| --- | --- | --- | --- |
| HM1_1_1 | 0.4115 | 0.9943 | (**) |
| HM1_1_2 | 0.5787 | 0.9926 | (**) |
| HM1_1_3 | 0.5633 | 0.9801 | (*) |
| HM1_1_4 | 0.3753 | 0.9893 | (*) |
| HM1_1_5 | 0.4334 | 0.9908 | (**) |
| HM1_1_6 | 0.4443 | 0.9955 | (**) |
| HM1_1_7 | 0.3518 | 0.9826 | (*) |
| HM1_1_8 | 0.3671 | 0.996 | (**) |
| HM1_1_9 | 0.418 | 0.9821 | (*) |
| HV1_1_1 | 0.0162 | 0.0938 |  |
| HV1_1_2 | 0.0069 | 0.9054 |  |
| HV1_1_3 | 0.0066 | 0.8251 |  |
| HV1_1_4 | 0.0186 | 0.2084 |  |
| HV1_1_5 | 0.0138 | 0.0652 |  |
| HV1_1_6 | 0.0122 | 0.0259 | (*) |
| HV1_1_7 | 0.0199 | 0.204 |  |
| HV1_1_8 | 0.0189 | 0.1129 |  |
| HV1_1_9 | 0.0155 | 0.3192 |  |
| HMO_1_1 | 0.1651 | 1 | (***) |
| HMO_1_2 | 0.189 | 1 | (***) |
| HMO_1_3 | 0.2467 | 1 | (***) |
| HMO_1_4 | 0.2573 | 1 | (***) |
| HMO_1_5 | 0.1246 | 0.9864 | (*) |
| HMO_1_6 | 0.1819 | 1 | (***) |
| HMO_1_7 | 0.188 | 1 | (***) |
| HMO_1_8 | 0.1828 | 1 | (***) |
| HMO_1_9 | 0.0843 | 0.856 |  |
| FM1_1_1&2 | 0.436 | 0.2747 |  |
| FM1_1_1&3 | 0.4172 | 0.162 |  |
| FM1_1_1&4 | 0.2944 | 0.2881 |  |
| FM1_1_1&5 | 0.365 | 0.7241 |  |
| FM1_1_1&6 | 0.3633 | 0.6041 |  |
| FM1_1_1&7 | 0.3228 | 0.3763 |  |
| FM1_1_1&8 | 0.358 | 0.5667 |  |
| FM1_1_1&9 | 0.4546 | 0.7819 |  |
| FM1_1_2&3 | 0.4247 | 0.0233 | (*) |
| FM1_1_2&4 | 0.2413 | 0 | (***) |
| FM1_1_2&5 | 0.4089 | 0.694 |  |
| FM1_1_2&6 | 0.3469 | 0.457 |  |
| FM1_1_2&7 | 0.3592 | 0.0417 | (*) |
| FM1_1_2&8 | 0.3586 | 0.0541 |  |
| FM1_1_2&9 | 0.5273 | 0.8832 |  |
| FM1_1_3&4 | 0.2513 | 0.0381 | (*) |
| FM1_1_3&5 | 0.3963 | 0.7249 |  |
| FM1_1_3&6 | 0.3494 | 0.5833 |  |
| FM1_1_3&7 | 0.3594 | 0.3698 |  |
| FM1_1_3&8 | 0.3911 | 0.4646 |  |
| FM1_1_3&9 | 0.4795 | 0.8136 |  |
| FM1_1_4&5 | 0.2417 | 0.2686 |  |
| FM1_1_4&6 | 0.2314 | 0.2681 |  |
| FM1_1_4&7 | 0.1751 | 0.1929 |  |
| FM1_1_4&8 | 0.2141 | 0.4182 |  |
| FM1_1_4&9 | 0.3455 | 0.6285 |  |
| FM1_1_5&6 | 0.3693 | 0.4376 |  |
| FM1_1_5&7 | 0.2885 | 0.4875 |  |
| FM1_1_5&8 | 0.3104 | 0.64 |  |
| FM1_1_5&9 | 0.555 | 0.8826 |  |
| FM1_1_6&7 | 0.2872 | 0.5282 |  |
| FM1_1_6&8 | 0.3038 | 0.656 |  |
| FM1_1_6&9 | 0.4964 | 0.777 |  |
| FM1_1_7&8 | 0.2553 | 0.5928 |  |
| FM1_1_7&9 | 0.3905 | 0.7408 |  |
| FM1_1_8&9 | 0.4106 | 0.809 |  |
| FV1_1_1&2 | 0.0834 | 0.2629 |  |
| FV1_1_1&3 | 0.0718 | 0.0811 |  |
| FV1_1_1&4 | 0.0611 | 0.0474 | (*) |
| FV1_1_1&5 | 0.0823 | 0.5025 |  |
| FV1_1_1&6 | 0.0855 | 0.3322 |  |
| FV1_1_1&7 | 0.0796 | 0.174 |  |
| FV1_1_1&8 | 0.0832 | 0.2442 |  |
| FV1_1_1&9 | 0.1164 | 0.623 |  |
| FV1_1_2&3 | 0.0433 | 0.069 |  |
| FV1_1_2&4 | 0.0447 | 0.0511 |  |
| FV1_1_2&5 | 0.0826 | 0.4171 |  |
| FV1_1_2&6 | 0.0834 | 0.4518 |  |
| FV1_1_2&7 | 0.0717 | 0.3841 |  |
| FV1_1_2&8 | 0.0725 | 0.423 |  |
| FV1_1_2&9 | 0.1056 | 0.6182 |  |
| FV1_1_3&4 | 0.0455 | 0.3291 |  |
| FV1_1_3&5 | 0.0723 | 0.4095 |  |
| FV1_1_3&6 | 0.07 | 0.4128 |  |
| FV1_1_3&7 | 0.0672 | 0.6604 |  |
| FV1_1_3&8 | 0.0686 | 0.6152 |  |
| FV1_1_3&9 | 0.0969 | 0.5166 |  |
| FV1_1_4&5 | 0.0521 | 0.212 |  |
| FV1_1_4&6 | 0.0471 | 0.1951 |  |
| FV1_1_4&7 | 0.023 | 0.0483 | (*) |
| FV1_1_4&8 | 0.0372 | 0.2354 |  |
| FV1_1_4&9 | 0.0953 | 0.5777 |  |
| FV1_1_5&6 | 0.071 | 0.5406 |  |
| FV1_1_5&7 | 0.0743 | 0.5052 |  |
| FV1_1_5&8 | 0.0794 | 0.619 |  |
| FV1_1_5&9 | 0.1141 | 0.9069 |  |
| FV1_1_6&7 | 0.0653 | 0.4501 |  |
| FV1_1_6&8 | 0.067 | 0.5422 |  |
| FV1_1_6&9 | 0.105 | 0.8152 |  |
| FV1_1_7&8 | 0.0506 | 0.6004 |  |
| FV1_1_7&9 | 0.1201 | 0.7571 |  |
| FV1_1_8&9 | 0.1191 | 0.767 |  |
| FMO_1_1&2 | 0.1864 | 0.9884 | (*) |
| FMO_1_1&3 | 0.2012 | 0.9951 | (**) |
| FMO_1_1&4 | 0.181 | 0.9845 | (*) |
| FMO_1_1&5 | 0.1689 | 0.9883 | (*) |
| FMO_1_1&6 | 0.1933 | 0.995 | (**) |
| FMO_1_1&7 | 0.2016 | 0.9934 | (**) |
| FMO_1_1&8 | 0.2117 | 0.9964 | (**) |
| FMO_1_1&9 | 0.2278 | 0.9992 | (***) |
| FMO_1_2&3 | 0.0991 | 0.9714 | (*) |
| FMO_1_2&4 | 0.0757 | 0.6141 |  |
| FMO_1_2&5 | 0.1421 | 0.9868 | (*) |
| FMO_1_2&6 | 0.1313 | 0.9806 | (*) |
| FMO_1_2&7 | 0.1176 | 0.884 |  |
| FMO_1_2&8 | 0.122 | 0.9092 |  |
| FMO_1_2&9 | 0.2524 | 1 | (***) |
| FMO_1_3&4 | 0.0715 | 0.9177 |  |
| FMO_1_3&5 | 0.1598 | 0.9977 | (**) |
| FMO_1_3&6 | 0.1317 | 0.99 | (*) |
| FMO_1_3&7 | 0.1352 | 0.9981 | (**) |
| FMO_1_3&8 | 0.1472 | 0.9988 | (**) |
| FMO_1_3&9 | 0.2685 | 1 | (***) |
| FMO_1_4&5 | 0.1179 | 0.9238 |  |
| FMO_1_4&6 | 0.1043 | 0.8946 |  |
| FMO_1_4&7 | 0.0781 | 0.7852 |  |
| FMO_1_4&8 | 0.1127 | 0.9761 | (*) |
| FMO_1_4&9 | 0.2239 | 1 | (***) |
| FMO_1_5&6 | 0.13 | 0.9763 | (*) |
| FMO_1_5&7 | 0.1342 | 0.9565 | (*) |
| FMO_1_5&8 | 0.1422 | 0.9781 | (*) |
| FMO_1_5&9 | 0.2259 | 1 | (***) |
| FMO_1_6&7 | 0.1338 | 0.9772 | (*) |
| FMO_1_6&8 | 0.1433 | 0.991 | (**) |
| FMO_1_6&9 | 0.251 | 1 | (***) |
| FMO_1_7&8 | 0.1164 | 0.9986 | (**) |
| FMO_1_7&9 | 0.2369 | 1 | (***) |
| FMO_1_8&9 | 0.245 | 1 | (***) |

**Table S8** D-statistics used to test for signals of introgression between *Brassica rapa* varieties and wild *Brassica* relatives.

| **P2** | **P3** | **D-statistic** | **P-value** |
| --- | --- | --- | --- |
| *B. rapa* ssp. *chinensis* | *B. rapa* ssp. *parachinensis* | 0.000 | 1.000 |
| *B. rapa* ssp. *chinensis* | *B. rapa* ssp. *pekinensis* | 0.103 | 0.000 |
| *B. rapa* ssp. *chinensis* | *B. rapa* ssp. *trilocularis* | 0.050 | 0.000 |
| *B. rapa* ssp. *chinensis* | Wild *B. rapa* and *B. rapa* ssp. *rapa* | 0.000 | 1.000 |
| *B. rapa* ssp. *chinensis* | *B. cretica* | 0.121 | 0.000 |
| *B. rapa* ssp. *chinensis* | *B. insularis* | 0.099 | 0.000 |
| *B. rapa* ssp. *chinensis* | *B. macrocarpa* | 0.118 | 0.000 |
| *B. rapa* ssp. *chinensis* | *B. rupestris* | 0.117 | 0.000 |
| *B. rapa* ssp. *parachinensis* | *B. rapa* ssp. *pekinensis* | 0.041 | 0.000 |
| *B. rapa* ssp. *parachinensis* | *B. rapa* ssp. *trilocularis* | 0.069 | 0.000 |
| *B. rapa* ssp. *parachinensis* | Wild *B. rapa* and *B. rapa* ssp. *rapa* | 0.041 | 0.000 |
| *B. rapa* ssp. *parachinensis* | *B. cretica* | 0.070 | 0.000 |
| *B. rapa* ssp. *parachinensis* | *B. insularis* | 0.058 | 0.000 |
| *B. rapa* ssp. *parachinensis* | *B. macrocarpa* | 0.073 | 0.000 |
| *B. rapa* ssp. *parachinensis* | *B. rupestris* | 0.068 | 0.000 |
| *B. rapa* ssp. *pekinensis* | *B. rapa* ssp. *trilocularis* | 0.000 | 1.000 |
| *B. rapa* ssp. *pekinensis* | Wild *B. rapa* and *B. rapa* ssp. *rapa* | 0.003 | 0.400 |
| *B. rapa* ssp. *pekinensis* | *B. cretica* | 0.000 | 1.000 |
| *B. rapa* ssp. *pekinensis* | *B. insularis* | 0.108 | 0.000 |
| *B. rapa* ssp. *pekinensis* | *B. macrocarpa* | 0.001 | 0.446 |
| *B. rapa* ssp. *pekinensis* | *B. rupestris* | 0.030 | 0.000 |
| *B. rapa* ssp. *trilocularis* | Wild *B. rapa* and *B. rapa* ssp. *rapa* | 0.000 | 1.000 |
| *B. rapa* ssp. *trilocularis* | *B. cretica* | 0.139 | 0.000 |
| *B. rapa* ssp. *trilocularis* | *B. insularis* | 0.107 | 0.000 |
| *B. rapa* ssp. *trilocularis* | *B. macrocarpa* | 0.132 | 0.000 |
| *B. rapa* ssp. *trilocularis* | *B. rupestris* | 0.130 | 0.000 |
| Wild *B. rapa* and *B. rapa* ssp. *rapa* | *B. cretica* | 0.135 | 0.000 |
| Wild *B. rapa* and *B. rapa* ssp. *rapa* | *B. insularis* | 0.111 | 0.000 |
| Wild *B. rapa* and *B. rapa* ssp. *rapa* | *B. macrocarpa* | 0.132 | 0.000 |
| Wild *B. rapa* and *B. rapa* ssp. *rapa* | *B. rupestris* | 0.130 | 0.000 |
| *B. cretica* | *B. insularis* | 0.025 | 0.006 |
| *B. cretica* | *B. macrocarpa* | 0.011 | 0.196 |
| *B. cretica* | *B. rupestris* | 0.000 | 1.000 |
| *B. insularis* | *B. macrocarpa* | 0.050 | 0.000 |
| *B. insularis* | *B. rupestris* | 0.000 | 1.000 |
| *B. macrocarpa* | *B. rupestris* | 0.129 | 0.000 |

**Table S9** Estimated genome-wide proportion of introgressed sites using the *fd* statistic in *Brassica rapa* analyses. Reported is the average *fd* statistic when each possible P1 population is considered; all other domesticated varieties as P1 when P2 is a domesticated variety and P3 is a CWR, and all other CWRs as P1 when P2 is a CWR and P3 is a domesticated variety.

| **P2** | **P3** | ***fd*** |
| --- | --- | --- |
| *B. rapa* ssp. *chinensis* | *B. cretica* | -0.010 |
| *B. rapa* ssp. *chinensis* | *B. insularis* | -0.010 |
| *B. rapa* ssp. *chinensis* | *B. macrocarpa* | -0.010 |
| *B. rapa* ssp. *chinensis* | *B. rupestris* | -0.010 |
| *B. rapa* ssp. *parachinensis* | *B. cretica* | 0.000 |
| *B. rapa* ssp. *parachinensis* | *B. insularis* | 0.001 |
| *B. rapa* ssp. *parachinensis* | *B. macrocarpa* | 0.001 |
| *B. rapa* ssp. *parachinensis* | *B. rupestris* | 0.000 |
| *B. rapa* ssp. *pekinensis* | *B. cretica* | -0.032 |
| *B. rapa* ssp. *pekinensis* | *B. insularis* | -0.030 |
| *B. rapa* ssp. *pekinensis* | *B. macrocarpa* | -0.030 |
| *B. rapa* ssp. *pekinensis* | *B. rupestris* | -0.030 |
| *B. rapa* ssp. *trilocularis* | *B. cretica* | 0.018 |
| *B. rapa* ssp. *trilocularis* | *B. insularis* | 0.015 |
| *B. rapa* ssp. *trilocularis* | *B. macrocarpa* | 0.016 |
| *B. rapa* ssp. *trilocularis* | *B. rupestris* | -0.030 |
| Wild *B. rapa* / ssp. *rapa* | *B. cretica* | 0.018 |
| Wild *B. rapa* / ssp. *rapa* | *B. insularis* | 0.017 |
| Wild *B. rapa* / ssp. *rapa* | *B. macrocarpa* | 0.017 |
| Wild *B. rapa* / ssp. *rapa* | *B. rupestris* | 0.018 |
| *B. cretica* | *B. rapa* ssp. *chinensis* | -0.002 |
| *B. cretica* | *B. rapa* ssp. *parachinensis* | -0.002 |
| *B. cretica* | *B. rapa* ssp. *pekinensis* | -0.004 |
| *B. cretica* | *B. rapa* ssp. *trilocularis* | 0.000 |
| *B. cretica* | Wild *B. rapa* / ssp. *rapa* | -0.002 |
| *B. insularis* | *B. rapa* ssp. *chinensis* | 0.007 |
| *B. insularis* | *B. rapa* ssp. *parachinensis* | 0.005 |
| *B. insularis* | *B. rapa* ssp. *pekinensis* | 0.009 |
| *B. insularis* | *B. rapa* ssp. *trilocularis* | 0.002 |
| *B. insularis* | Wild *B. rapa* / ssp. *rapa* | 0.004 |
| *B. macrocarpa* | *B. rapa* ssp. *chinensis* | -0.003 |
| *B. macrocarpa* | *B. rapa* ssp. *parachinensis* | -0.002 |
| *B. macrocarpa* | *B. rapa* ssp. *pekinensis* | -0.004 |
| *B. macrocarpa* | *B. rapa* ssp. *trilocularis* | -0.002 |
| *B. macrocarpa* | Wild *B. rapa* / ssp. *rapa* | -0.001 |
| *B. rupestris* | *B. rapa* ssp. *chinensis* | 0.000 |
| *B. rupestris* | *B. rapa* ssp. *parachinensis* | 0.000 |
| *B. rupestris* | *B. rapa* ssp. *pekinensis* | 0.000 |
| *B. rupestris* | *B. rapa* ssp. *trilocularis* | 0.000 |
| *B. rupestris* | Wild *B. rapa* / ssp. *rapa* | 0.000 |
| *B. rapa* ssp. *chinensis* | *B. rupestris* | -0.010 |
| *B. rapa* ssp. *parachinensis* | *B. cretica* | 0.000 |

**Table S10** ABC model checking for Scenario 3 in network analysis of one reticulation *Brassica rapa* phylogenies. Summary statistics included: the proportion of monomorphic loci (HP0), mean and variance of gene diversity on polymorphic loci (HM1 and HV1 respectively), mean gene diversity on all loci (HMO), proportion of loci with null Fst distances (FP0), mean and variance of non-zero Fst distances (FM1 and FV1 respectively), mean of all Fst distances (FMO), proportion of loci with null Nei’s distances (NP0), mean and variance of non-zero Nei’s distances (NM1 and NV1 respectively) and mean of all Nei’s distances (NMO). Population *j* in summary statistic format STAT_1_*j* corresponds to populations 1-6 in Figure S9. Tail-area probability significance is indicated as *, *P* < 0.05; **, *P* < 0.01; ***, *P* < 0.001.

| Summary statistics | Observed value | proportion (simulated<observed) | |
| --- | --- | --- | --- |
| HP0_1_1 | 0.678 | 1.000 | (***) |
| HP0_1_2 | 0.952 | 0.108 |  |
| HP0_1_3 | 0.774 | 0.003 | (**) |
| HP0_1_4 | 0.761 | 0.000 | (***) |
| HP0_1_5 | 0.678 | 0.000 | (***) |
| HP0_1_6 | 0.432 | 0.000 | (***) |
| HM1_1_1 | 0.395 | 1.000 | (***) |
| HM1_1_2 | 0.443 | 0.897 |  |
| HM1_1_3 | 0.355 | 0.977 | (*) |
| HM1_1_4 | 0.491 | 0.986 | (*) |
| HM1_1_5 | 0.367 | 0.800 |  |
| HM1_1_6 | 0.325 | 1.000 | (***) |
| HV1_1_1 | 0.018 | 0.271 |  |
| HV1_1_2 | 0.016 | 0.423 |  |
| HV1_1_3 | 0.014 | 0.002 | (**) |
| HV1_1_4 | 0.009 | 0.005 | (**) |
| HV1_1_5 | 0.017 | 0.043 | (*) |
| HV1_1_6 | 0.021 | 0.013 | (*) |
| HMO_1_1 | 0.127 | 0.000 | (***) |
| HMO_1_2 | 0.022 | 0.902 |  |
| HMO_1_3 | 0.080 | 0.996 | (**) |
| HMO_1_4 | 0.117 | 1.000 | (***) |
| HMO_1_5 | 0.118 | 1.000 | (***) |
| HMO_1_6 | 0.185 | 1.000 | (***) |
| FP0_1_1&2 | 0.575 | 0.997 | (**) |
| FP0_1_1&3 | 0.434 | 0.999 | (***) |
| FP0_1_1&4 | 0.485 | 0.970 | (*) |
| FP0_1_1&5 | 0.361 | 0.994 | (**) |
| FP0_1_1&6 | 0.210 | 0.886 |  |
| FP0_1_2&3 | 0.681 | 0.010 | (**) |
| FP0_1_2&4 | 0.665 | 0.000 | (***) |
| FP0_1_2&5 | 0.621 | 0.001 | (***) |
| FP0_1_2&6 | 0.471 | 0.000 | (***) |
| FP0_1_3&4 | 0.770 | 0.001 | (***) |
| FP0_1_3&5 | 0.674 | 0.000 | (***) |
| FP0_1_3&6 | 0.532 | 0.000 | (***) |
| FP0_1_4&5 | 0.775 | 0.001 | (***) |
| FP0_1_4&6 | 0.633 | 0.000 | (***) |
| FP0_1_5&6 | 0.519 | 0.000 | (***) |
| FM1_1_1&2 | 0.558 | 0.897 |  |
| FM1_1_1&3 | 0.304 | 0.001 | (***) |
| FM1_1_1&4 | 0.357 | 0.000 | (***) |
| FM1_1_1&5 | 0.330 | 0.000 | (***) |
| FM1_1_1&6 | 0.301 | 0.003 | (**) |
| FM1_1_2&3 | 0.463 | 0.054 |  |
| FM1_1_2&4 | 0.522 | 0.062 |  |
| FM1_1_2&5 | 0.434 | 0.034 | (*) |
| FM1_1_2&6 | 0.325 | 0.007 | (**) |
| FM1_1_3&4 | 0.351 | 0.620 |  |
| FM1_1_3&5 | 0.286 | 0.232 |  |
| FM1_1_3&6 | 0.180 | 0.185 |  |
| FM1_1_4&5 | 0.312 | 0.070 |  |
| FM1_1_4&6 | 0.218 | 0.000 | (***) |
| FM1_1_5&6 | 0.221 | 0.113 |  |
| FV1_1_1&2 | 0.126 | 0.134 |  |
| FV1_1_1&3 | 0.082 | 0.001 | (***) |
| FV1_1_1&4 | 0.078 | 0.000 | (***) |
| FV1_1_1&5 | 0.081 | 0.000 | (***) |
| FV1_1_1&6 | 0.070 | 0.002 | (**) |
| FV1_1_2&3 | 0.150 | 0.698 |  |
| FV1_1_2&4 | 0.105 | 0.286 |  |
| FV1_1_2&5 | 0.120 | 0.511 |  |
| FV1_1_2&6 | 0.104 | 0.052 |  |
| FV1_1_3&4 | 0.041 | 0.099 |  |
| FV1_1_3&5 | 0.051 | 0.171 |  |
| FV1_1_3&6 | 0.027 | 0.027 | (*) |
| FV1_1_4&5 | 0.044 | 0.028 | (*) |
| FV1_1_4&6 | 0.036 | 0.009 | (**) |
| FV1_1_5&6 | 0.040 | 0.030 | (*) |
| FMO_1_1&2 | 0.237 | 0.014 | (*) |
| FMO_1_1&3 | 0.172 | 0.000 | (***) |
| FMO_1_1&4 | 0.185 | 0.002 | (**) |
| FMO_1_1&5 | 0.211 | 0.001 | (***) |
| FMO_1_1&6 | 0.238 | 0.003 | (**) |
| FMO_1_2&3 | 0.147 | 0.954 | (*) |
| FMO_1_2&4 | 0.175 | 0.983 | (*) |
| FMO_1_2&5 | 0.164 | 0.979 | (*) |
| FMO_1_2&6 | 0.172 | 0.979 | (*) |
| FMO_1_3&4 | 0.081 | 0.998 | (**) |
| FMO_1_3&5 | 0.093 | 0.999 | (***) |
| FMO_1_3&6 | 0.084 | 0.997 | (**) |
| FMO_1_4&5 | 0.070 | 0.990 | (**) |
| FMO_1_4&6 | 0.080 | 0.997 | (**) |
| FMO_1_5&6 | 0.106 | 0.999 | (***) |
| NP0_1_1&2 | 0.493 | 1.000 | (***) |
| NP0_1_1&3 | 0.419 | 0.999 | (***) |
| NP0_1_1&4 | 0.404 | 0.997 | (**) |
| NP0_1_1&5 | 0.324 | 0.992 | (**) |
| NP0_1_1&6 | 0.095 | 0.075 |  |
| NP0_1_2&3 | 0.663 | 0.011 | (*) |
| NP0_1_2&4 | 0.654 | 0.005 | (**) |
| NP0_1_2&5 | 0.585 | 0.001 | (***) |
| NP0_1_2&6 | 0.374 | 0.000 | (***) |
| NP0_1_3&4 | 0.640 | 0.000 | (***) |
| NP0_1_3&5 | 0.598 | 0.000 | (***) |
| NP0_1_3&6 | 0.430 | 0.003 | (**) |
| NP0_1_4&5 | 0.601 | 0.000 | (***) |
| NP0_1_4&6 | 0.408 | 0.000 | (***) |
| NP0_1_5&6 | 0.391 | 0.000 | (***) |
| NM1_1_1&2 | 0.405 | 0.940 |  |
| NM1_1_1&3 | 0.199 | 0.001 | (***) |
| NM1_1_1&4 | 0.232 | 0.000 | (***) |
| NM1_1_1&5 | 0.220 | 0.000 | (***) |
| NM1_1_1&6 | 0.151 | 0.003 | (**) |
| NM1_1_2&3 | 0.388 | 0.126 |  |
| NM1_1_2&4 | 0.403 | 0.054 |  |
| NM1_1_2&5 | 0.343 | 0.045 | (*) |
| NM1_1_2&6 | 0.233 | 0.037 | (*) |
| NM1_1_3&4 | 0.140 | 0.403 |  |
| NM1_1_3&5 | 0.160 | 0.553 |  |
| NM1_1_3&6 | 0.091 | 0.448 |  |
| NM1_1_4&5 | 0.140 | 0.062 |  |
| NM1_1_4&6 | 0.104 | 0.104 |  |
| NM1_1_5&6 | 0.117 | 0.174 |  |
| NV1_1_1&2 | 0.170 | 0.657 |  |
| NV1_1_1&3 | 0.083 | 0.002 | (**) |
| NV1_1_1&4 | 0.083 | 0.000 | (***) |
| NV1_1_1&5 | 0.085 | 0.000 | (***) |
| NV1_1_1&6 | 0.058 | 0.002 | (**) |
| NV1_1_2&3 | 0.172 | 0.139 |  |
| NV1_1_2&4 | 0.139 | 0.251 |  |
| NV1_1_2&5 | 0.138 | 0.158 |  |
| NV1_1_2&6 | 0.110 | 0.042 | (*) |
| NV1_1_3&4 | 0.034 | 0.158 |  |
| NV1_1_3&5 | 0.048 | 0.263 |  |
| NV1_1_3&6 | 0.020 | 0.109 |  |
| NV1_1_4&5 | 0.035 | 0.027 | (*) |
| NV1_1_4&6 | 0.022 | 0.021 | (*) |
| NV1_1_5&6 | 0.030 | 0.044 | (*) |
| NMO_1_1&2 | 0.205 | 0.019 | (*) |
| NMO_1_1&3 | 0.116 | 0.000 | (***) |
| NMO_1_1&4 | 0.138 | 0.000 | (***) |
| NMO_1_1&5 | 0.149 | 0.000 | (***) |
| NMO_1_1&6 | 0.137 | 0.003 | (**) |
| NMO_1_2&3 | 0.131 | 0.938 |  |
| NMO_1_2&4 | 0.139 | 0.950 | (*) |
| NMO_1_2&5 | 0.142 | 0.958 | (*) |
| NMO_1_2&6 | 0.146 | 0.966 | (*) |
| NMO_1_3&4 | 0.051 | 0.991 | (**) |
| NMO_1_3&5 | 0.065 | 0.997 | (**) |
| NMO_1_3&6 | 0.052 | 0.995 | (**) |
| NMO_1_4&5 | 0.056 | 0.990 | (**) |
| NMO_1_4&6 | 0.062 | 0.992 | (**) |
| NMO_1_5&6 | 0.072 | 0.996 | (**) |

**Table S11** ABC model checking for Scenario 5 in network analysis of one reticulation *Brassica rapa* phylogenies. Summary statistics included: the proportion of monomorphic loci (HP0), mean and variance of gene diversity on polymorphic loci (HM1 and HV1 respectively), mean gene diversity on all loci (HMO), proportion of loci with null Fst distances (FP0), mean and variance of non-zero Fst distances (FM1 and FV1 respectively), mean of all Fst distances (FMO), proportion of loci with null Nei’s distances (NP0), mean and variance of non-zero Nei’s distances (NM1 and NV1 respectively) and mean of all Nei’s distances (NMO). Population *j* in summary statistic format STAT_1_*j* corresponds to populations 1:6 in Figure S9. Tail-area probability significance is indicated as *, *P* < 0.05; **, *P* < 0.01; ***, *P* < 0.001.

| Summary statistics | Observed value | proportion (simulated<observed) | |
| --- | --- | --- | --- |
| HP0_1_1 | 0.678 | 0.996 | (**) |
| HP0_1_2 | 0.952 | 0.085 |  |
| HP0_1_3 | 0.774 | 0.013 | (*) |
| HP0_1_4 | 0.761 | 0.002 | (**) |
| HP0_1_5 | 0.678 | 0.002 | (**) |
| HP0_1_6 | 0.432 | 0.002 | (**) |
| HM1_1_1 | 0.395 | 1.000 | (***) |
| HM1_1_2 | 0.443 | 0.877 |  |
| HM1_1_3 | 0.355 | 0.748 |  |
| HM1_1_4 | 0.491 | 0.875 |  |
| HM1_1_5 | 0.367 | 0.635 |  |
| HM1_1_6 | 0.325 | 1.000 | (***) |
| HV1_1_1 | 0.018 | 0.259 |  |
| HV1_1_2 | 0.016 | 0.450 |  |
| HV1_1_3 | 0.014 | 0.000 | (***) |
| HV1_1_4 | 0.009 | 0.160 |  |
| HV1_1_5 | 0.017 | 0.043 | (*) |
| HV1_1_6 | 0.021 | 0.001 | (***) |
| HMO_1_1 | 0.127 | 0.014 | (*) |
| HMO_1_2 | 0.022 | 0.925 |  |
| HMO_1_3 | 0.080 | 0.987 | (*) |
| HMO_1_4 | 0.117 | 1.000 | (***) |
| HMO_1_5 | 0.118 | 0.999 | (***) |
| HMO_1_6 | 0.185 | 1.000 | (***) |
| FP0_1_1&2 | 0.575 | 0.947 |  |
| FP0_1_1&3 | 0.434 | 0.975 | (*) |
| FP0_1_1&4 | 0.485 | 0.375 |  |
| FP0_1_1&5 | 0.361 | 0.919 |  |
| FP0_1_1&6 | 0.210 | 0.106 |  |
| FP0_1_2&3 | 0.681 | 0.128 |  |
| FP0_1_2&4 | 0.665 | 0.019 | (*) |
| FP0_1_2&5 | 0.621 | 0.017 | (*) |
| FP0_1_2&6 | 0.471 | 0.001 | (***) |
| FP0_1_3&4 | 0.770 | 0.005 | (**) |
| FP0_1_3&5 | 0.674 | 0.000 | (***) |
| FP0_1_3&6 | 0.532 | 0.000 | (***) |
| FP0_1_4&5 | 0.775 | 0.002 | (**) |
| FP0_1_4&6 | 0.633 | 0.000 | (***) |
| FP0_1_5&6 | 0.519 | 0.000 | (***) |
| FM1_1_1&2 | 0.558 | 0.894 |  |
| FM1_1_1&3 | 0.304 | 0.002 | (**) |
| FM1_1_1&4 | 0.357 | 0.001 | (***) |
| FM1_1_1&5 | 0.330 | 0.008 | (**) |
| FM1_1_1&6 | 0.301 | 0.015 | (*) |
| FM1_1_2&3 | 0.463 | 0.015 | (*) |
| FM1_1_2&4 | 0.522 | 0.014 | (*) |
| FM1_1_2&5 | 0.434 | 0.010 | (**) |
| FM1_1_2&6 | 0.325 | 0.003 | (**) |
| FM1_1_3&4 | 0.351 | 0.070 |  |
| FM1_1_3&5 | 0.286 | 0.173 |  |
| FM1_1_3&6 | 0.180 | 0.108 |  |
| FM1_1_4&5 | 0.312 | 0.041 | (*) |
| FM1_1_4&6 | 0.218 | 0.000 | (***) |
| FM1_1_5&6 | 0.221 | 0.444 |  |
| FV1_1_1&2 | 0.126 | 0.130 |  |
| FV1_1_1&3 | 0.082 | 0.002 | (**) |
| FV1_1_1&4 | 0.078 | 0.000 | (***) |
| FV1_1_1&5 | 0.081 | 0.002 | (**) |
| FV1_1_1&6 | 0.070 | 0.016 | (*) |
| FV1_1_2&3 | 0.150 | 0.991 | (**) |
| FV1_1_2&4 | 0.105 | 0.879 |  |
| FV1_1_2&5 | 0.120 | 0.664 |  |
| FV1_1_2&6 | 0.104 | 0.020 | (*) |
| FV1_1_3&4 | 0.041 | 0.013 | (*) |
| FV1_1_3&5 | 0.051 | 0.190 |  |
| FV1_1_3&6 | 0.027 | 0.021 | (*) |
| FV1_1_4&5 | 0.044 | 0.025 | (*) |
| FV1_1_4&6 | 0.036 | 0.004 | (**) |
| FV1_1_5&6 | 0.040 | 0.157 |  |
| FMO_1_1&2 | 0.237 | 0.098 |  |
| FMO_1_1&3 | 0.172 | 0.006 | (**) |
| FMO_1_1&4 | 0.185 | 0.025 | (*) |
| FMO_1_1&5 | 0.211 | 0.028 | (*) |
| FMO_1_1&6 | 0.238 | 0.036 | (*) |
| FMO_1_2&3 | 0.147 | 0.101 |  |
| FMO_1_2&4 | 0.175 | 0.189 |  |
| FMO_1_2&5 | 0.164 | 0.168 |  |
| FMO_1_2&6 | 0.172 | 0.241 |  |
| FMO_1_3&4 | 0.081 | 0.961 | (*) |
| FMO_1_3&5 | 0.093 | 0.991 | (**) |
| FMO_1_3&6 | 0.084 | 0.984 | (*) |
| FMO_1_4&5 | 0.070 | 0.938 |  |
| FMO_1_4&6 | 0.080 | 0.972 | (*) |
| FMO_1_5&6 | 0.106 | 0.994 | (**) |
| NP0_1_1&2 | 0.493 | 0.985 | (*) |
| NP0_1_1&3 | 0.419 | 0.988 | (*) |
| NP0_1_1&4 | 0.404 | 0.940 |  |
| NP0_1_1&5 | 0.324 | 0.897 |  |
| NP0_1_1&6 | 0.095 | 0.002 | (**) |
| NP0_1_2&3 | 0.663 | 0.167 |  |
| NP0_1_2&4 | 0.654 | 0.026 | (*) |
| NP0_1_2&5 | 0.585 | 0.017 | (*) |
| NP0_1_2&6 | 0.374 | 0.007 | (**) |
| NP0_1_3&4 | 0.640 | 0.003 | (**) |
| NP0_1_3&5 | 0.598 | 0.002 | (**) |
| NP0_1_3&6 | 0.430 | 0.013 | (*) |
| NP0_1_4&5 | 0.601 | 0.000 | (***) |
| NP0_1_4&6 | 0.408 | 0.001 | (***) |
| NP0_1_5&6 | 0.391 | 0.001 | (***) |
| NM1_1_1&2 | 0.405 | 0.931 |  |
| NM1_1_1&3 | 0.199 | 0.003 | (**) |
| NM1_1_1&4 | 0.232 | 0.002 | (**) |
| NM1_1_1&5 | 0.220 | 0.005 | (**) |
| NM1_1_1&6 | 0.151 | 0.024 | (*) |
| NM1_1_2&3 | 0.388 | 0.025 | (*) |
| NM1_1_2&4 | 0.403 | 0.012 | (*) |
| NM1_1_2&5 | 0.343 | 0.014 | (*) |
| NM1_1_2&6 | 0.233 | 0.030 | (*) |
| NM1_1_3&4 | 0.140 | 0.034 | (*) |
| NM1_1_3&5 | 0.160 | 0.295 |  |
| NM1_1_3&6 | 0.091 | 0.242 |  |
| NM1_1_4&5 | 0.140 | 0.035 | (*) |
| NM1_1_4&6 | 0.104 | 0.062 |  |
| NM1_1_5&6 | 0.117 | 0.520 |  |
| NV1_1_1&2 | 0.170 | 0.677 |  |
| NV1_1_1&3 | 0.083 | 0.003 | (**) |
| NV1_1_1&4 | 0.083 | 0.000 | (***) |
| NV1_1_1&5 | 0.085 | 0.003 | (**) |
| NV1_1_1&6 | 0.058 | 0.017 | (*) |
| NV1_1_2&3 | 0.172 | 0.479 |  |
| NV1_1_2&4 | 0.139 | 0.863 |  |
| NV1_1_2&5 | 0.138 | 0.289 |  |
| NV1_1_2&6 | 0.110 | 0.016 | (*) |
| NV1_1_3&4 | 0.034 | 0.017 | (*) |
| NV1_1_3&5 | 0.048 | 0.224 |  |
| NV1_1_3&6 | 0.020 | 0.048 | (*) |
| NV1_1_4&5 | 0.035 | 0.022 | (*) |
| NV1_1_4&6 | 0.022 | 0.023 | (*) |
| NV1_1_5&6 | 0.030 | 0.244 |  |
| NMO_1_1&2 | 0.205 | 0.100 |  |
| NMO_1_1&3 | 0.116 | 0.002 | (**) |
| NMO_1_1&4 | 0.138 | 0.002 | (**) |
| NMO_1_1&5 | 0.149 | 0.019 | (*) |
| NMO_1_1&6 | 0.137 | 0.032 | (*) |
| NMO_1_2&3 | 0.131 | 0.079 |  |
| NMO_1_2&4 | 0.139 | 0.071 |  |
| NMO_1_2&5 | 0.142 | 0.109 |  |
| NMO_1_2&6 | 0.146 | 0.150 |  |
| NMO_1_3&4 | 0.051 | 0.795 |  |
| NMO_1_3&5 | 0.065 | 0.980 | (*) |
| NMO_1_3&6 | 0.052 | 0.967 | (*) |
| NMO_1_4&5 | 0.056 | 0.860 |  |
| NMO_1_4&6 | 0.062 | 0.923 |  |
| NMO_1_5&6 | 0.072 | 0.987 | (*) |

**Table S29** Descriptions for putative *B. oleracea* – *B. rapa* orthologues (identified as reciprocal best blast pairs) in peaks of positive selection of domesticates with similar phenotypes. Only pairs with AT annotations for at least one gene are listed with their associated biological process gene ontology annotation where available. Genes with similar sequence identity were identified by reciprocal BLAST searches with e-value <1x10^-4^ and >60 % sequence identity.

| **Comparison** | **Pair name** | **Gene ID** | **AT number** | **AT gene name** | **AT gene GO category (biological process)** |
| --- | --- | --- | --- | --- | --- |
| *Brassica oleracea* var. *alboglabra* and  *Brassica rapa* ssp. *parachinensis* | A-P.2 | Bo8g049720 | AT4G17785 | Myb domain protein 39 (MYB39) | regulation of transcription, DNA templated |
|  |  | BraA03g063730 |  |  |  |
|  | A-P.4 | Bo9g018740 |  |  |  |
|  |  | BraA06g022760 | AT1G65960 | Glutamate decarboxylase 2 (GAD2) | glutamate catabolic process, glutamate metabolic process, nitrogen compound metabolic process |
|  | A-P.6 | Bo9g097100 |  |  |  |
|  |  | BraA06g022250 | ATMG00810 | (ORF240B) |  |
| *Brassica oleracea* var. *capitata*  and  *Brassica rapa* ssp. *pekinensis* | C-P.1 | Bo1g028510 | AT4G22670 | HSP70-interacting protein 1 (HIP1) | chaperone cofactor-dependent protein refolding, chaperone-mediated protein folding, protein-containing complex assembly, response to cadmium ion |
|  |  | BraA03g063600 |  |  |  |
|  | C-P.4 | Bo2g098750 | AT2G45750 | S-adenosyl-L-methionine-dependent methyltransferases superfamily protein (PMT16) | methylation |
|  |  | BraA09g032060 | AT1G33170 | S-adenosyl-L-methionine-dependent methyltransferases superfamily protein (PMT18) |  |
|  | C-P.5 | Bo2g099120 | AT4G01440 | Usually multiple acids move in and out transporters 31 (UMAMIT31) |  |
|  |  | BraA03g064700 | AT5G40780 | Lysine histidine transporter 1 (LHT1) | amino acid import, defense response, response to karrikin |
|  | C-P.6 | Bo2g099150 |  |  |  |
|  |  | BraA09g031950 | AT1G33080 | MATE efflux family protein | transmembrane transport |
|  | C-P.7 | Bo4g059840 | AT2G30070 | Potassium transporter (KT1) | potassium ion transmembrane transport, potassium ion transport |
|  |  | BraA09g032790 | AT1G73360 | Homeodomain glabrous 11 (HDG11) | plant-type cell wall loosening, trichome branching |
|  | C-P.8 | Bo4g079890 |  |  |  |
|  |  | BraA03g065040 | AT1G80850 | DNA glycosylase superfamily protein | base-excision repair |
