# Supplementary material for: Extensive crop–wild hybridization during Brassica evolution and selection during the domestication and diversification of Brassica crops
Source: Genetics. 2023 Feb 22;223(4):iyad027. doi: 10.1093/genetics/iyad027 (PMC10078912; doi:10.1093/genetics/iyad027)

Supporting Information

**Article title: Extensive crop-wild hybridisation during Brassica evolution, and selection during the domestication and diversification of Brassica crops**

**Authors:** Jasmine M. Saban, Anne J. Romero, Thomas H. G. Ezard & Mark A. Chapman

**Article acceptance date:**

The following Supporting Information is available for this article:

**Methods S1** Extended materials and methods.

**Figure S1** Maximum likelihood phylogeny of *Brassica* species based on single nucleotide polymorphisms (filtered by linkage disequilibrium) identified by mapping resequencing data of 108 samples to the *Brassica* *oleracea* pangenome.

**Figure S2** Maximum likelihood phylogeny of *Brassica* species based on single nucleotide polymorphisms (filtered by linkage disequilibrium) identified by mapping resequencing data of 77 samples to the *Brassica* *rapa* ssp. *pekinensis* genome.

**Figure S3** Average genome-wide relative minimum distance (RNDmin) between domesticated *Brassica* crops and wild monophyletic *Brassica* species relative to outgroup *Raphanus raphanistrum*.

**Figure S4** Signals of introgression between *Brassica oleracea* varieties and wild *Brassica* relatives as a heatmap of significant (P<0.05, FDR correction) D-statistics.

**Figure S5** Pseudolikelihood of models inferred for zero to five reticulations in phylogenetic network analysis of *Brassica oleracea.*

**Figure S6** Phylogenetic networks identified as having the highest pseudolikelihood for number of reticulations 5:0 (a-f) in analysis of *Brassica oleracea.*

**Figure S7** Signals of introgression between *Brassica rapa* varieties and wild *Brassica* relatives as a heatmap of significant (P<0.05, FDR correction) D-statistics.

**Figure S8** Pseudolikelihood of models inferred for zero to five reticulations in phylogenetic network analysis of *Brassica rapa.*

**Figure S9** The five phylogenetic networks with highest pseudolikelihood for one reticulation in analysis of *Brassica rapa* phylogenies.

**Figure S10** Density distribution of annotation values informing SNP discovery for 108 individual samples aligned to the *Brassica oleracea* pangenome assembly.

**Figure S11** Density distribution of annotation values informing INDEL discovery for 108 individual samples aligned to the *Brassica oleracea* pangenome assembly.

**Figure S12** Density distribution of annotation values informing SNP discovery for 77 individual samples aligned to the *Brassica rapa* v3.0 genome assembly.

**Figure S13** Density distribution of annotation values informing INDEL discovery for 77 individual samples aligned to the *Brassica rapa* v3.0 genome assembly.


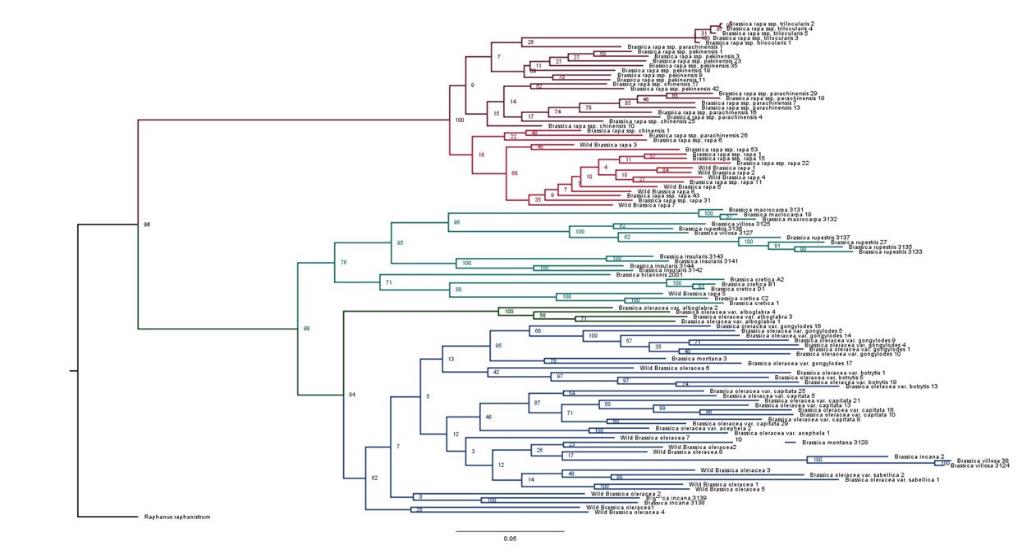
**Figure S1** Maximum likelihood phylogeny of *Brassica* species based on single nucleotide polymorphisms (filtered by linkage disequilibrium) identified by mapping resequencing data of 108 samples to the *Brassica* *oleracea* pangenome.

**Figure S2** Maximum likelihood phylogeny of *Brassica* species based on single nucleotide polymorphisms (filtered by linkage disequilibrium) identified by mapping resequencing data of 77 samples to the *Brassica* *rapa* ssp. *pekinensis* genome.


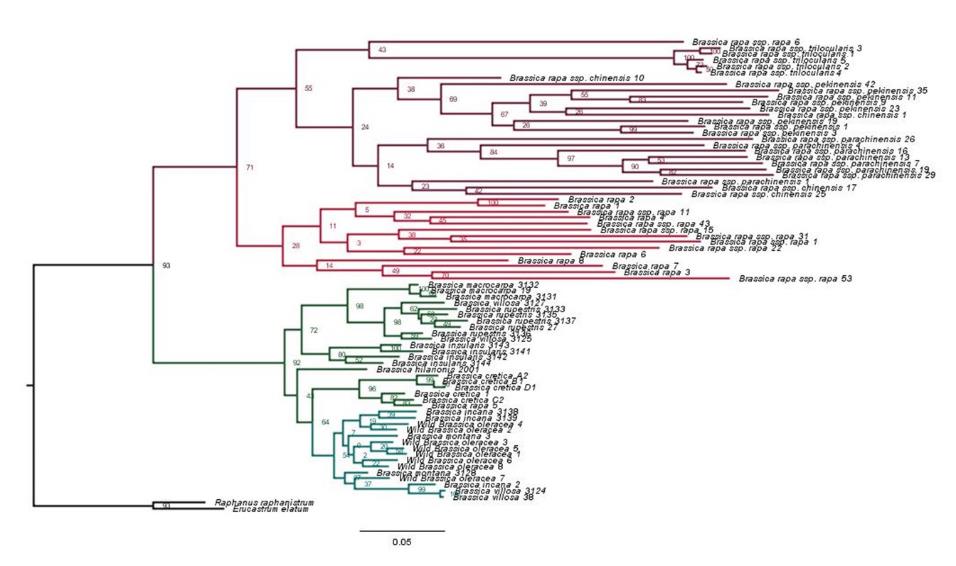


**Figure S3** Average genome-wide relative minimum distance (RNDmin) between domesticated *Brassica* crops and wild monophyletic *Brassica* species relative to outgroup *Raphanus raphanistrum*. Mean RNDmin values with 95 % confidence intervals are plotted for comparisons to a) domesticated *B. oleracea* and b) to domesticated *B. rapa* (excluding ssp. rapa). Pairwise comparisons of the distribution of non-zero RNDmin values were conducted using one-way ANOVA with posthoc Tukey’s HSD test. P-values for comparisons between RNDmin for *Brassica* crops versus the wild relative with smallest RNDmin, and Brassica crops versus each of the other wild relatives are indicated. *For B. oleracea* comparisons (a) p-values indicating the probability of windows with zero RNDmin, calculated using logistic regression, is also visualised for the specified comparisons. Significance is indicated as *, *P* < 0.05; **, *P* < 0.01; ***, *P* < 0.001.

1. **RNDmin between wild *Brassica* species and domesticated *B. rapa***
2. **RNDmin between wild *Brassica* species and domesticated *B. oleracea***

**
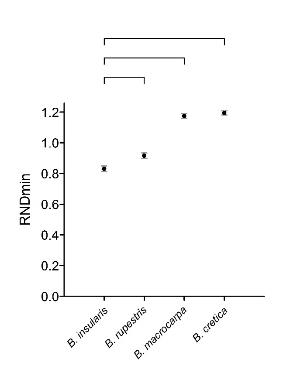

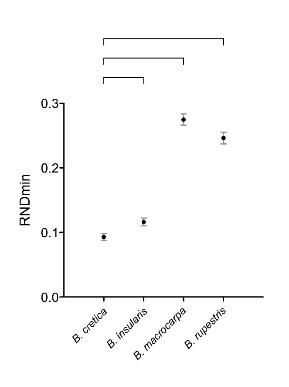
**

∗∗∗

∗∗∗

∗∗∗

∗∗∗ (∗∗∗)

∗∗∗ (∗∗∗)

∗∗∗ (∗)

**Figure S4** Signals of introgression between *Brassica oleracea* varieties and wild *Brassica* relatives as a heatmap of significant D-statistics (P<0.05, Benjamini- Hochberg FDR correction). For each pairwise combination of populations in positions P2 and P3, the corresponding square is coloured according to the D-statistic (strength of signal of introgression) with lowest p-value across all populations in position P1.

*B. oleracea* var. *capitata*

*B. oleracea* var. *botrytis*

*B. oleracea* var. *gongylodes*

*B. oleracea* var. *alboglabra*

Wild *B. oleracea 1*

*B. oleracea* var. *gongylodes*

*B. oleracea* var. *botrytis*

Wild *B. oleracea 2*

*B. oleracea*  var. *alboglabra*

*B. cretica*

*B. insularis*

Wild *B. oleracea* 2

Wild *B. oleracea* 1

*B. macrocarpa*

*B. cretica*

*B. macrocarpa*

*B. insularis*

*B. rupestris*

0

1

0.5

D-stat

**Figure S5** Pseudolikelihood of models inferred for zero to five reticulations in phylogenetic network analysis of *Brassica oleracea.* 18 representative individuals were used to construct networks with Maximum Pseudolikelihood in PhyloNet. The five models with the highest pseudolikelihood for each number of reticulations is plotted.

**
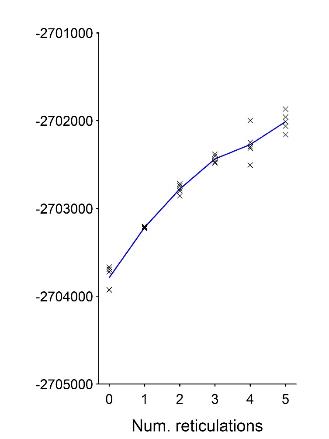
**

Pseudolikelihood

**Figure S6** Phylogenetic networks identified as having the highest pseudolikelihood for number of reticulations 5:0 (a-f) in analysis of *Brassica oleracea.* Populations are abbreviated as ALB: B. oleracea var. alboglabra, BOT: B. oleracea var. botrytis, GON: B. oleracea var. gongylodes, CAP: B. oleracea var. capitata, WB1: Wild B. oleracea group 1, WB2: Wild B. oleracea group 2.


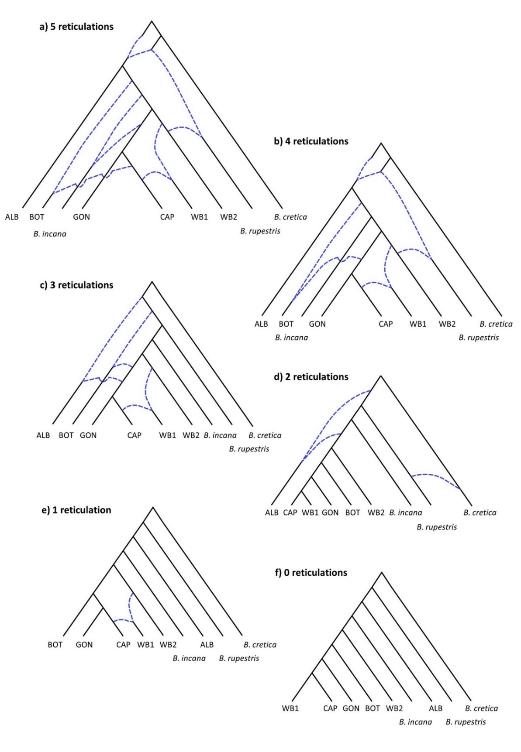


**Figure S7** Signals of introgression between *Brassica rapa* subspecies and wild *Brassica* relatives as a heatmap of significant D-statistics (P<0.05, Benjamini- Hochberg FDR correction). For each pairwise combination of populations in positions P2 and P3, the corresponding square is coloured according to the D-statistic (strength of signal of introgression) with lowest p-value across all populations in position P1.

*B. rapa* ssp. *chinensis*

*B. rapa* ssp. *parachinensis*

*B. rapa* ssp. *pekinensis*

*B. rapa* ssp. *trilocularis*

Wild *B. rapa* and *B. rapa* ssp. *rapa*

*B. cretica*

*B. rapa* ssp. *pekinensis*

*B. rapa* ssp. *parachinensis*

*B. insularis*

*B. macrocarpa*

*B. rapa* ssp. *trilocularis*

*B. cretica*

Wild *B. rapa* and *B. rapa* ssp. *rapa*

*B. insularis*

*B. macrocarpa*

*B. rupestris*

0

1

0.5

D-stat

**Figure S8** Pseudolikelihood of models inferred for zero to five reticulations in phylogenetic network analysis of *Brassica rapa.* 22 representative individuals were used to construct networks with Maximum Pseudolikelihood in PhyloNet. The five models with the highest pseudolikelihood for each number of reticulations is plotted.


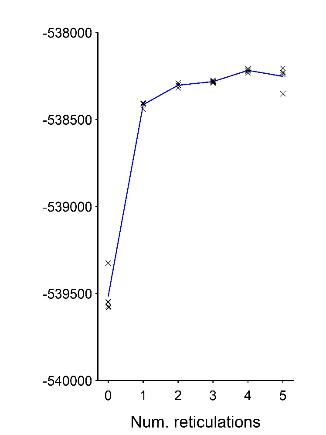


Pseudolikelihood

**Figure S9** The five phylogenetic networks with highest pseudolikelihood for one reticulation in analysis of *Brassica rapa* phylogenies. Populations are abbreviated as WBR: Wild B. rapa and B. rapa ssp. rapa group, PEK: B. rapa ssp. pekinensis, CHI: B. rapa ssp. chinensis, PAR: B.rapa ssp. parachinensis, TRI: B. rapa ssp. trilocularis.

**
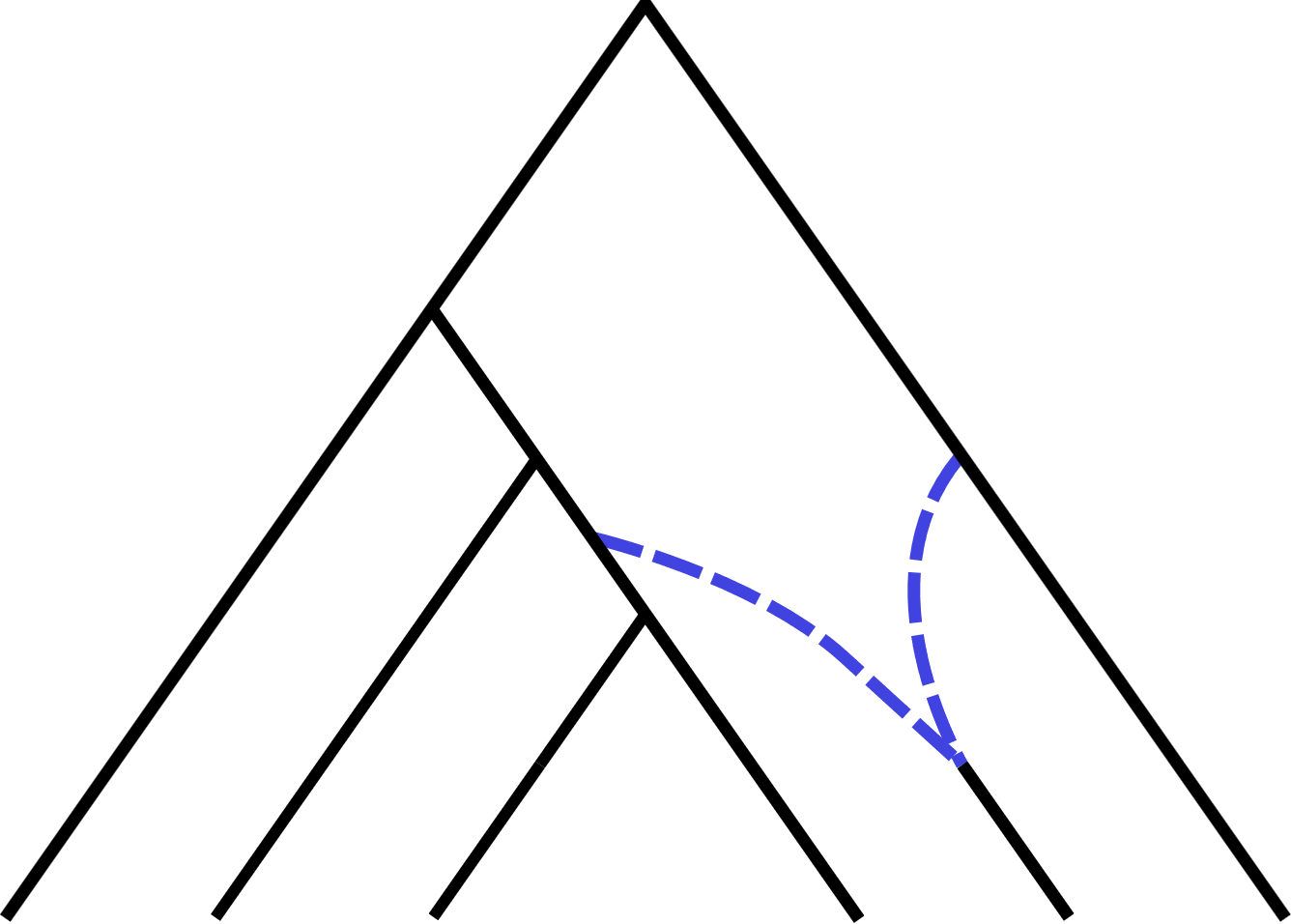

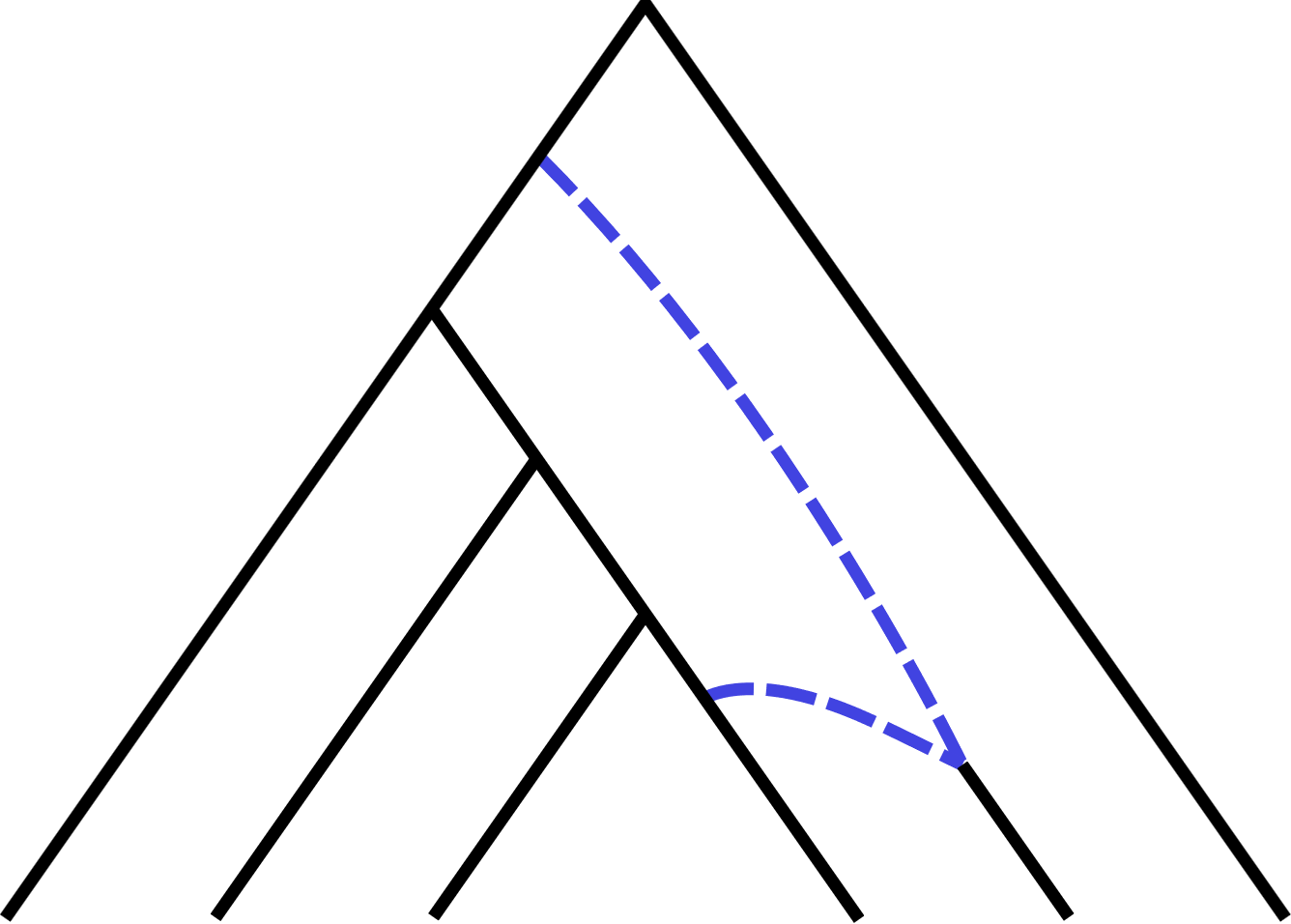

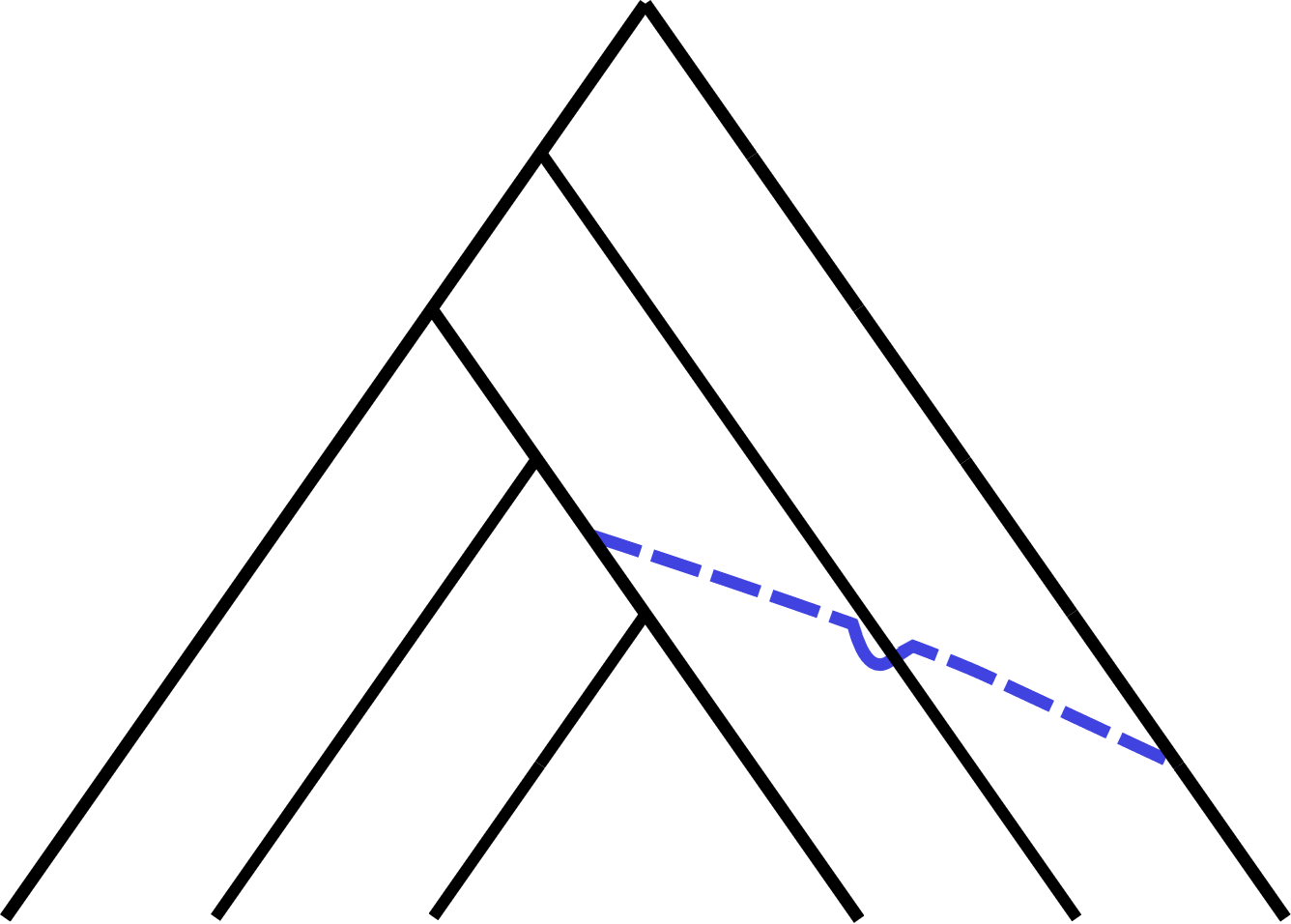

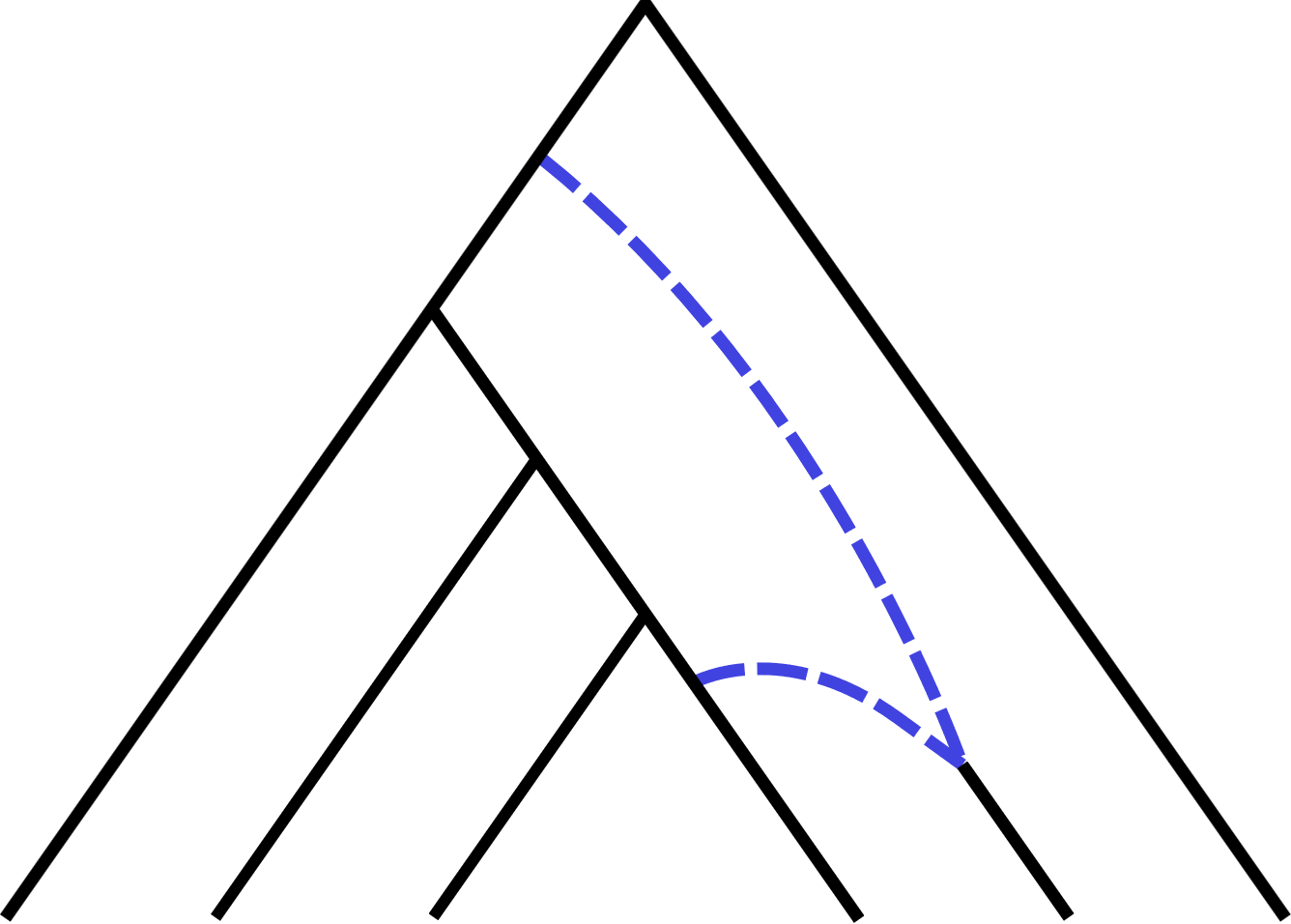

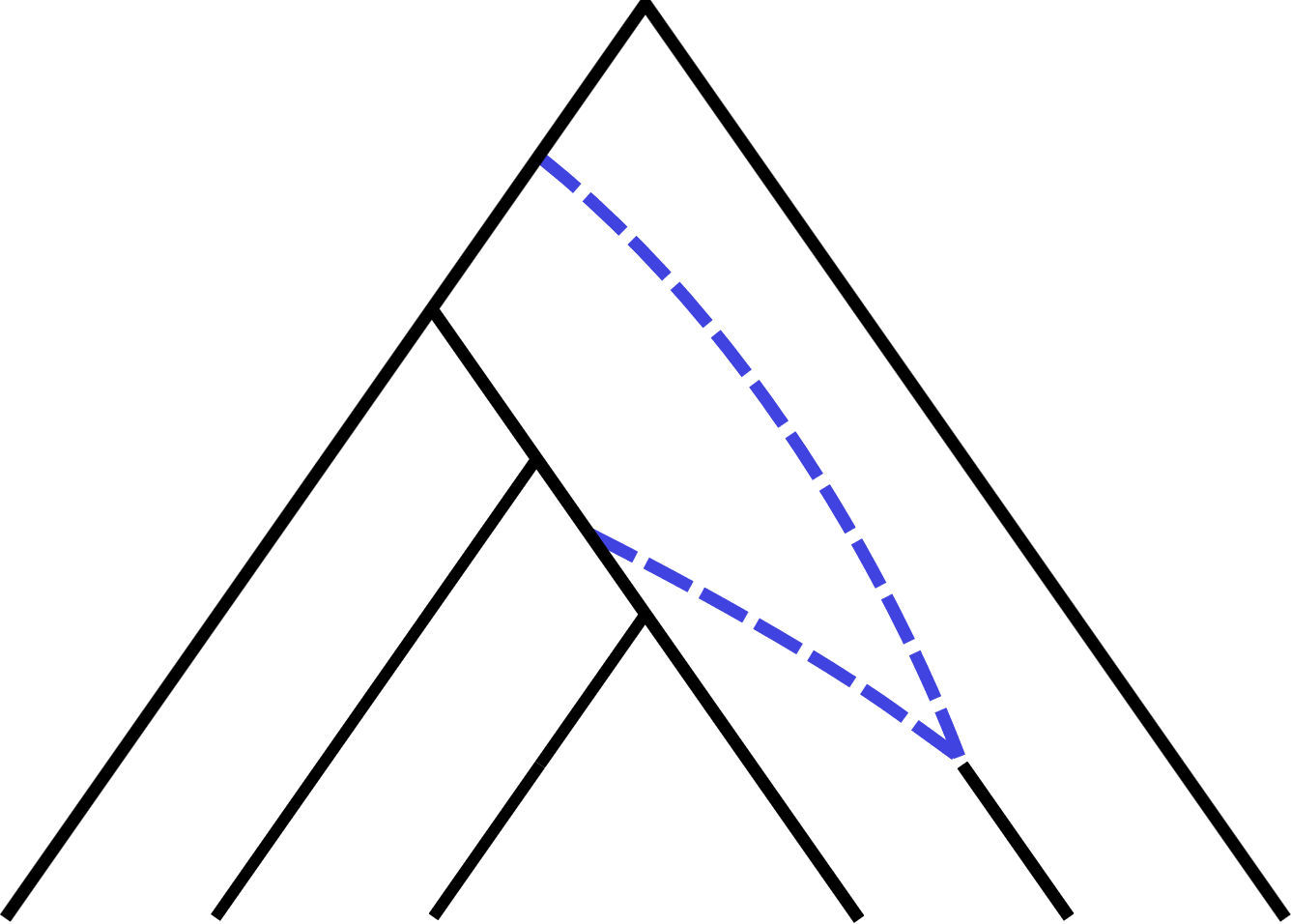
**

**a) Scenario 1**

**b) Scenario 2**

**c) Scenario 3**

**e) Scenario 5**

**d) Scenario 4**

TRI

PEK

WBR

CHI

PAR

*B. cretica*

TRI

PEK

WBR

PAR

CHI

*B. cretica*

TRI

PEK

WBR

CHI

PAR

*B. cretica*

TRI

PEK

WBR

CHI

PAR

*B. cretica*

TRI

PEK

WBR

CHI

PAR

*B. cretica*

**Figure S10** Density distribution of annotation values informing SNP discovery for 108 individual samples aligned to the *Brassica oleracea* pangenome assembly.


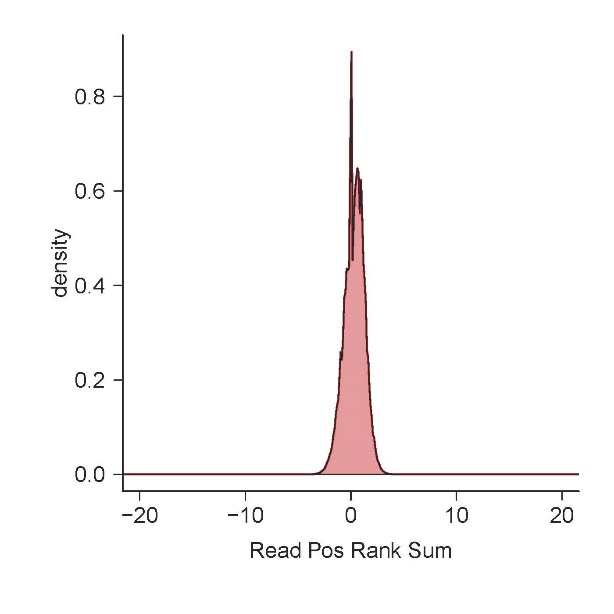

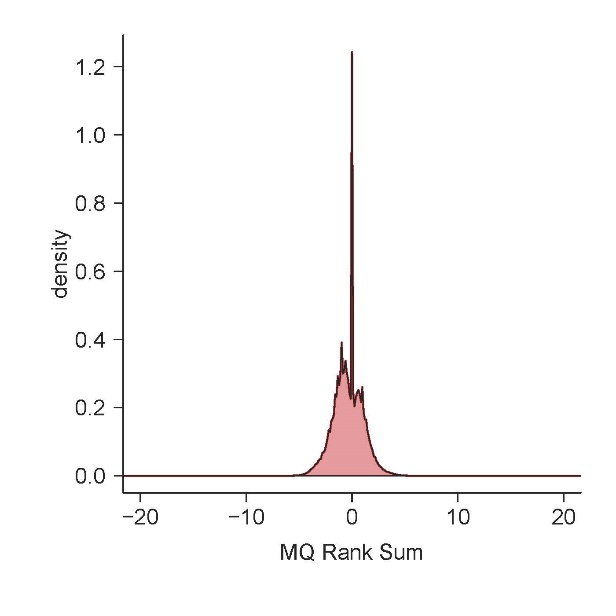

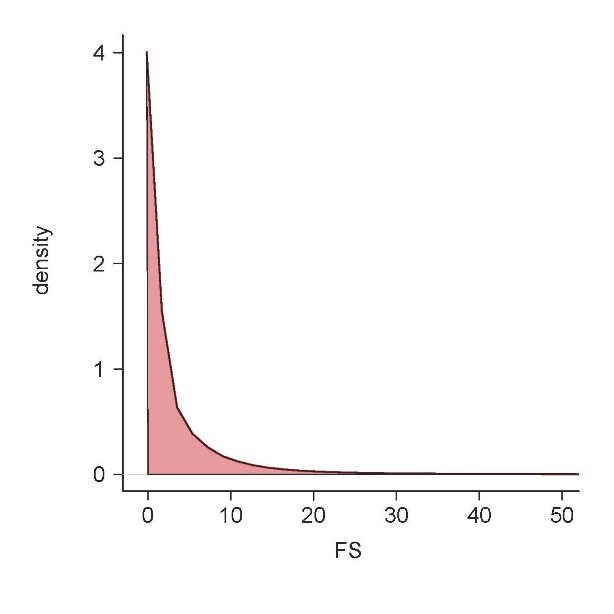

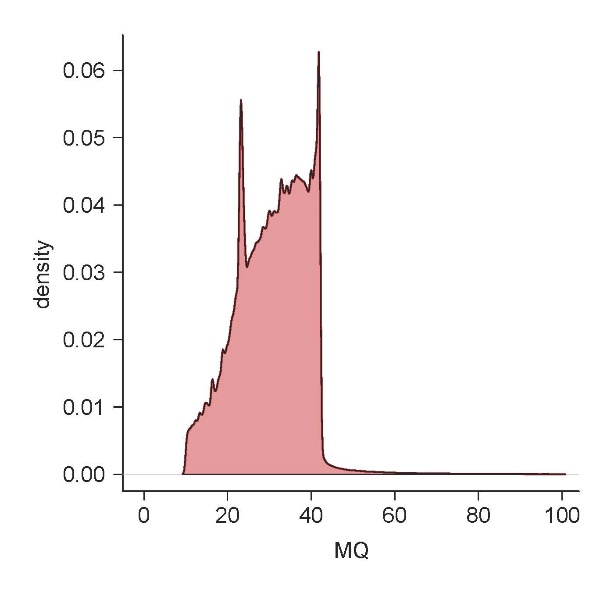

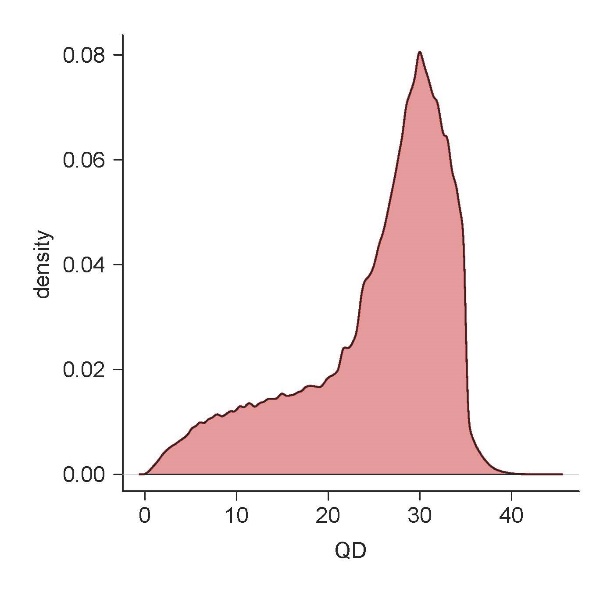

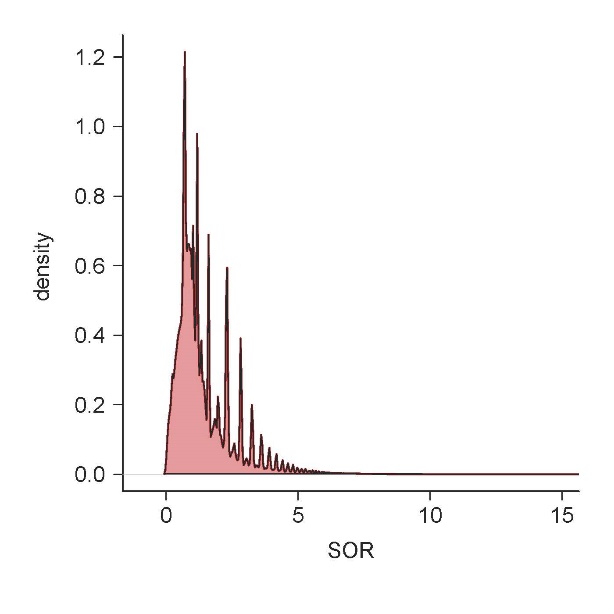


**Figure S11** Density distribution of annotation values informing INDEL discovery for 108 individual samples aligned to the *Brassica oleracea* pangenome assembly.


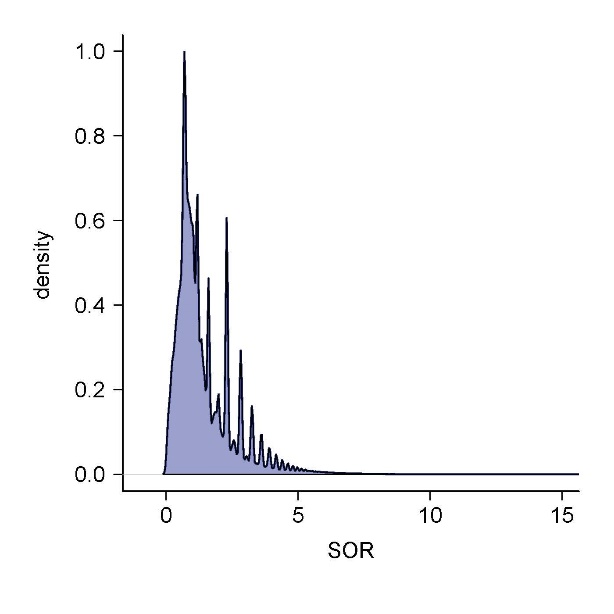

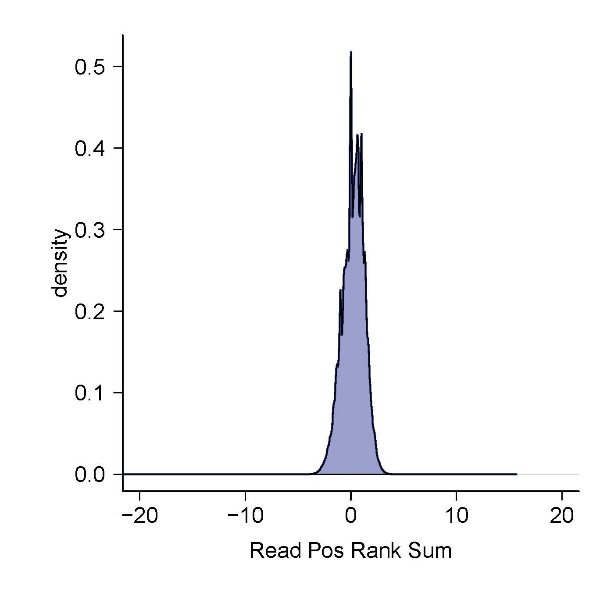

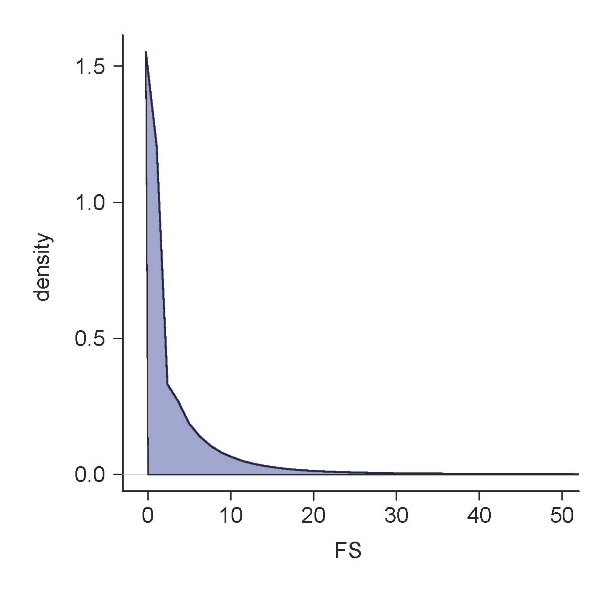

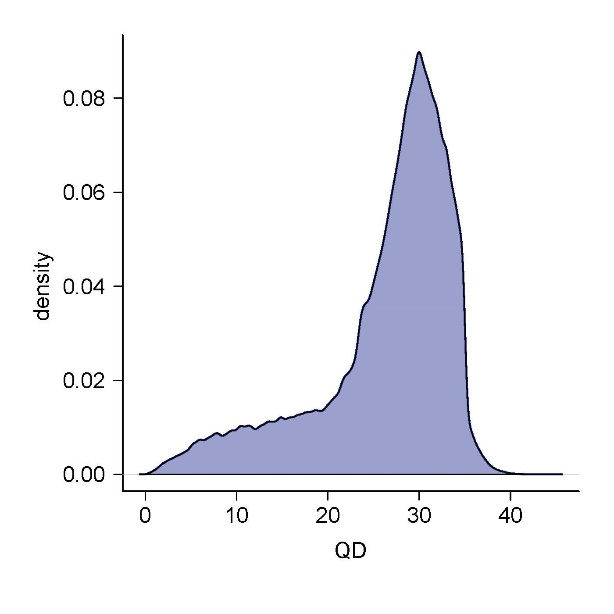

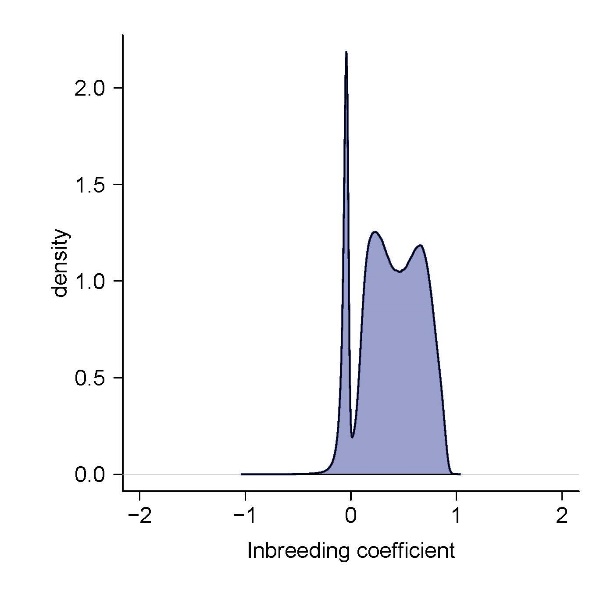


**Figure S12** Density distribution of annotation values informing SNP discovery for 77 individual samples aligned to the *Brassica rapa* v3.0 genome assembly.


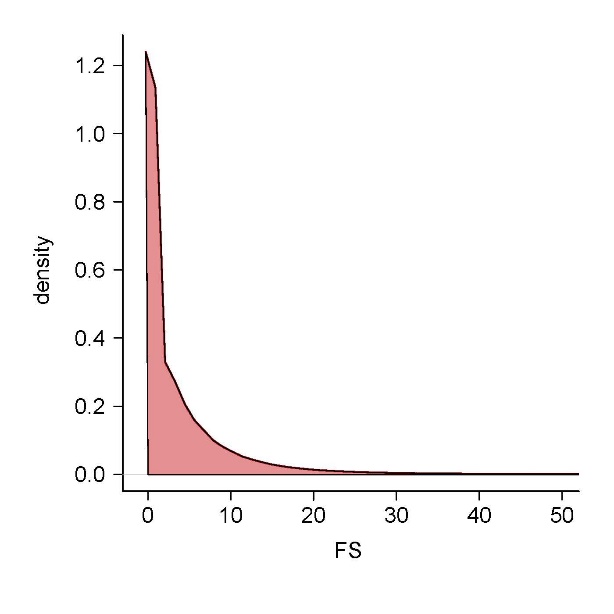

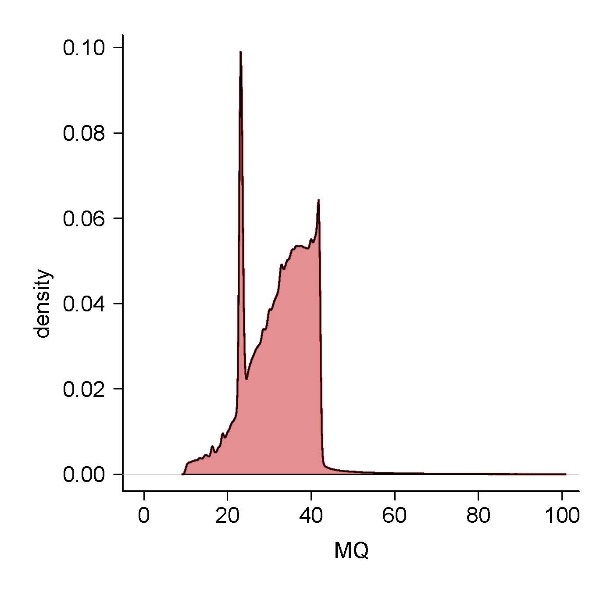

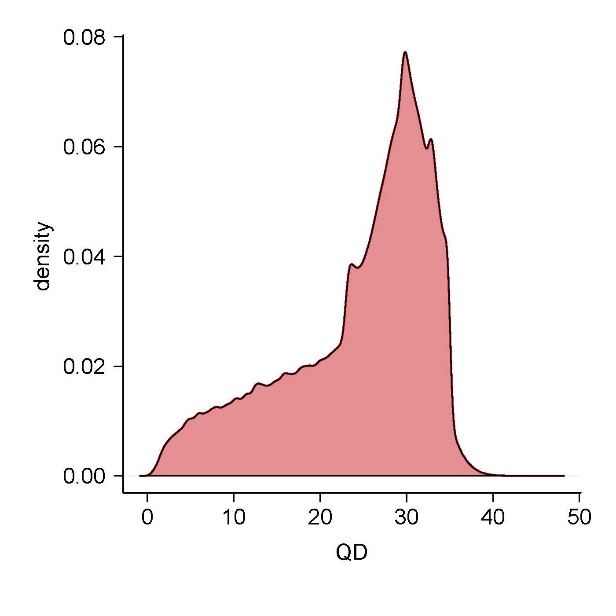

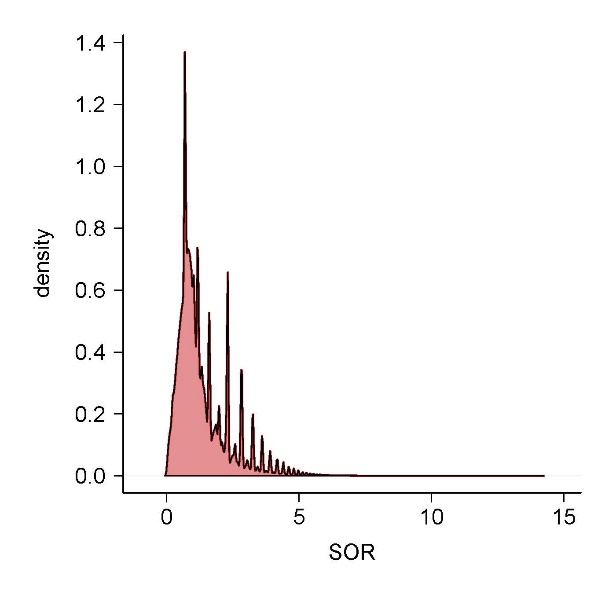

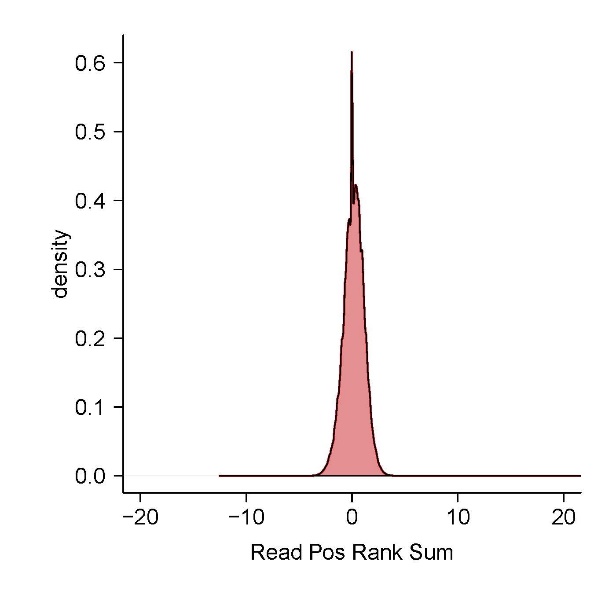

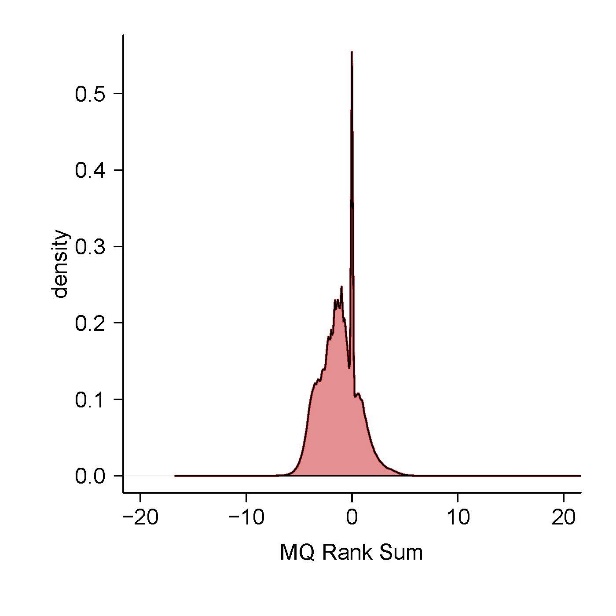


**Figure S13** Density distribution of annotation values informing INDEL discovery for 77 individual samples aligned to the *Brassica rapa* v3.0 genome assembly.


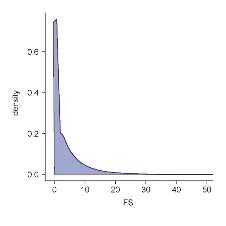

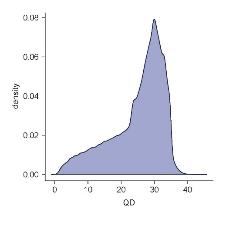


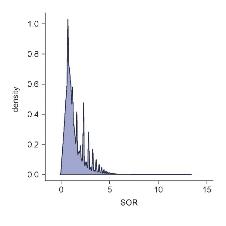

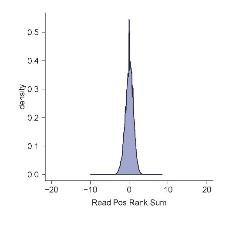


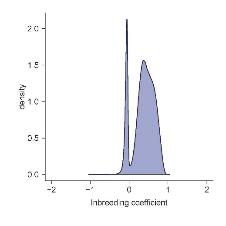

Supplement: iyad027_Supplementary_Data [file iyad027_supplementary_data.zip › Supplemental_Figures_GENETICS-2022-305730.docx]
